# Epigenetic inheritance is gated by naïve pluripotency and *Dppa2*

**DOI:** 10.15252/embj.2021108677

**Published:** 2022-02-24

**Authors:** Valentina Carlini, Cristina Policarpi, Jamie A Hackett

**Affiliations:** ^1^ Epigenetics & Neurobiology Unit European Molecular Biology Laboratory (EMBL) Rome Italy; ^2^ Faculty of Biosciences Collaboration for Joint PhD Degree between EMBL and Heidelberg University Heidelberg Germany

**Keywords:** heterochromatin, dCas9, epigenetic editing, *p53*, X‐inactivation, Chromatin, Transcription & Genomics, Development, Stem Cells & Regenerative Medicine

## Abstract

Environmental factors can trigger cellular responses that propagate across mitosis or even generations. Perturbations to the epigenome could underpin such acquired changes, however, the extent and contexts in which modified chromatin states confer heritable memory in mammals is unclear. Here, we exploit a precision epigenetic editing strategy and forced *Xist* activity to programme *de novo* heterochromatin domains (epialleles) at endogenous loci and track their inheritance in a developmental model. We find that naïve pluripotent phases systematically erase ectopic domains of heterochromatin via active mechanisms, which likely acts as an intergenerational safeguard against transmission of epialleles. Upon lineage specification, however, acquired chromatin states can be probabilistically inherited under selectively favourable conditions, including propagation of *p53* silencing through *in vivo* development. Using genome‐wide CRISPR screening, we identify molecular factors that restrict heritable memory of epialleles in naïve pluripotent cells, and demonstrate that removal of chromatin factor *Dppa2* unlocks the potential for epigenetic inheritance uncoupled from DNA sequence. Our study outlines a mechanistic basis for how epigenetic inheritance is constrained in mammals, and reveals genomic and developmental contexts in which heritable memory is feasible.

## Graphical abstract

Naïve pluripotency and chromatin factor DPPA2 protect against epiallele propagation.

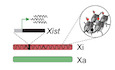

## Introduction

Cellular identity is maintained by the constellation of trans‐ and cis‐acting factors that regulate gene expression programmes. Among these, epigenetic mechanisms, including histone modifications and DNA methylation, play a key role in establishing and perpetuating transcription states during development (Atlasi & Stunnenberg, 2017; Grosswendt *et al*, 2020). For example, heterochromatin domains facilitate stable transcriptional silencing, and are characterised by repressive H3K9me3 and DNA methylation or by H3K27me3 (Allshire & Madhani, 2018). Once established, DNA methylation patterns propagate through cell divisions via the maintenance methylase DNMT1, while histone modifications, such as H3K27me3 and H3K9me3, utilise self‐reinforcing feedback loops (Smith & Meissner, 2013; Reinberg & Vales, 2018). These entail “read‐write” modules that associate with the replication fork to reinstate modification patterns and mutual cross‐talk between epigenetic systems, which together are thought to promote stable “epigenetic” inheritance. Nevertheless, chromatin marks are also subject to active reversal mechanisms and imperfect maintenance during replication, and are consequently rendered in a dynamic equilibrium of opposing influences (Stewart‐Morgan *et al*, 2020). Thus, while chromatin states can convey a degree of heritable memory through reinforcing loops, they also exhibit plasticity in response to extrinsic cues.

These dual properties have implicated epigenetic systems as potential mechanisms that underlie genome–environment interactions (Cavalli & Heard, 2019). Indeed, across phyla and model organisms, environmental changes can induce specific epigenetic alterations—known as epialleles—that drive major phenotypic responses and adaptations (Seong *et al*, 2011; Simola *et al*, 2016; Jiang & Berger, 2017; Yang *et al*, 2017; Ge *et al*, 2018; Duempelmann *et al*, 2019; Torres‐Garcia *et al*, 2020). In mammals, emergent phenotypes have also been linked with chromatin changes as a response to environmental contexts, for example, hypoxia (Batie *et al*, 2019; Chakraborty *et al*, 2019) or availability of metabolic intermediates (Haws *et al*, 2020). Chromatin perturbations more generally are additionally associated with human disease susceptibility (Feinberg, 2018; Panzeri & Pospisilik, 2018). Understanding the potential prevalence and heritability of epialleles (or epimutations) in mammals is therefore of great interest.

Early embryogenesis is considered a susceptibility window for induction of epialleles (Cavalli & Heard, 2019; Bertozzi & Ferguson‐Smith, 2020). Importantly, if epigenetic perturbations occur during development they have the potential to be inherited throughout adult tissues, possibly influencing disease risk (Walker & Shuk‐mei, 2012; Hitchins, 2015). Furthermore, evidence is accruing across model organisms that adverse environments even prior to conception can induce epigenetic perturbations that are intergenerationally inherited, and influence offspring phenotype (Carone *et al*, 2010; Ost *et al*, 2014; Rechavi *et al*, 2014; Huypens *et al*, 2016; Ciabrelli *et al*, 2017; Klosin *et al*, 2017). However, in mammals, pre‐implantation development entails reprogramming of parentally inherited epigenomes, including rewiring of chromatin and global DNA demethylation (Hackett & Surani, 2013). While this reprogramming is often believed to be directly linked with—or even prerequisite for—emergence of naïve pluripotency, epigenome reprogramming could alternatively function as a barrier to inheritance of acquired or ectopic chromatin states (epialleles) (Kazachenka *et al*, 2018). In any case, the potential for heritable epialleles in mammals and the underlying mechanisms that enable or antagonise this during development are relatively uncharacterised.

The advent of epigenome editing tools has provided a means to programme precise epigenetic perturbations at regulatory loci that can model environmentally induced epialleles. Previous reports have shown that targeted H3K9me3 and DNA methylation, or Polycomb, can exhibit stable propagation (Hathaway *et al*, 2012; Amabile *et al*, 2016; Bintu *et al*, 2016; Saunderson *et al*, 2017; Moussa *et al*, 2019; O'Geen *et al*, 2019; Nunez *et al*, 2021), however, these studies do not extend *in vivo*, generally involve epigenetically abnormal cancer cell lines, and/or manipulate the underlying genetic context, while other studies found contradictory results (Kungulovski *et al*, 2015; Braun *et al*, 2017; Policarpi *et al*, 2021). Thus, the potential for epigenetic inheritance at endogenous loci in normal developmental contexts is unresolved. By exploiting an optimised and releasable CRISPR‐dCas9 epigenetic editing tool to deposit broad heterochromatin domains, we reveal that developmental phases of naïve pluripotency function as a blockade to heritable silencing memory in mammals. Coupling our epigenetic memory assay with genome‐wide genetic screens, we pinpoint *Dppa2* as a key “surveyor” that restricts inheritance of epialleles in naïve cells at specific loci. During subsequent developmental transitions, however, inheritance of induced epialleles is supported both *in vitro* and *in vivo*. The results reveal heritable memory of induced chromatin states is viable in differentiated contexts, which has implications for disease risk, but places naïve pluripotency and *Dppa2* as an intergenerational safeguard against epiallele propagation in mammals.

## Results

### A dynamic traceable system to programme *de novo* epialleles

**Figure 1:**
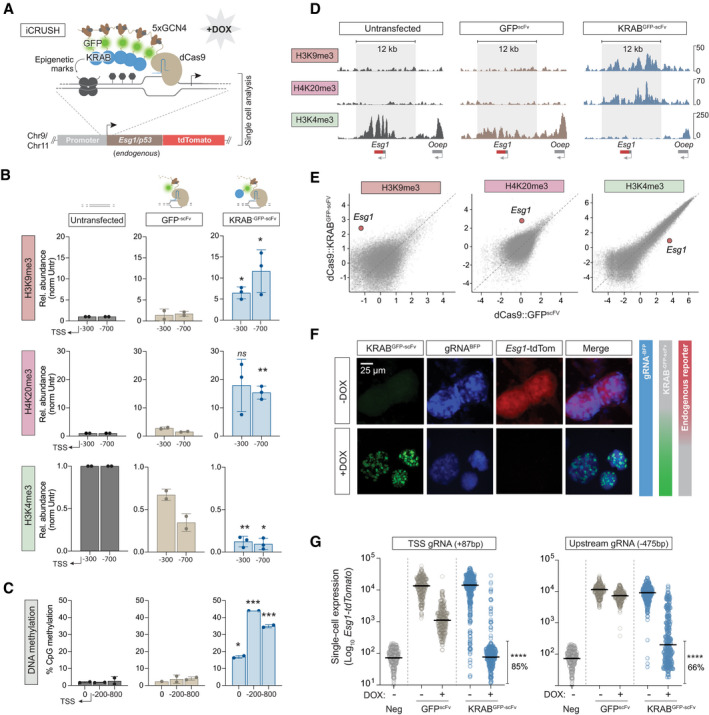
Programming an epiallele promotes a robust heterochromatin domain and gene silencing Schematic showing recruitment of the modular epigenetic editing system to an endogenous promoter upon addition of DOX. A knock‐in reporter downstream facilitates single‐cell analysis.The relative abundance of indicated histone modifications assayed by CUT&RUN‐qPCR after KRAB^GFP‐scFv^ (blue) or control GFP^scFv^ (light brown) targeting, relative to untransfected (grey). Shown are independent quantifications at two genomic positions on the *Esg1* promoter, indicated relative to TSS (−300 bp, −700 bp). Data are mean of two or three independent biological replicates.Histograms showing the average DNA methylation across three genomic regions of the *Esg1* promoter determined by bisulphite pyrosequencing in two biological replicates.CUT&RUN genome tracks for H3K4me3, H4K20me3 and H3K4me3 in untransfected (grey), control GFP^scFv^ (light brown) or KRAB^GFP‐scFv^ (blue) targeted ESC after 7 days of DOX treatment. Grey box highlights the region of heterochromatin spreading induced by epigenetic editing.Scatterplots demonstrating specificity and magnitude of programmed modifications at *Esg1* by CUT&RUN‐seq. Shown are all promoters genome wide.Epifluorescence images of *Esg1‐tdTomato* ESC in −DOX (top) or +DOX conditions (bottom), where targeted heterochromatin is deposited.Single‐cell expression of *Esg1‐tdTomato* in control (GFP^scFv^) or upon heterochromatin induction (KRAB^GFP‐scFv^) using a gRNA targeting close to the TSS (+87 bp) or further upstream on the promoter (−475 bp). Each data point indicates a cell, percentage indicates the proportion of fully silenced cells (mean of four biological replicates) and bars represent median. *P*‐values calculated by comparing +DOX with −DOX conditions. Data information: In all panels, asterisks indicate *P‐*values by unpaired *t*‐test over biological replicates; *P < 0.05, **P < 0.01, ***P < 0.001. Error bars ± SD.

To investigate the potential for memory of *de novo* epigenetic states, we first developed an optimised CRISPR‐based epigenetic programming tool. Here, we employed a catalytically dead (d)Cas9 fused with an array of five optimally spaced GCN4 repeats (dCas9^GCN4^) (Morita *et al*, 2016). These serve as docking sites to recruit up to five “effectors” to a specific genomic locus via their single‐chain antibody (scFv) domain (Fig 1A). This modular system amplifies both the quantitative level and domain size of ON‐target epigenome editing, relative to dCas9 effector fusions, while minimising OFF‐target effects (Pflueger *et al*, 2018). To target *de novo* heterochromatin, we generated KRAB^GFP‐scFv^ and DNMT3A/3L^GFP‐scFv^ effectors, which promote direct deposition of H3K9me3 and DNA methylation respectively (Quenneville *et al*, 2012).

**Figure EV1:**
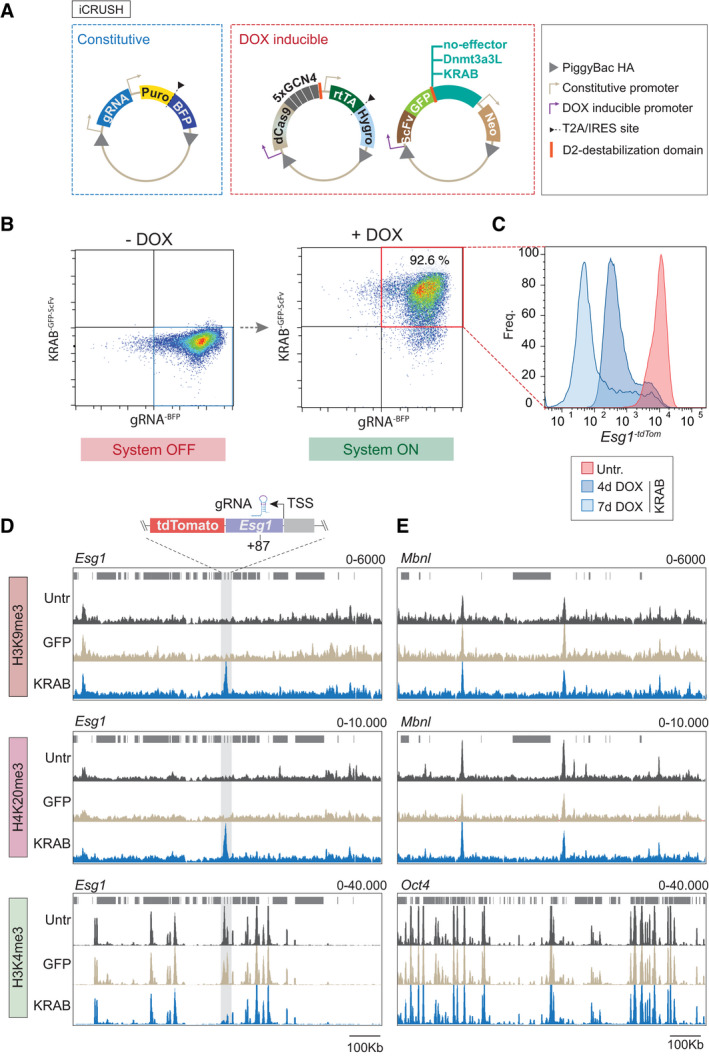
iCRUSH induces domains of *de novo* histone modification levels comparable to endogenous heterochromatin loci APiggyBac constructs used to deliver the epigenetic editing tool (iCRUSH) into ESC. From left to right: the enhanced gRNA scaffold is under control of a constitutive U6 promoter, the same construct also drives constitutive expression of a puromycin selection gene and blue fluorescent protein (BFP) separated by the self‐cleaving peptide T2A; the dCas9^GCN4^ fusion gene is under control of the TRE3G DOX‐inducible promoter, the same construct also drives constitutive expression of the rtTA trans‐activator and a hygromycin resistance gene separated by a T2A; the epigenetic effector constructs (KRAB^GFP‐scFv^ or Dnmt3a3L^GFP‐scFv^ ) are also under control of the TRE3G DOX‐inducible promoter and it is fused with a green fluorescent protein (GFP) and an scFv domain specific for GCN4, it also drives constitutive expression of the neomycin resistance gene. These constructs are genomically integrated into ESC by co‐transfection with the PiggyBac transposase.BDensity plots, obtained by flow cytometry analysis, show gRNA^BFP^ and KRAB^GFP‐scFv^ expression in the cell population prior to and upon DOX induction, revealing its dynamic activation.CFluorescence distribution measured by flow cytometry showing *Esg1* reporter silencing after recruitment of KRAB^GFP‐scFv^ for 4 or 7 days (blue) compared to a reference untargeted sample (red), demonstrating order of magnitude of silencing. Cells were gated for the presence of both KRAB^scFv‐GFP^ (GFP positive) and gRNA^‐BFP^ (BFP positive).D, EGenome views comparing the magnitude of *de novo* peaks of histone marks deposited by iCRUSH at the *Esg1* promoter (D) with endogenous representative examples (*Mbnl* and *Oct4*) (E).

We placed all components under a DOX‐inducible promoter and destabilised dCAS9^GCN4^ protein and effectors with d2 domains, which together facilitate precise temporal control over epigenome editing activity. This is important to assess subsequent epigenetic memory without confounding reiterative targeting. To track the temporal ON‐OFF dynamics in real‐time, all effectors are tagged with superfolder GFP, which also enables cell isolation via flow cytometry (Fig EV1A–C). Finally, we used an “enhanced” gRNA scaffold (AT‐flip, extended stem loop) linked with a tagBFP (Chen *et al*, 2013), which further amplifies ON‐target activity and facilitates tracking respectively. In summary, dCas9^GCN4^ and KRAB^GFP‐scFv^ expression can be induced by DOX treatment and traced in real‐time via GFP, while BFP is constitutively expressed. Reciprocally, the system is destabilised and can be rapidly switched back OFF by removal of DOX.

### Programmed heterochromatin epialleles fully silence gene activity in single cells

To follow programmed epialleles at endogenous loci, we initially used a naïve pluripotent ESC line wherein the endogenous *Esg1* gene carries a knock‐in tdTomato (Fig 1A) (Hackett *et al*, 2018). We introduced dCas9^GCN4^::KRAB^GFP‐scFv^ and a single gRNA^BFP^ that targets the *Esg1* promoter (+87 bp of TSS) via piggyBac, and assessed the extent of *de novo* programmed epigenetic states after 7 days (7 d) induction with DOX. Quantitative CUT&RUN qPCR demonstrated a highly significant deposition of heterochromatic H3K9me3 (*P = 0.014*) marks across the *Esg1* promoter specifically with KRAB^GFP‐ScFv^, relative to either untransfected or control GFP^scFv^ targeting (Fig 1B). This was paralleled by enrichment of another heterochromatic mark, H4K20me3 (*P = 0.002*), which often co‐localises with H3K9me3 (Schotta *et al*, 2004), and complete loss of endogenous H3K4me3 modification (*P* = 0.0013). Moreover, bisulphite pyrosequencing revealed a highly significant increase in DNA methylation (*P* = 0.0005) across the entire *Esg1* promoter region (Fig 1C). These results indicate that upon single‐gRNA tethering of a flexible array of five KRAB^GFP‐scFv^ effectors, a *de novo* domain of heterochromatic modifications is established.

To further investigate the extent and specificity of programmed heterochromatin we performed CUT&RUN‐seq. We observed that our epigenetic editing system deposits a broad domain encompassing ~12 kb of H3K9me3 and H4K20me3 around the endogenous *Esg1* locus, while previously abundant H3K4me3 is undetectable (Fig 1D). Importantly the *de novo* peaks of H3K9me3 and H4K20me3 are of a magnitude comparable to the strongest peaks throughout the genome, suggesting they recapitulate robust physiological heterochromatin status (Figs 1E and EV1D). Moreover, targeting was highly specific, since we observed minimal OFF‐target changes in H3K9me3, H4K20me3 and H3K4me3 (Figs 1E and EV1D). Taken together, these data reveal that a substantial epigenomic domain (> 10 kb), which bears the key hallmarks of repressive heterochromatin, is specifically programmed at an endogenous genomic locus.

We next asked whether this *de novo* heterochromatin domain is associated with induction of transcriptional silencing. *Esg1* is highly active in pluripotent cells, and the endogenous reporter facilitates dynamic single‐cell analysis over time. As expected, addition of DOX led most cells to become GFP positive, indicative of activation of the epigenetic editing system (Fig 1F). Strikingly, this concomitantly led to complete loss of *Esg1* ^tdTomato^‐positive cells, which was additive with time (Figs 1F and EV1C). Quantitative single‐cell expression revealed > 99% cells exhibited transcriptional repression, with > 85% reaching a complete OFF state, indistinguishable from ESC that do not carry the tdTomato reporter (Neg), and corresponding to > 500‐fold transcriptional silencing (Fig 1G). In contrast, the GFP^scFv^ control exhibited only modest repression, which likely reflected steric hindrance due to TSS binding, since a gRNA targeting upstream (−475 bp) elicited full silencing with KRAB^GFP‐scFv^ (> 500 fold) but its control GFP^scFv^ exhibited no effect on transcription (Fig 1G). In summary, these data indicate that our system is able to ectopically programme heterochromatin states at *Esg1*, which is directly linked with powerful transcriptional silencing at the single‐cell level, implying high penetrance of deposition across a broad domain. We refer to this enhanced epigenetic tool as inducible CRISPR unleashing of silencing by heterochromatin (iCRUSH) (Fig 1A).

### Induced heterochromatin is progressively erased in ESC

**Figure 2:**
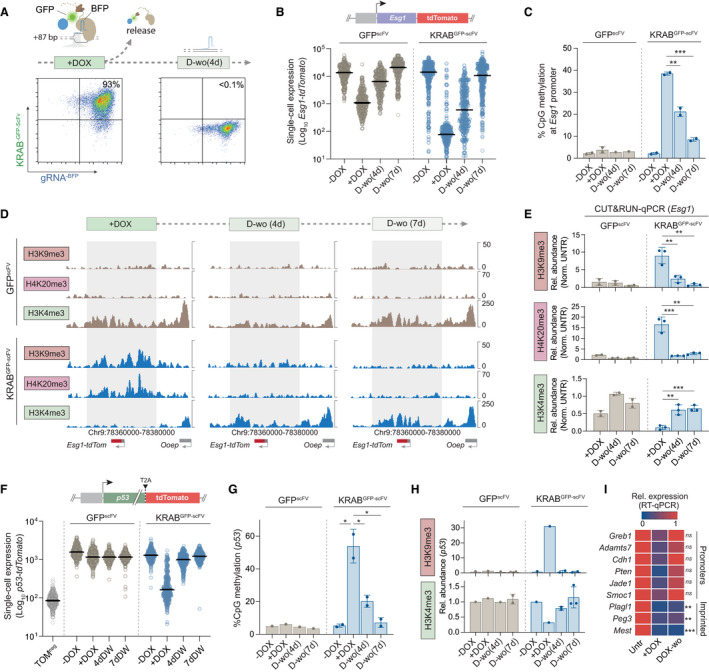
Induced heterochromatin epialleles are progressively erased in naïve pluripotent cells Representative flow cytometry density plots showing gRNA^BFP^ (+87 bp of TSS) and KRAB^GFP‐scFv^ expression after DOX treatment (7 days) and DOX washout (4 days).
*Esg1‐*tdTomato expression at single‐cell resolution during DOX washout in control (GFP^scFv^) or induced heterochromatin (KRAB^GFP‐scFv^) cells. Horizontal bars indicate median, each data point a single cell.Histograms of mean DNA methylation levels across the *Esg1* promoter (6 CpG sites) during DOX washout in two biological replicates.CUT&RUN tracks at +DOX, and 4 and 7 days of DOX washout in control GFP^scFv^ or KRAB^GFP‐scFv^ for indicated histone marks. Grey boxes highlight the domain of heterochromatin spreading in +DOX.CUT&RUN qPCR quantification of the relative abundance of each mark at *Esg1* promoter in two or three independent biological replicates, normalised to a positive control region and untransfected cells.
*p53‐*tdTomato expression in single cells during DOX washout in control (GFP^scFv^) or induced heterochromatin (KRAB^GFP‐scFv^) cells. Each data point indicates a cell, and horizontal lines represent the median.Bisulphite pyrosequencing quantification of DNA methylation at the *p53* promoter (4 CpG sites) at indicated time point in two biological replicates.CUT&RUN qPCR quantification of the relative abundance of H3K9me3 and H3K4me3 at *p53* promoter in biological replicates.Heatmap representing relative expression by qRT‐PCR of each indicated gene upon heterochromatin targeting (+DOX) or after DOX washout (D‐wo (7 days)), normalised to the untransfected control in three biological replicates. Statistics are measured between KRAB^GFP‐scFv^ and control (GFP^scFv^) at DOX‐wo time point. Data information: In all panels, asterisks indicate *P‐*values by unpaired *t*‐test; *P < 0.05, **P < 0.01 ***P < 0.001. Error bars ± SD.

Extant paradigms suggest that large domains of heterochromatic H3K9me3, H4K20me3 and DNA methylation are heritable, and self‐propagate via “read‐write” feedback machinery (Hathaway *et al*, 2012; Reinberg & Vales, 2018). To understand this in a developmental context, we next investigated the potential for propagation of induced heterochromatin epialleles in naïve ESC, which faithfully recapitulate *in vivo* epiblast when maintained in 2i/L (Hackett & Surani, 2014). Withdrawal of DOX resulted in a rapid switch off of the iCRUSH epigenetic editing system as determined by quantitative cytometry for GFP, and consistent with dCas9^GCN4^ and KRAB^GFP‐scFv^ being destabilised, therefore fully releasing the inducing signal (Fig 2A). In parallel, we observed a progressive loss of *Esg1* ^tdTomato^ silencing (Fig 2B). Interestingly, 4 days after DOX washout (D‐wo (4 days)), we observed a graded distribution of *Esg1* expression among single cells, indicative of probabilistic reactivation dynamics. By 7 days after release (D‐wo (7 days)), however, all cells reverted to the ON state, reflecting > 500‐fold increase in *Esg1* expression (Fig 2B).

**Figure EV2:**
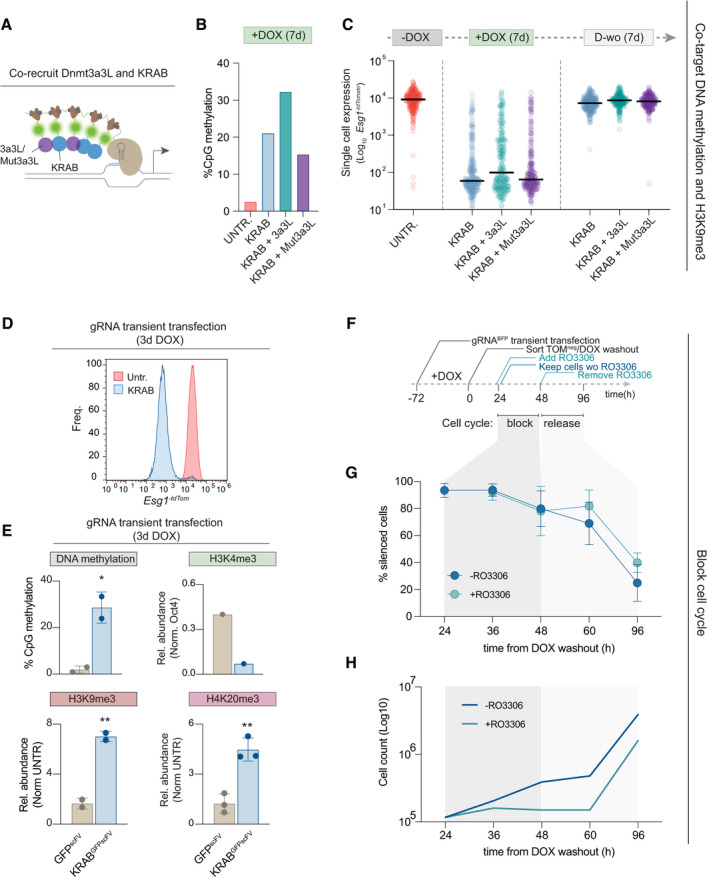
Cell cycle inhibition slows but does not block epigenetic erasure in naïve ESC Schematics of the iCRUSH epigenetic tool used in the experiment. KRAB^GFP‐scFv^ is either recruited alone or in combination with Dnmt3a/Dnmt3L (3a3L^GFP‐scFv^) or the catalytically mutant Mut3a3L^GFP‐scFv^.Bar plot showing the average of the percentage DNA methylation at 6 CpG sites on the *Esg1* promoter assayed by bisulphite pyrosequencing.Violin plots show the single‐cell distribution of *Esg1^‐tdTomato^* expression (log scale) in each condition (−DOX, 7 days +DOX and 7 days DOX washout) upon epigenetic editing with KRAB^GFP‐scFv^ (blue) +/− 3a3L^GFP‐scFv^ (aqua green) or Mut3a3L^GFP‐scFv^ (purple). Each data point indicates a cell, and horizontal lines represent the median.
*Esg ^‐tdTomato^* reporter expression in KRAB^GFP‐scFv^ or untransfected control after gRNA transient transfection and 3 days of DOX induction.Bar plots show percentage of CpG methylation or relative abundance of histone modifications normalised to a positive control region and untransfected control after 3 days of DOX (gRNA transiently transfected) on one, two or three biological replicates. Statistics calculated by unpaired *t*‐test over two or three independent biological replicates (*P < 0.05; **P < 0.01). Error is measured as ± SD.Timeline of the experiment. Briefly, cells are treated for 72 h (3 days) with DOX to induce iCRUSH‐mediated silencing. *tdTomato*‐negative cells are sorted and plated back in culture in duplicate experiments without DOX. After 24 h of DOX washout, one sample is treated with the cell cycle inhibitor RO3306 for further 24 h. After a total of 48 h from DOX washout, the cell cycle is released and cells further analysed at 12 h interval up to 96 h. In parallel, cells are cultured in the absence of the inhibitor.Time course of the percentage of *Esg1^‐tdTomato^*‐silenced cells in the + or ‐RO3306 conditions at 12 h intervals of DOX washout. Error is measured as ± SD between two biological replicates.Line plots indicate log growth of cells in + or – RO3306 conditions.

To determine if transcriptional re‐expression corresponds to loss of programmed epigenetic states, we used bisulphite pyrosequencing and CUT&RUN. Consistent with the reactivation dynamics, we found that DNA methylation is partially maintained at the *Esg1* promoter at the early time point (D‐wo (4 days)) but is almost completely erased by 7 days washout (Fig 2C). However, we found that the high levels of deposited H3K9me3 and H4K20me3 are largely erased by 4 days after DOX withdrawal (Fig 2D and E). Following 7 days release of the epigenetic editing trigger, the *Esg1* chromatin state completely reverts to the initial configuration, including erasure of H3K9me3, H4K20me3 and DNA methylation, and reacquisition of the endogenous H3K4me3 mark (Fig 2D and E). While our system deposits high levels of DNA methylation, we additionally checked whether co‐targeting KRAB^GFP‐scFv^ together with the catalytic domain of *Dnmt3a* and its cofactor *Dnmt3L* (3a3L^GFP‐scFv^) would enhance epigenetic inheritance, since such effects have been reported in cancer and primed cell lines (Fig EV2A) (Amabile *et al*, 2016; Nunez *et al*, 2021). Although we found a slight further increase in DNA methylation by compound recruitment (Fig EV2B), we observed equivalent or faster erasure of epigenetic memory (Fig EV2C). Taken together, our data argue that induction of a robust heterochromatin domain, and consequently extensive epigenetic silencing, is readily reversible from OFF→ON in naïve pluripotent cells.

### Epigenetic inheritance is restricted by naïve ESC

To confirm that failure to propagate robust heterochromatin in ESC is not a phenotype specific to *Esg1*, we generated a second endogenous reporter ESC line by inserting *tdTomato* downstream of the *p53* gene, separated by a T2A self‐cleavable domain (Fig 2F). Targeting KRAB^GFP‐scFv^ to the *p53* ^tdTomato^ promoter recapitulated the same extensive heterochromatin deposition including DNA methylation, H3K9me3 and loss of H3K4me3, and robust (> 100‐fold) single‐cell silencing, as achieved at *Esg1* ^tdTomato^ (Fig 2F–H). Upon 7 days DOX withdrawal, we found that *p53* expression becomes fully reactivated in ESC (Fig 2F–H). This is paralleled by erasure of targeted DNA methylation and H3K9me3, and reacquisition of endogenous H3K4me3, consistent with heterochromatin failing to confer epigenetic memory in naïve ESC.

To examine this across further genomic locations, we programmed heterochromatin to additional endogenous loci, selected to represent different regulatory features (e.g. imprinting control regions, promoters). We imposed strong epigenetic silencing with iCRUSH, yet most loci (*Pten*, *Cdh1*, *Greb1*, *Adamts7*, *Smoc1* and *Jade1*) reverted to their original expression status within 7 days DOX withdrawal (Fig 2I). Nevertheless, we did observe that imprinted genes (*Peg3*, *Mest* and *Plagl1*) are exceptions and, uniquely, maintain memory of *de novo* silencing in naïve ESC (Fig 2I). This suggests that heterochromatin domains at ectopic sites can be epigenetically inherited in a genomic context‐dependent manner, with imprinted loci providing the necessary sequence substrate for propagation. However, in general, we find *de novo* chromatin states at endogenous single‐copy loci are not heritable over extended periods in naïve ESC. This supports a dynamic competition of opposing activities that generally disfavours epigenetic inheritance during the phase of naïve pluripotency, potentially as a safeguard to restrict intergenerational transmission of aberrant epialleles.

**Figure 3:**
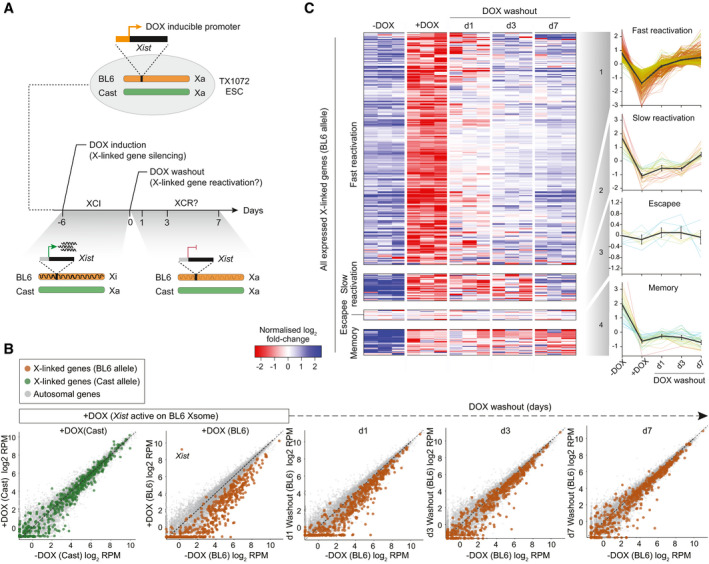
Induced X‐linked gene silencing is reversible in naïve pluripotent cells Schematic representing the workflow of the experiment: wild‐type TX1072 female mESC cells (mixed Cast/BL6), carrying a DOX‐inducible *Xist* on the BL6 allele, are treated with DOX for 6 days to induce *Xist* overexpression driving allele‐specific epigenetic silencing of X‐linked genes in *cis* (XCI). Subsequent DOX washout enables silencing memory to be investigated.Scatterplots showing allele‐specific expression of X‐linked genes on the Cast (green) or BL6 (orange) X‐chromosome following DOX. The dynamics of reactivation of repressed BL6 genes are shown at indicated time points of DOX washout. Grey dots indicate all autosomal genes, which are unaffected.Heatmap of BL6 X‐linked genes in three independent clones at the indicated time points arranged by unsupervised hierarchical clusters: fast reactivation (81%), slow reactivation (8%), escapees (4%) and memory (7%). Line plots indicate the expression trend of each individual gene from the cluster, with black line representing the mean, and error bars 95% CI.

We next asked if this principle in naïve ESC holds for other epigenetic silencing pathways by exploiting a hybrid female ESC line carrying a DOX‐inducible *Xist* allele on the BL6‐derived X‐chromosome (TX1072) (Schulz *et al*, 2014) (Fig 3A). Activation of *Xist* leads to programmed silencing of X‐linked genes in *cis* via recruitment of repressive epigenetic systems, with a principal role for polycomb (Zylicz *et al*, 2019). In differentiated cells, *cis* repression propagates independently, resulting in stable silencing memory (X‐Chromosome inactivation (XCI)), even after withdrawal of *Xist* (Loda & Heard, 2019). However, using transcriptomics, we observed that while strong epigenetic silencing is initially imposed in naïve ESC, withdrawal of DOX led to an almost complete reactivation of X‐linked genes after 3 days (Fig 3B), extending a previous finding based on two marker genes (Wutz & Jaenisch, 2000). Hierarchical clustering revealed the majority of genes (81%) exhibit fast reactivation dynamics (< 3 days) in ESC (Fig 3C). A second cluster (8% of genes) also reactivated but with slower dynamics (< 7 days), and these overlapped with X‐linked loci that reactivate late *in vivo*, for example, *Fmr1b* and *Pnma5* (Borensztein *et al*, 2017). A third cluster (4%) was resistant to initial silencing in naïve cells (ESC escapees), while the final cluster (7% of genes) did exhibit memory of silencing following DOX withdrawal (Fig 3C). This “memory” cluster was enriched for tandem gene families such as the *Rhox*, *Mage* and *Xlr* clusters. Overall, however, these data suggest that the vast bulk of X‐linked genes cannot propagate programmed epigenetic silencing in ESC, which is in contrast to differentiated cells, and supports the principle that naïve pluripotency specifically antagonises epiallele memory.

### CRISPR screen reveals key factors that antagonise epigenetic memory in ESC

To investigate whether the reversal of repressive epialleles in naïve cells is driven by passive dilution during cell divisions, or by active erasure, we transiently transfected iCRUSH to epigenetically silence the *Esg1* reporter for 3 days with DOX. This led to ~100‐fold silencing, deposition of significant levels of DNA methylation, H3K9me3 and H4K20me3, and loss of H3K4me3 (Fig EV2D and E). We then released the epigenetic editing system by DOX washout and concomitantly treated the cells with or without the cell cycle inhibitor RO3306 (Fig EV2F), which blocks cells at the G2/M phase boundary. We observed *Esg1* reactivation is only weakly impaired by cell cycle inhibition (Fig EV2G), spanning at least 60 h (Fig EV2H). Thus, while passive dilution may partially contribute to reversion of epigenetic memory, active mechanisms play a key role in erasing *de novo* epialleles in naïve ESC.

**Figure 4:**
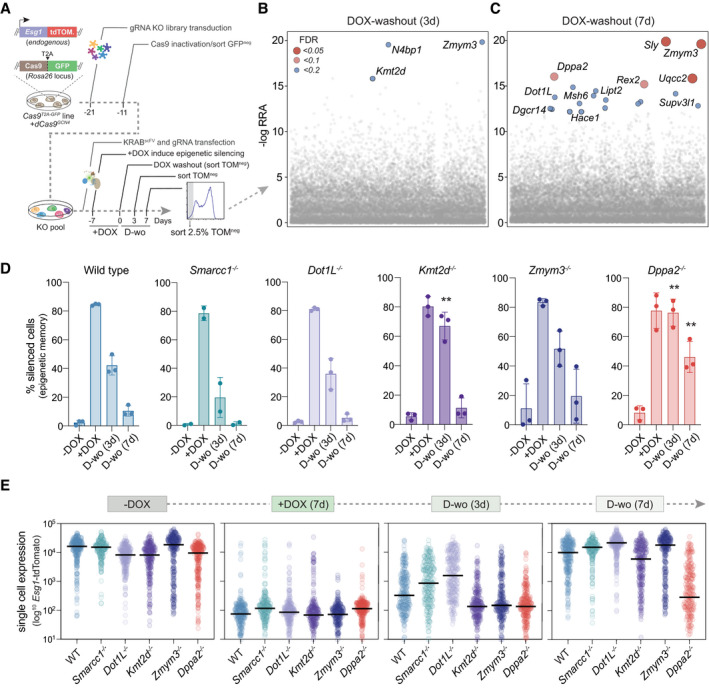
A CRISPR screen identifies key factors that restrict epigenetic inheritance in ESC ASchematic of screen design and workflow: an *Esg1*‐tdTomato cell line carrying constitutive *Cas9* ^T2A‐GFP^ nuclease and DOX‐inducible (off)‐*dCas9^GCN4^* is transduced with a lentiviral gRNA library. The *Cas9* ^T2A‐GFP^ is self‐inactivated via introducing specific gRNAs, and GFP‐negative cells are isolated. Subsequently, gRNA^BFP^ and KRAB^GFP‐ScFv^ are introduced and cells treated with DOX to induce *Esg1* heterochromatin‐mediated silencing (TOM^neg^). TOM^neg^ ESC are flow sorted and re‐cultured in absence of DOX to isolate cells that inherit epigenetic silencing, which are subject to NGS to identify the gene knockout they carry.B, CSignificant hits for factors that permit epigenetic memory when knocked‐out, showing the ‐log relative ranking algorithm (RRA) score at 4 or 7 days of DOX washout. False discovery rate (FDR) is indicated.DHistograms showing the percentage of *Esg1*‐tdTomato*‐*negative cells in WT or knockout ESC lines after programming heterochromatin (+DOX) and during DOX washout. Each data point indicates a biological independent knockout line. Error bars ± SD, asterisks indicate *P‐*values relative to −DOX by unpaired *t*‐test; **P < 0.01.EQuantitative expression of *Esg1‐tdTomato* among single cells in WT or in independent clonal knockout ESC lines. Each data point indicates a cell, and bars represent the median.

We therefore sought to identify the putative factors that actively counteract epigenetic memory in pluripotent phases by designing a genome‐wide loss‐of‐function CRISPR screen (Fig 4A). We introduced a single copy of *Cas9* nuclease tagged with GFP (Cas9^T2A‐GFP^) into *Esg1* ^tdTomato^ ESC that also carry dCas9^GCN4^ in the OFF state, and infected these cells with a pooled lentiviral library of exon‐targeting gRNA covering 19,674 genes (Doench *et al*, 2016). We subsequently induced self‐inactivation of the Cas9^T2A‐GFP^ with a pair of specific gRNAs, which we confirmed by flow sorting cells according to loss of GFP (GFP^neg^) (Fig 4A). This cell population is now composed of a heterogeneous pool of knockout cells, to which we could apply our epigenetic editing system to identify the factors that antagonise epigenetic inheritance.

**Figure EV3:**
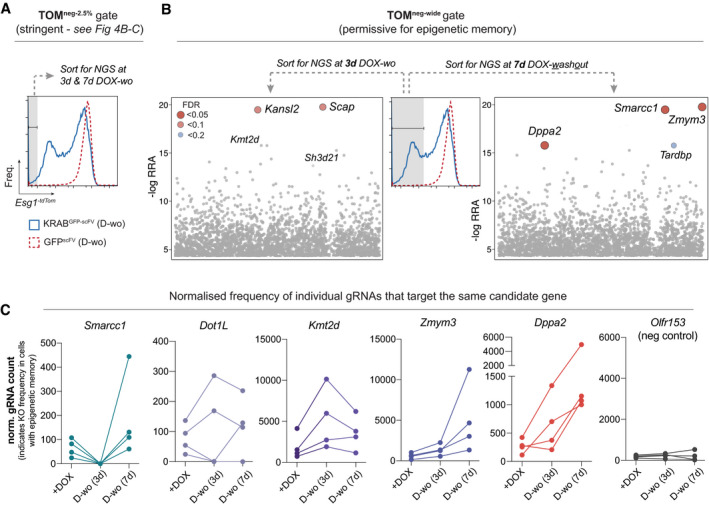
Gating strategy for CRISPR screen and supplementary scatterplots of candidate factors that antagonise epigenetic inheritance Histogram showing the gate used to sort cells that strictly retained epigenetic silencing memory in the CRISPR screen (tdTomato negative) using a stringent gating threshold (bottom 2.5% TOM‐ cells (TOM^2.5%neg^). The results of significant candidate genes that enable this memory when knocked out are detailed in main Fig 4B and C.Middle histogram: A wider gate was used to include all tdTomato‐negative cells (TOM^negwide^) and was designed according to a tdTomato‐positive control sample to capture all cells exhibiting full or partial silencing memory. Scatterplots: show significant hits from the screen displayed by ‐log relative ranking algorithm (RRA) score from the cells captured using the TOM^negwide^ gate at short‐term time point (3 days DOX washout; left) or longer term (7 aysd DOX washout; right). These hits are linked with enabling epigenetic inheritance when abrogated.Line plot for gRNA count from the CRISPR screen after silencing (+DOX) and during memory phase (DOX washout for 3 or 7 days) for the candidate genes indicated. Each line represents a different gRNA from the pool for the same target gene. A concordant enrichment during DOX washout indicates all gRNAs (and therefore independent knockouts of the target gene) promote epigenetic inheritance. Note *Kmt2d* is only enriched at the earlier time point.

To achieve this, we targeted heterochromatin to *Esg1* ^tdTomato^ and isolated cells that subsequently retained silencing memory (TOM^neg^) following release of dCas9^GCN4^::KRAB^GFP‐scFv^ using a gating strategy to distinguish between cells remaining fully silenced (bottom 2.5% (TOM^neg‐2.5%^) (Fig EV3A) and those that retain a degree of repression memory (TOM^neg‐wide^) (Fig EV3B). We then used model‐based analysis of genome‐wide CRISPR‐Cas9 knockout (MAGeCK) to identify the gene knockouts enriched in the TOM^neg^ populations that retained epigenetic memory relative to the complementary TOM^pos^ population over short (3 days) and extended (7 days) timescales (Li *et al*, 2014). As expected, top hits across both gates were associated with roles in transcriptional or translational regulation, and comprised many candidates with established or predicted epigenetic functions. This included the SWI/SNF histone remodeller *Smarcc1* (FDR 0.03), the H3K79 methyltransferase *Dot1L* (FDR 0.16) and the H3K4 histone methyltransferase *Kmt2d* (FDR 0.13), although this latter was enriched only at the shorter time point (Figs 4B and C and EV3B). Additionally, we noted cells that propagated silencing memory also exhibited significant enrichment for knockouts of the X‐linked zinc‐finger protein *Zmym3* (FDR 0.01), the NSL complex subunit *Kansl2* (FDR 0.05) and *Dppa2* (FDR 0.03) (Figs 4B and C, and EV3B and C), which is a pluripotency‐specific gene recently linked with regulating *de novo* DNA methylation and bivalency (Eckersley‐Maslin *et al*, 2020; Gretarsson & Hackett, 2020).

To validate these candidates, we generated independent clonal knockout ESC lines of each. Deletion of these factors did not affect *Esg1* ^tdTomato^ basal activity prior to imposition of epigenetic silencing, and all knockouts also exhibited a comparable extent of programmed silencing as WT after 7 days DOX, implying no changes in initial parameters (Fig 4D). Following DOX washout, *Smarcc1* ^−/−^ and *Dot1L* ^−/−^ reverted to the active state with a similar kinetics to WT, implying false positives. In contrast, *Kmt2d* ^−/−^ cells showed penetrant memory at 4 days of DOX washout, but reverted to an ON state after 7 days, suggesting absence of *Kmt2d* impacts the rate of memory erasure, potentially by affecting re‐deposition of H3K4me3 (Fig 4D and E). Interestingly, while some *Zmym3* ^−/−^ lines exhibited memory, and independent gRNAs were concordant (Fig EV3C), there was high heterogeneity between independent knockout clones, indicating a complex regulatory response that we did not follow further. However, all *Dppa2* ^−/−^ lines fully maintained epigenetic memory after 3d DOX withdrawal, with the majority of cells remaining in the OFF state after 7 days (Figs 4D and E, and EV3C). This suggests that abrogating *Dppa2* changes the balance of activates in ESC to generate an environment that is permissive for epigenetic inheritance.

### Epigenetic inheritance is permitted by deletion of DPPA2

**Figure EV4:**
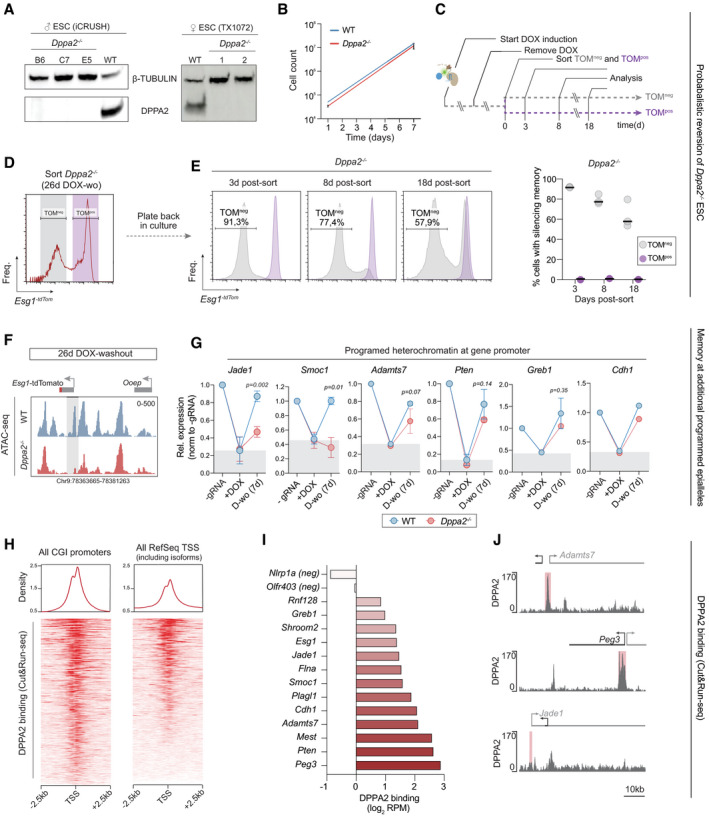
Epigenetic inheritance is probabilistic upon Dppa2 knockout Western blot comparing DPPA2 protein expression in WT and *Dppa2* knockout individual ESC clonal lines. β‐TUBULIN is used as loading control.Line plot indicates exponential growth of wild‐type or *Dppa2* ^−/−^ cells.Timeline of the experiment in D and E. After induction for 7 days, tdTomato‐negative (TOM^neg^) and ‐positive (TOM^pos^) fractions of cells are sorted following 26 days of DOX washout in *Dppa2* ^−/−^ and put back in culture separately. Flow cytometry analysis is performed after 3, 8 or 18 days after sorting.
*Esg1^‐tdTomato^* expression in *Dppa2* ^−/−^ after 26 days of DOX washout. Grey and purple boxes indicate the gates used to sort TOM^neg^ and TOM^pos^ fraction of cells respectively.Left, distributions of the TOM^neg^ and TOM^pos^ cell population over time, showing a probabilistic reversion to active status that is cumulative, indicating a stochastic memory function. Right, percentage of silenced cells of TOM^neg^ and TOM^pos^ in three independent *Dppa2* ^−/−^ clones, after 3, 8 or 18 days post‐sort.ATAC‐seq tracks showing chromatin accessibility at *Esg1‐tdTomato* after 26 days of DOX washout in wild‐type or *Dppa2* ^−/−^ lines. Grey box highlights *Esg1* promoter.Line plots show gene repression (+DOX) and memory (D‐wo) of the indicated targets relative to the housekeeping gene *Rplp0* and normalised to ‐gRNA control. Grey boxes represent the level of silencing achieved after +DOX treatment in the *wild‐type* condition. All non‐imprinted targets exhibit increased memory in *Dppa2* knockout, albeit insignificant, which likely reflects stochastic memory function at the single‐cell level (i.e. > 50% of the cells still revert in *Dppa2* ^−/−^, thereby blunting the effect in population measurements). Error bars are ± SD out of two or three biological replicates. Statistics is measured by one‐tail unpaired *t*‐test between WT and *Dppa2* knockout conditions.Distribution of DPPA2 binding (CUT&RUN‐seq) over TSS ± 2.5 kb considering CG dense promoters or all promoters.Histograms showing DPPA2 occupancy (CUT&RUN‐seq) at the targets reported in (G) and Fig 5H.Chromosome tracks of representative examples from (I).

**Figure 5:**
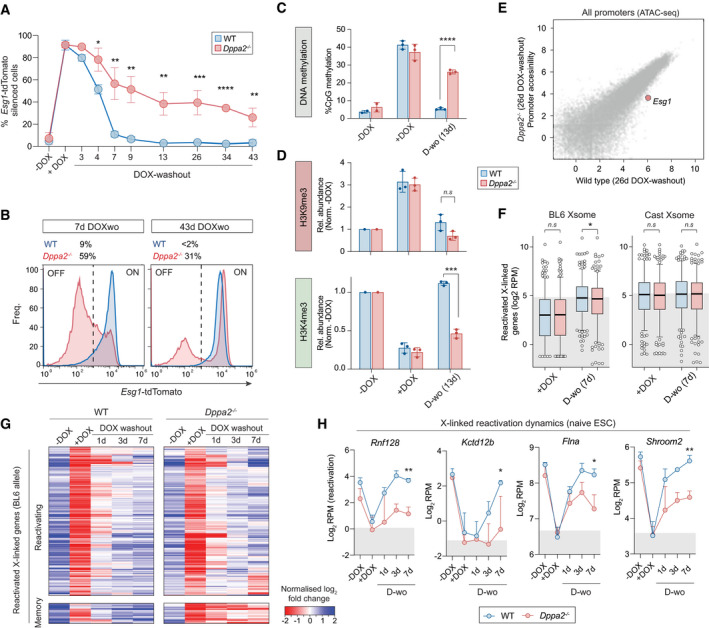
Epigenetic inheritance is unlocked by deletion of *Dppa2* Time course showing percentage cells that propagate heritable epigenetic silencing of *Esg1*‐tdTomato in wild‐type or *Dppa2* ^−/−^ cells. Each data point represents average of three independent clonal lines.Distribution of quantitative *Esg1*‐tdTomato expression in *Dppa2* ^−/−^ (red) or wild type (blue) after 7 or 43 days DOX washout showing bimodal epigenetic memory only upon *Dppa2* abrogation, in one representative example. Numbers indicate percentage of *Esg1‐*tdTomato‐negative cells.Bisulphite pyrosequencing quantification of DNA methylation at *Esg1* promoter in WT (blue) or *Dppa2* ^−/−^ (red) ESC, each assayed in two or three independent lines.CUT&RUN‐qPCR quantification of H3K9me3 and H3K4me3 relative to a positive control region and to the −DOX control in one (−DOX) or three independent clonal lines.Scatterplot of genome accessibility across all promoters after 26 days DOX withdrawal, showing a specific and persistent memory at *Esg1* in *Dppa2* ^−/−^ cells.Boxplot showing expression (log_2_ RPM) of all expressed X‐linked genes on either the BL6 or control cast allele in WT and *Dppa2* ^−/−^ at +DOX or 7 days after DOX washout (7 days D‐wo) time points. Bars indicate median from independent clonal lines of *Dppa2* ^−/−^ or WT. Boxes indicate quartiles, and whiskers represent the 5^th^ to 95^th^ percentiles.Heatmap indicating expression dynamics of X‐linked genes that reactivate in WT. Shown are average profiles from independent three WT and two *Dppa2* ^−/−^ clonal lines at the indicated time points, which form two unsupervised hierarchical clusters: genes that reactivate in both WT and *Dppa2* ^−/−^ (upper) and genes which maintain memory only in *Dppa2* ^−/−^ (lower).Expression dynamics (log_2_ RPM) of four representative genes from the memory cluster. Statistics calculated between two or three in independent clonal lines in *Dppa2* ^−/−^ or WT, respectively, at 7 days DOX washout. Data information: In all panels, asterisks indicate *P‐*values by unpaired *t*‐test; *P < 0.05, **P < 0.01 ***P < 0.001. In panels (A, C, D and H), error bars are ± SD.

To examine the role of *Dppa2* further, we traced the single‐cell dynamics of transcriptional memory in multiple‐knockout ESC lines (Fig EV4A). While WT cells rapidly lose silencing memory after 7 days DOX washout, the majority of *Dppa2* ^−/−^ cells remain fully silenced at this stage (Fig 5A). Importantly, inheritance of this silenced state in the absence of *Dppa2* is subsequently maintained across a consistent fraction of cells for at least 43 days after DOX withdrawal (> 100 cell replications), with the population therefore exhibiting a bimodal distribution (Fig 5B). Importantly, population doubling time was similar between wild‐type and knockout cells (Fig EV4B). This implies that abrogation of *Dppa2* facilitates heritability of ectopic silencing in a probabilistic manner, potentially by shifting the odds against reversion, and thus promoting steady‐state inheritance. Notably, flow sorting TOM^neg^ and TOM^pos^ fractions after 26 days of DOX washout revealed that, while the TOM^pos^ remained positive, the TOM^neg^ reacquired a bimodal distribution, supporting a stochastic memory function (Fig EV4C–E).

We next investigated transmission of programmed chromatin states in *Dppa2* ^−/−^ cells. After DOX induction of iCRUSH, we observed that DNA methylation and H3K9me3 are deposited comparably in both WT and *Dppa2* ^−/−^ cells, and endogenous H3K4me3 is equivalently erased (Fig 5C and D). Upon release of dCas9^GCN4^::KRAB^GFP‐scFv^ (DOX washout), however, while WT cells underwent a complete recovery of the epigenetic landscape, *Dppa2* ^−/−^ exhibited significant inheritance of DNA methylation (Fig 5C), and also propagated the H3K4me3‐depleted state at *Esg1* (Fig 5D). Moreover, ATAC‐seq revealed that induced chromatin inaccessibility status was transmitted mitotically in *Dppa2* ^−/−^ ESC but not WT (Figs 5E and EV4F).

To determine the generality of epiallele propagation in *Dppa2* ^−/−^ ESC, we targeted heterochromatic silencing to additional loci, which we previously showed do not exhibit memory in wild‐type ESC (Fig 2I). Here, a trend of memory was propagated at *Pten*, *Cdh1*, *Greb1 and Adamts7* in the absence of *Dppa2* (Fig EV4G). While these did not reach significance, this could potentially reflect an incompletely penetrant memory (bimodality) at the single‐cell level similar to *Esg1*, which we cannot resolve at the population level by qRT‐PCR. Importantly, however, we did observe significant inheritance of an induced repressed state at *Jade1* (aka *Phf17*) and *Smoc1*, specifically in *Dppa2* ^−/−^ ESC.

To expand this analysis to a larger unbiased scale, we generated *Dppa2* knockout ESC in the inducible *Xist* background (TX1072) (Fig EV4A) and assayed the transcriptome following DOX withdrawal, which releases *Xist*‐mediated epigenetic silencing. Analysis of all X‐linked loci that are reactivated in wild‐type ESC revealed *Dppa2* ^−/−^ cells exhibited a significant (*P = 0.041*), albeit modest, block in gene re‐expression following release of *Xist* (Fig 5F). Hierarchical clustering deconvolved two broad groupings. The first gene cluster (92.9%) reactivated in *Dppa2* ^−/−^ ESC comparably with wild‐type kinetics. In contrast, the second cluster (7.1%) identified a broad set of genes that exhibit epigenetic memory of prior silencing specifically in the absence of *Dppa2*, including *Flna*, *Shroom2*, *Kctd12b* and *Rnf128* (Fig 5G and H). Of note, epigenetic silencing induced by *Xist* is preferentially linked with polycomb pathways (H3K27me3 and H2AK119ub) and histone deacetylation (Zylicz *et al*, 2019), whereas targeting with iCRUSH programmes repression with H3K9me3, H4K20me2 and DNA methylation epialleles. This suggests absence of DPPA2 unlocks the potential for propagating at least two distinct modes of heterochromatin‐based silencing in naïve ESC.

Taken together these data suggest that once an aberrant heterochromatic state occurs, pluripotent cells rely, at least partly, on DPPA2 to re‐establish the original epigenetic configuration. Indeed, using CUT&RUN‐seq to chart DPPA2 occupancy in ESC, we observed that DPPA2 binds strongly to all CpG‐dense gene promoters (Fig EV4H), and > 70% of all transcriptional start sites (TSS), including all responsive targets tested above (Fig EV4I and J). This implies DPPA2 acts as an epigenome “surveyor” in naïve pluripotent cells by sampling most promoters and promoting probabilistic reversion of epimutations. While parallel erasure mechanisms must also operate, since epialleles at many genes revert independent of *Dppa2*, DPPA2 activity underlies the capacity of naïve ESC to reset aberrant epigenetic states across a significant cohort of sensitive loci.

### Aberrant epialleles can be propagated upon exit from pluripotency

**Figure 6:**
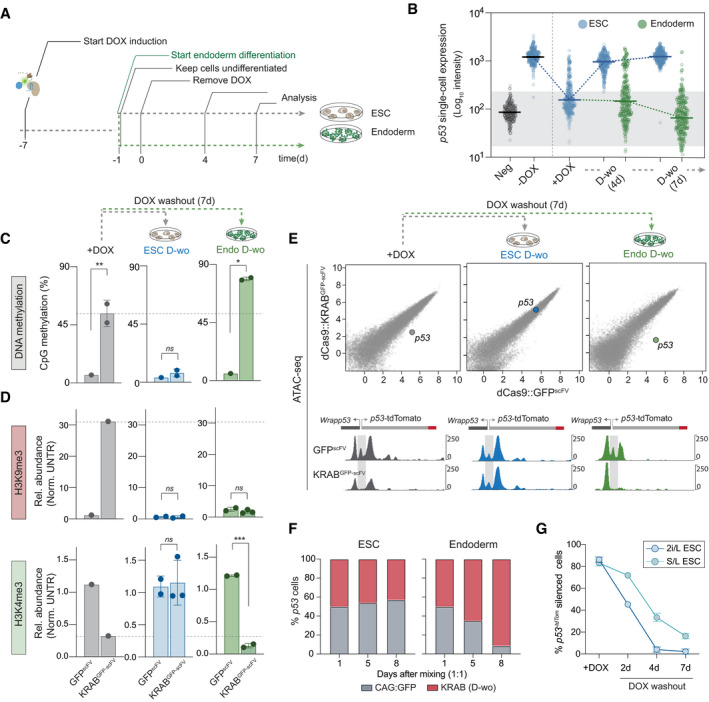
Aberrant epialleles can be propagated in wild‐type cells upon exit from pluripotency Schematic of experimental timeline: heterochromatin epialleles are induced at *p53*‐tdTomato in ESC followed by endoderm differentiation (day −1), with DOX removed after 24 h (day 0). In parallel, cells are maintained as naïve ESC. Chromatin and expression analysis to record memory in endoderm and ESC is performed at 4 and 7 days of DOX washout.Single‐cell expression of *p53‐*tdTomato during DOX washout in endoderm or naïve ESC, following induction of heterochromatic silencing. Each data point indicates a cell, and horizontal lines represent the median.Bisulphite pyrosequencing quantification of DNA methylation at the *p53* promoter in ESC or endoderm following DOX withdrawal in two or three biological replicates for KRAB^GFP‐scFv^. Dashed line indicates levels of DNA methylation after targeting KRAB^GFP‐scFv^ (+DOX) for 7 days.CUT&RUN qPCR quantification of the relative memory of induced H3K9me3 and H3K4me3 at *p53* promoter in independent biological replicates of endoderm or ESC.ATAC‐seq scatterplots showing genome accessibility at all promoters comparing GFP^scFv^ and KRAB^GFP‐scFv^ in +DOX or DOX washout conditions in ESC or endoderm cells. Shown below are relevant genome tracks of the *p53* promoter (highlighted in grey).Cell growth competition assay following 1:1 mixing of cells bearing control GFP or KRAB^GFP‐scFv^ epigenetic silencing of *p53*, in ESC or endoderm.Line plot showing the percentage of *p53‐*tdTomato epigenetically silenced cells in 2i/L and serum/Lif (S/L) culturing conditions at +DOX and 2, 4 or 7 days of DOX washout. Error bars are ± SD measured over two biological replicates. Data information: In all panels, asterisks indicate *P‐*values by unpaired *t*‐test; *P < 0.05, **P < 0.01 ***P < 0.001. Error bars ± SD between two and three biological replicates.

*Dppa2* is only expressed during pluripotent phases suggesting epialleles acquired during or after this may confer heritable mitotic memory through subsequent lineage commitment when DPPA2 is absent. To investigate this, we differentiated wild‐type ESC towards definitive endoderm as an *in vitro* model of development (Fig 6A). Because *Esg1* is repressed during differentiation as part of the normal developmental programme, we initially focused on *p53*, wherein heterochromatin and transcriptional silencing are also robustly erased in ESC (Fig 2F–H).

**Figure EV5:**
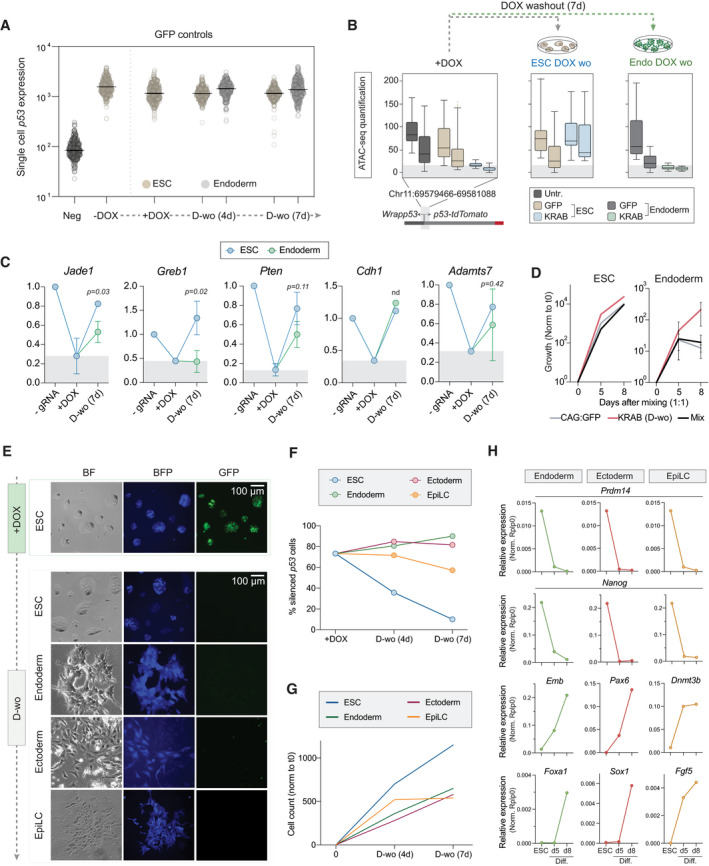
Epigenetic memory is maintained over differentiation programmes representative of the three germ layers Expression of the *p53^‐tdTomato^* reporter at the single‐cell level in ESC or endoderm‐differentiated cells in the DOX condition indicated, using only the GFP control, confirming no effect (compared to main Fig 6B). Black horizontal bars represent the median of fluorescence intensity in the population of cells (single dots).Box plot quantification of the ATAC‐seq accessibility at the *p53* promoter region in two biological replicates side by side. Boxes represent upper and lower quartile out of 800 values, horizontal bar is the median. Whiskers are the 5^th^ to 95^th^ percentiles.Line plots show expression of the indicated targets relative to the housekeeping gene *Rplp0* and normalised to ‐gRNA control for each time point in ESC or endoderm cells. They demonstrate most loci do not exhibit memory of epigenetic silencing in ESC or endoderm, but some (e.g. *Jade1* and *Greb1*) show selective epigenetic inheritance in endoderm. Grey boxes represent the level of repression achieved after DOX treatment in the wild‐type condition. Error bars are standard deviation out of two or three biological replicates. Statistics is measured by unpaired *t*‐test between WT and *knockout* conditions.Line plots indicate exponential growth of CAG:GFP, KRAB^‐GFP‐scFv^ (D‐wo) or the mixed population in one ESC or three endoderm cell lines used for the experiment in Fig 6F. Error bars are ± SD.Representative microscopy images of bright field and BFP or GFP fluorescence of ESC, endoderm, ectoderm or EpiLC‐differentiated cells upon DOX induction (7 days) or DOX washout (7 days).Time course showing epigenetic memory of *p53‐tdTomato* silencing during DOX washout in ESC, endoderm, ectoderm and EpiLC.Line plots show ratio of cell count normalised to time point 0 in ESC, endoderm, ectoderm or EpiLC‐differentiated cells.Time course showing relative expression of naïve pluripotency (*Prdm14* and *Nanog*) or differentiation markers (*Emb* and *Foxa1* for endoderm; *Pax6* and *Sox1* for Ectoderm; *Dnmt3b* and *Fgf5* for EpiLC) compared to the housekeeping gene *Rplp0* in ESC and during differentiation programmes after release of the silencing trigger on *p53‐tdTomato* reporter.

In contrast to ESC, upon differentiation to definitive endoderm, we observed a highly penetrant memory of *p53* silencing among single cells (Fig 6B), whereas no memory effects were observed upon control targeting with GFP^scFv^ (Fig EV5A). Analysis of chromatin revealed inheritance of targeted DNA methylation (> 85%) at *p53* specifically in differentiating endoderm cells (Fig 6C), while there is also heritable memory of the H3K4me3 depletion (Fig 6D). Interestingly, deposited H3K9me3 is erased in endoderm (Fig 6D), implying it does not self‐reinforce or drive silencing in this context. To investigate this epigenetic memory further, we used ATAC‐seq and observed highly significant loss of accessibility specifically at targeted *p53* upon *de novo* heterochromatin formation (Figs 6E and EV5B). Following 7 days DOX withdrawal, this inaccessible chromatin state exhibited robust memory during endoderm differentiation. In contrast, chromatin accessibility is restored in ESC upon DOX withdrawal (Fig 6E). These data imply that differentiated cells, but not naïve pluripotent ESC, are competent for epigenetic inheritance of ectopic heterochromatin.

To extend this we next targeted heterochromatin to five additional endogenous loci and tracked their memory in *wild‐type* endoderm. We found that *Jade1* and *Greb1* exhibit robust inheritance (*P* < 0.05) of a prior silenced state specifically in endoderm (Fig EV5C), whereas *Cdh1*, *Adamts7* and *Pten* reinstate their original activity, implying a degree of context dependency. Notably of all targets, *p53* exhibited the most striking propagation of silencing, which we reasoned may reflect a confluence of epigenetic memory and a selective advantage, given the role of *p53* in restricting proliferation. Indeed, by mixing equal (1:1) proportions of silenced *p53* cells with untargeted controls and withdrawing DOX, we found epigenetically repressed cells became dominant in the endoderm population, comprising > 95% by d8, but not in naïve ESC, where memory of prior silencing is rapidly erased (Fig 6F). Moreover, endoderm cells with prior *p53* silencing replicate faster and with greater viability (Fig EV5D). Taken together, these data suggest that the potential for epiallele inheritance of *de novo* heterochromatin is influenced by multiple cell type‐ and genomic context‐dependent factors. In the case of *p53*, augmenting weak‐acting heterochromatin inheritance in differentiated cells with a favourable advantage may tip the balance of dynamic forces to enable robust propagation of ectopic chromatin states within the population.

To investigate whether the difference in epiallele propagation between naïve and committed cells is functionally linked with global DNA hypomethylation in naïve ESC (Leitch *et al*, 2013), we switched ESC to serum/LIF culture (S/L). S/L maintains ESC populations as functionally naïve (they contribute to blastocyst chimeras), but promotes a more developmentally advanced epigenetic state, including DNA hypermethylation, thereby enabling us to parse the influence of global DNA methylation status on memory. We observed that programmed heterochromatic silencing at *p53* is readily erased in S/L ESC, albeit with modestly slower dynamics than 2i/L ESC (Fig 6G). In contrast, differentiated cells maintain silencing memory (Fig 6B). The delayed dynamics in S/L relative to 2i/L could indicate a contributory, but non‐essential role of global DNA hypomethylation for erasure, or alternatively may reflect the metastable status of ESC in S/L, with some subpopulations not in “naïve” status. In any case, these data argue that global hypomethylation *per se* is not requisite, and that additional properties of naïve cells underlie their unique capacity to reset acquired heterochromatin states (including *Dppa2* activity).

### Epigenetic inheritance during mammalian development *in vivo*

To more closely model the developmental process that occurs *in vivo* when pluripotent cells differentiate into all lineages, we differentiated ESC towards fates representative of multiple germ layers: ectoderm, endoderm and epiblast‐like cells (EpiLC) (as a model of primed pluripotency). Upon release of the iCRUSH heterochromatic trigger (Fig EV5E), we observed maintenance of *p53* silencing in all three differentiation programmes (Fig EV5F). Notably this included primed pluripotent EpiLC, emphasising the preferential capacity of *naïve* pluripotency to reset epialleles. All differentiating cells replicated with comparable kinetics (Fig EV5G) and activated master lineage regulators, while repressing naïve markers, indicating successful differentiation (Fig EV5H). These data suggest that epigenetic aberrations acquired during the pluripotency window can potentially be inherited in all tissues.

**Figure 7:**
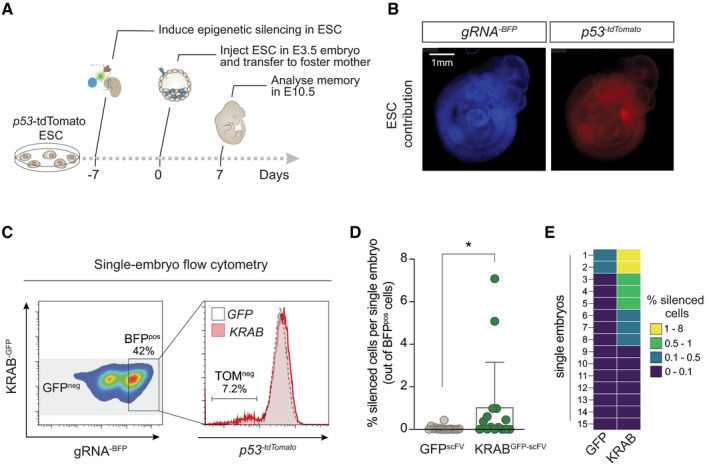
Induced epialleles exhibit epigenetic inheritance during *in vivo* development Schematic of experimental strategy to trace induced epialleles during *in vivo* development.Epifluorescence images showing good contribution of ESC (marked in BFP expression) to E10.5 chimeric embryos.Left: flow cytometry density plot of gRNA^BFP^ and KRAB^GFP‐scFv^ expression of a E10.5 embryo. Shown right is the distribution of *p53* expression demonstrating memory of induced *p53* silencing (KRAB) relative to control (GFP), in cells gated for BFP positive and GFP negative.Percentage of *p53*‐silenced cells in BFP^pos^/GFP^neg^ population. Each dot represents a single embryo from two independent experiments (*n* = 15 per condition). Asterisks indicate *P‐*values by unpaired *t*‐test; *P < 0.05. Error bars ± SD.Heatmap showing the percentage of cells in each embryo (*n* = 15 per condition) that retained epigenetic silencing memory of *p53*.

To determine whether perturbed epigenetic states acquired at loci such as *p53* can self‐propagate during *in vivo* development, potentially affecting disease risk, we tested epigenetic inheritance during embryogenesis. We introduced *p53* epigenetically‐silenced ESC (KRAB^GFP‐scFv^) into E3.5 blastocysts and traced the memory during post‐implantation development (−DOX), as compared to a control (GFP^scFv^) (Fig 7A). By epifluorescence microscopy, we observed a strong contribution of ESC to all tissues of the E10.5 embryo (Fig 7B), and a consistent fraction of the cells carried BFP expression, as analysed by flow cytometry (Fig 7C). Analysis of tdTomato within the BFP^pos^ cells revealed that *p53* is fully activated in controls, as expected (Fig 7C). In contrast, embryos with prior *p53* epigenetic silencing had a significant tendency (*P* = 0.03) to propagate memory of this through development (Fig 7D). Indeed, up to 7% of foetal cells inherited epigenetic silencing memory (Fig 7D and E). Given the central role of *p53* as a tumour suppressor, this has the potential to have a major impact on disease susceptibility.

Overall, these data suggest that an epiallele acquired during or after naïve pluripotent phases can be inherited through subsequent development. Importantly, this effect is highly context dependent and relies on supporting activities or influences that reinforce or promote propagation, either directly or indirectly.

## Discussion

Here, we used a precision epigenetic editing strategy (iCRUSH) to define the transcriptional function and memory of heterochromatin epialleles at endogenous loci. Our method of compound recruitment of multiple “effector” modules using dCas9^GCN4^ facilitated programming of major (> 10 kb) heterochromatin domains, sufficient to drive robust epigenetic silencing. These *de novo* domains comprised H3K9me3, H4K20me3 and DNA methylation, and concomitant loss of H3K4me3, with modification levels comparable or greater than endogenous heterochromatic regions, which are thought to self‐propagate via “read‐write” reinforcement (Reinberg & Vales, 2018).

Nevertheless, we found that naïve pluripotent cells act as a fundamental roadblock to inheritance of heterochromatin domains occurring outside of normal genomic contexts, even when providing a selective advantage such as silencing *p53*. This supports the concept of an epigenetic “*tabula rasa*” during early mammalian development that acts to prevent intergenerational transmission of inherited or acquired chromatin epialleles. An exception to this principle is imprinted regions, wherein programmed chromatin was stably maintained, highlighting the role of underlying DNA sequence context for epigenetic memory. This contextual influence is further exemplified by the effects of cell identity, with our data revealing the potential for epigenetic inheritance in mammals during normal *in vivo* development. Thus, we propose a unique and defining feature of naïve pluripotency is to reset aberrant chromatin states at endogenous loci to establish a pristine epigenome for development. Indeed, the functional properties of pluripotency *per se* are relatively unaffected by impairing global DNA demethylation (McLaughlin *et al*, 2019), and thus purging otherwise heritable epialleles could be a key operative function of epigenome reorganisation during pluripotent phases (Festuccia *et al*, 2016).

To decipher the underlying mechanisms that restrict epigenetic inheritance in naïve pluripotent cells, we designed a genome‐wide CRISPR screening strategy. We found that loss of *Kmt2d* enables prolonged memory of epigenetic silencing in naïve ESC, presumably because of reduced H3K4me3 re‐deposition, yet the original epigenetic state is eventually restored, suggesting an important but non‐critical role of *Kmt2d* in opposing heritable silencing. In contrast, we show that deletion of *Dppa2* enables robust long‐term epigenetic inheritance of programmed epigenetic silencing in naïve cells. Interestingly, this was a probabilistic effect, with most cells potentiating memory, but with a fraction delaminating to reactivate expression over time. This suggests that removing *Dppa2* shifts the balance of opposing factors to favour propagation of epigenetic silencing without fully saturating the odds against reversion.

Importantly, in *Dppa2* mutants, the majority of loci remain in their erstwhile epigenetic configuration prior to acquiring a forced epimutation. This argues that loss of *Dppa2* sensitises some loci to stably inherit any stochastic or programmed epigenetic changes, and implies DPPA2 acts as an epigenome surveyor to counteract epigenetic inheritance during pluripotent phases. This extends recent observations which showed a subset of developmental loci and LINE1 directly acquire silencing in *Dppa2* mutants (Gretarsson & Hackett, 2020), by revealing that loss of *Dppa2* renders a further fraction of the genome predisposed to inherit prospective epigenetic perturbations, potentially induced by external exposures. Mechanistically, DPPA2 is thought to target H3K4me3 through interactions with the COMPASS complex (Eckersley‐Maslin *et al*, 2020), which emphasises that the molecular pathways that impair heterochromatin inheritance in ESC could converge on promoting antagonistic H3K4me3. Indeed, we found that, in the absence of DPPA2, memory is favoured at non‐canonically imprinted H3K4me3 genes (*Jade1* and *Smoc1*) (Hanna *et al*, 2019). DPPA2 is also a putative mitotic bookmarking factor, which may be functionally relevant for restricting aberrant epigenetic memory through cell division (Djeghloul *et al*, 2020). Nevertheless, it is important to note that additional or redundant factors are at play, since we observed epigenetic reversion of many loci in *Dppa2* mutants, such as *p53* and X‐linked genes. This suggests there is a broad network of mechanisms that precludes epigenetic inheritance specifically in naïve cells, with DPPA2 playing a key role within these regulatory systems.

In contrast to naïve cells, heritable epigenetic silencing has been reported in differentiated cells (Amabile *et al*, 2016; Bintu *et al*, 2016; Nunez *et al*, 2021). This has, however, typically been in cancer‐derived cell lines and the potential for mitotic propagation of *de novo* epigenetic states in a normal developmental context is relatively unexplored. We reasoned that if an epimutation occurred during or after early pluripotent phases *in vivo*, it could heritably influence subsequent cellular/organismal phenotype, via clonal inheritance in neighbouring cells. Indeed, we found that while epigenetic silencing at some loci is reset, others demonstrate robust mitotic transmission of heterochromatin‐mediated silencing, including *p53*. This appears to reflect the confluence of weak‐acting probabilistic inheritance and a selective advantage conferred by stable *p53* repression. Such a phenomenon of ‘Darwinian’ epigenetic inheritance was recently shown in yeast (Catania *et al*, 2020; Torres‐Garcia *et al*, 2020). Importantly, we demonstrate this effect *in vivo* in mice, with up to 7% of cells within a whole embryo heritably maintaining the legacy of prior *p53* silencing. This is relevant as even a small fraction of organismal cells correspond to a large absolute number (order of 10 billion in adult mouse) that carry epigenetic silencing, and such constitutional epimutations of tumour suppressors have been linked to cancer risk (Hitchins, 2015; Saunderson *et al*, 2017). Moreover, the principle of probabilistic inheritance of epigenetic silencing *in vivo* shown here could have implications for other aspects of health and disease linked with early life environmental exposures that can promote epigenome changes.

In summary, we find the window of naïve pluripotency robustly counteracts induced epigenetic memory, in part through DPPA2 activity, implying an intrinsic role of naïve status could be to erase epimutations during early mammalian development. Upon differentiation, however, when *Dppa2* is downregulated, we find acquired chromatin states can self‐propagate through development, particularly when providing a selective advantage. This highlights a previously unappreciated but crucial developmental function of naïve pluripotency, and contributes to understanding the complex inputs that feed into epiallele propagation in mammals.

## Materials and Methods

### Routine cell culture

Naïve murine embryonic stem cells (mESC) were derived freshly (mixed 129/B6, XY), or obtained from (Hackett *et al*, 2018), and were routinely cultured on gelatin‐coated plates in t2i/L media: NDiff (N2B27) (Takara #y40002) supplemented with titrated 2i (0.2 μM PD0325901 and 3 μM CHIR 99021), 1,000 U/ml leukaemia inhibitory factor (LIF), 1% FBS, 1% penicillin streptavidin and maintained in humidified atmosphere at 37°C and 5% CO_2_. Cells were passaged every 2–3 days by dissociation with TrypLE and medium was changed daily. Mycoplasma contamination checks were performed routinely by ultrasensitive qPCR assay (Eurofins).

For X‐chromosome reactivation experiments, TX1072 cells (mixed Cast/B6, XX) were a gift from Edith Heard, and were cultured as described before in 2i+Lif media: DMEM (Sigma), 15% FBS (Gibco), 1% penicillin streptavidin, 0.1 mM β‐mercaptoethanol, 1,000 U/ml (LIF), CHIR99021 (3 μM) and PD0325901 (1 μM).

### DNA transfection

DNA transfection was accomplished with Lipofectamine 3000 (Thermo Fisher Scientific #L3000001) unless otherwise stated. ES cells were seeded at least 24 h in advance to be ~50% confluent on the day of transfection. Appropriate amounts of DNA were calculated according to manufacturer’s instructions. Media were changed after 6 h, and replaced with antibiotic selection containing medium where appropriate.

### Flow cytometry

For fluorescence‐activated cell sorting (FACS) or flow analysis, cells were gently dissociated in cell suspension by TrypLE, resuspended in PBS plus FBS 1% (FACS media) and filtered (BD, cup‐Filcons #340632). A FACS Aria III (Becton Dickinson) or Attune NxT Flow Cytometer (Thermo Fisher Scientific) were used for sorting or analysis respectively. Data analysis was performed with FlowJo v10.5.3 (Tree Star, Inc.).

### Epigenetic editing tool constructs

Epigenetic editing tools comprising dCas9^GCN4^, KRAB^GFP‐scFv^, 3a3L^GFP‐scFv^, Mut3a3L^GFP‐scFv^ and GFP^scFv^ were cloned into PiggyBac recipient plasmids, properly linearised with restriction enzymes, by homology arm recombination using In‐fusion HD‐Cloning (Takara #639650) according to manufacturer’s instructions (Fig EV1A). For the pPB_TRE3G::dCas9^GCN4^_*EF1a::*TetOn‐Hygro, the *Streptococcus pyogenes* dCas9^GCN4^ was PCR amplified from the PlatTET‐gRNA2 plasmid (Morita *et al*, 2016) (Addgene #82559), and cloned together with a d2 destabilisation domain under control of the TRE3G promoter in a PiggyBac backbone vector also containing the TET‐ON3G transactivator and the hygromycin resistance gene separated by an IRES sequence and under control of the CAG promoter. For the effector plasmids (pPB_TRE3G::ScFv‐GFP‐KRAB_EF1a::Neo and pPB_TRE3G::ScFv‐GFP‐3a3L_EF1a::Neo), the GCN4‐specific scFv domain and the sfGFP gene were amplified from PlatTET‐gRNA2 plasmid (Addgene #82559) and fused in frame with the human ZNF10 KRAB domain (amplified from the pAAVS1‐NDi‐CRISPRi (Addgene #73498)) or the catalytic domain (CD) of mouse Dnmt3a and the C‐terminal part of mouse Dnmt3L (3a3L) (amplified from pET28‐Dnmt3a3L‐sc27 (Addgene #71827)) and cloned in PiggyBac plasmids under control of the TRE3G promoter. The effector is also destabilised with a d2 domain and the vector also carries constitutive expression of the Neomycin resistance gene. The control effector GFP^scFv^ was cloned as described above but without any epigenetic domain. Finally, abolishment of the cytosine methyltransferase catalytic activity in Dnmt3a CD (Mut3a3L) was achieved by a single replacement of a cysteine at position 296 by a serine (Hsieh, 1999).

Similarly, the U6::gRNA_EF1a::BFP‐Puro, carrying an enhanced gRNA scaffold, was amplified from Addgene plasmid #60955 and cloned into a PiggyBac recipient plasmid (pPB_U6::gRNA_EF1a::BFP‐Puro).

To design all sgRNA for targeting the epigenetic editing system, the GPP web portal (Broad Institute) was used. Reverse complement gRNA sequences (Table EV1) with appropriate overhangs were annealed at 10 μM final concentration with 10 mM Tris, pH 7.5–8.0, 60 mM NaCl, 1 mM EDTA, by heating at 95°C for 3 min and cooling down at RT for > 30 min. Annealed sgRNA was ligated with T4‐DNA ligase (NEB #M0202S) for 1 h at 37°C into pPB_U6::gRNA_EF1a::BFP‐Puro digested with BlpI (NEB #R0585S) and BstXI (NEB #R0113S). All ligated assembled plasmids were amplified by bacteria transformation and purified by endotoxin‐free midi‐preparations (ZymoResearch #D4200). Correct assembly and sequences were confirmed by Sanger sequencing (Genewiz).

### Generation of reporter cell lines

The heterozygous *Esg1^‐tdTomato^* reporter cell line was derived from the *Stella*‐GFP:: *Esg1*‐tdTomato (SGET) compound reporter line (Hackett *et al*, 2018) by CRISPR inactivation of the *Stella*‐GFP reporter. For generating the *p53^‐tdTomato^* reporter cell line, we obtained T2A‐tdTomato dsDNA sequence by PCR amplification from a donor vector with ultramers carrying 180 bp overhangs complementary to the 3’ end of the *p53* gene. We introduced this into cells by transfection of 129/B6 XY ESC together with the spCas9 plasmid pX459 (Addgene #62988), carrying a single gRNA sequence complementary for the *p53* 3’ end. After antibiotic selection for transient px459, TOM^pos^ single cells were sorted at by FACS. Single cells were expanded clonally and correct monoallelic integration to generate *p53^‐tdTomato^* was validated by PCR genotyping and Sanger sequencing (Genewiz). Normal levels of *p53* mRNA expression were verified by qPCR.

### Epigenetic editing and memory assay

For stable integration of the epigenetic editing system, *Esg1^‐tdTomato^* or *p53^‐tdTomato^* WT or KO reporter ESC lines were co‐transfected with the Piggybac plasmids: pPB_TRE3G::dCas9^‐GCN4^_*EF1a::*TetOn‐Hygro, pPB_TRE3G::ScFv‐KRAB^‐GFP^_EF1a::Neo and pPB_U6::gRNA_EF1a::BFP‐Puro containing appropriate gRNA sequence and pPY_CAG_Pbase using 5:5:1:1 molar ratio respectively. Alternatively the pPB_TRE3G::KRAB^‐GFP‐scFv^_EF1a::Neo was replaced with a construct carrying only expression of *GFP^‐scFv^* pPB_TRE3G::ScFv‐GFP_EF1a::Neo as a control. Cells with successful integration of the three cassettes were enriched by successive selection with hygromycin (250 μg/ml) for 5 days, neomycin (300 μg/ml) for 3 days and puromycin (1.2 μg/ml) for 2 days. After 2 days of cellular recovery, expression of dCas9^‐GCN4^ and *KRAB^‐GFP‐scFv^* was induced with doxycyclin (DOX) (100 ng/ml) for 7 days, and double GFP‐ and BFP‐positive cells that had activated the epigenetic editing system were enriched by FACS. GFP/BFP double‐positive cells were re‐seeded into culture in absence of DOX and retention of reporter silencing estimated by flow cytometry after 4 or 7 days of DOX washout in cells that switched off the destabilised epigenetic editing tool gated as BFP^pos^/GFP^neg^ cells.

### Cell cycle inhibition

For cell cycle inhibition, to test active versus passive epigenetic erasure, the *Esg1^‐tdTomato^* cell line, already carrying the *dCas9^‐5XGCN4^* and *KRAB^‐GFP‐scFv^* or *GFP^‐scFv^*, was transiently transfected with a pPB_U6::gRNA_EF1a::BFP‐Puro containing gRNA against *Esg1* TSS (*gRNA^87dw^*). 1.2 ng/ml of puromycin selection was added after 6 h together with DOX (100 µg/ml). Cells were cultured for 3 days and then sorted for TOM^neg^ status. TOM^neg^ cells were then cultured in absence of DOX for a total of 4 days, with the cell cycle inhibitor RO3306 (9 μM) added after 24 h (when GFP had just switched off), and removed after 48 h. Cells were analysed by flow cytometry at 24 h intervals and gated for absence of expression of GFP and BFP.

### ESC differentiation

To induce endodermal, ectodermal or epiblast‐like cell (EpiLC) differentiation, naïve ESCs were cultured for 5 days in presence of DOX (100 ng/ml) seeded at a confluence of 6 × 10^3^/cm^2^ on gelatin‐coated plates (for endoderm and ectoderm) or fibronectin‐coated plates (for EpiLC) and maintained in t2i/L media for 24 h in presence of doxycycline (100 ng/ml). After removal of ESC medium and 5× washes with PBS, differentiation was induced as follows: (i) endoderm differentiation was induced with IDE1 (STEMCELL Technologies #72512)‐containing medium (Borowiak *et al*, 2009) (RPMI (Thermo Fisher Scientific #12‐633‐012) supplemented with 0.02% FBS, 2 mM L‐glutamine, 5 μM IDE1 and 1% penicillin streptavidin); (ii) ectoderm differentiation was induced with NDiff (NB27 Takara #y40002) supplemented with 0,25 μM Retinoic Acid (Sigma Aldrich#R2625), 0,02% FBS and 1% penicillin streptavidin and (iii) EpiLC differentiation was induced with NDiff supplemented with 20 ng/ml ActivinA (PeproTech #120‐14P), 12 ng/ml bFGF (PeproTech #450‐33), 1% knockout serum replacement (Thermo Fisher #10828010) and 1% penicillin streptavidin. In all cases, DOX treatment was maintained for the first 24 h of differentiation and then cells were washed five times and cultured for a maximum of 8 days without DOX, with media change every other day. Flow cytometry was performed at 5 and 8 days of differentiation (corresponding to 4 and 7 days of DOX washout, respectively) and, at the same time, cells harvested for bisulphite pyrosequencing and CUT&RUN.

### Growth competition assay

For assaying the competition advantage of the *p53* epigenetically silenced cells, *p53^‐tdTomato^* reporter line, carrying *dCas9^GCN4^*, *KRAB^‐GFP‐scFv^* and a gRNA against p53 (*gRNA^345up^*), was induced with DOX (100 ng/ml). In parallel, *p53^‐tdTomato^* reporter line without the epigenetic editing tool from a similar passage number was transfected with a PiggyBac plasmid driving constitutive GFP expression (CAG:GFP) and subjected to two subsequent rounds of sorting to enrich GFP^pos^ cells. After 7 days of DOX induction, KRAB‐induced *p53^‐tdTOM^*‐negative (TOM^neg^) cells were enriched by flow cytometry and equally mixed (1:1) with cells expressing constitutive GFP and subjected to DOX washout. After 5 or 8 days from mixing, cells were analysed by flow cytometry to measure the proportion of GFP^pos^ and GFP^neg^ cells.

### Generation of knockout ESC lines

Knockouts (KO) cell lines were generated by transiently transfecting two spCas9 plasmids (pX459) carrying one gRNA each targeting exon sequence for critical catalytic activity of the gene of interest (*Dppa2*, *Zmym3*, *Kmt2d*, *Smarcc1* and *Dot1L*) (Table EV1) in *Esg1^‐tdTomato^* reporter lines. Similarly, two *Dppa2* clonal *knockout* lines were generated in the TX1072 background. After transfection, cells were selected with puromycin (1.2 μg/ml) for 3 days and subsequently seeded at low density (1,000 cells per 9.6 cm^2^) for single colony picking. Subsequently to expansion, single clones were screened for bi‐allelic genetic deletion by PCR genotyping (Table EV2) and Sanger sequencing (Genewiz). For *Dppa2* ^−/−^, absence of the protein was further confirmed by western blot (Fig EV4A).

### Western blot

For protein extraction, cell pellets were resuspended in RIPA buffer (Sigma #R0278) containing protease inhibitors (Roche #4693159001), incubated at 4°C for 30 min and, upon centrifugation, cell lysis supernatant was collected. 10–20 μg of protein was mixed with bolt LDS sample buffer (ThermoFisher #B0007) and bolt‐reducing agent (ThermoFisher #B0004), heated at 70°C for 10 min and loaded on 4–12% Bis‐Tris gel (ThermoFisher #NW04125BOX). After electrophoresis separation at 200 V using MES running buffer (ThermoFisher #NP0002), proteins were transferred to a PVDF membrane (ThermoFisher #IB24002) using the iBlot 2 transfer stack (ThermoFisher) and the membrane was subsequently saturated with 5% milk/1xPBS for 1 h at room temperature. For detection of the protein of interest, the membrane was incubated at 4°C overnight with primary antibody (Table EV3) in 0.5%milk/PBS/0.05%tween and after three washes in PBS/0.05% tween, HRP‐linked secondary antibody incubation was carried on in 0.5%milk/PBS/0.05%tween for 1 h at room temperature. After washing thrice with 0.5%milk/PBS/0.05%tween, detection was performed by incubating the membrane with Pierce ECL western blot solution (ThermoFisher #32132) for 5 min prior imaging with ChemiDoc XRS+ system (BioRad).

### RNA preparation and real‐time qPCR

Total RNA was extracted using the PicoPure RNA Isolation kit (Applied Biosystems #KIT0204) for less than 1 × 10^4^ cells or the RNeasy kit (Qiagen #74004) otherwise, following manufacturer instructions. One microgram of RNA was used as input to generate complementary DNA (cDNA), with a mixture of random hexamers and reverse transcriptase, following DNAase treatment (TAKARA PrimeScript RT Reagent Kit with gDNA Eraser #RR047A). A control reaction in which the RNA was incubated with all the other components except the reverse transcriptase enzyme mix (‐RT control) was performed. cDNA was diluted 1:10 and specific targets quantified by real‐time quantitative qPCR using primers designed at exon–exon junctions to minimise amplification from contaminant DNA (Table EV2). The reaction was performed using SYgreen Blue Mix (PCRbio # PB20.15‐20) and a QuantStudio 5 (Applied Biosystems) thermal cycler.

### Bisulphite pyrosequencing

DNA bisulphite conversion was performed directly starting from cell pellets (a maximum of 1 × 10^5^ cells per sample) using the EZ DNA Methylation‐Direct kit (Zymo Research #D5021) following the manufacturer’s instructions. Target genomic regions were PCR amplified using 1 μl of converted DNA with biotin‐conjugated bisulphite primers (Table EV2), using the PyroMark PCR kit (Qiagen #978703). Pyrosequencing assay conditions were generated using the PyroMark Q24 Advanced 3.0 software and the sequencing reaction was performed with PyroMark Q24 advanced reagents (Qiagen, #970902) according to manufacturer’s instructions. Briefly, 10 μl of the PCR reaction was mixed with streptavidin beads (GE Healthcare #17‐5113‐01) by shaking for 5 min at room temperature and, after separation of DNA strands and release of samples into the Q24 plate (Qiagen) using PyroMark workstation (Qiagen), sequencing primers were annealed to DNA by heating at 80°C for 2 min and cooling down at RT for 5 min. Pyrosequencing was run on PyroMark Q24 advanced pyrosequencer (Qiagen) with target‐specific dispensation order (Table EV4). Results were analysed with PyroMark Q24 Advanced 3.0 software.

### CUT&RUN

The CUT&RUN (Cleavage Under Targets and Release Using Nuclease) protocol (Skene & Henikoff, 2017) was used to detect protein–DNA interaction and histone modifications. Briefly, a total of 3 × 10^5^ cells per sample were pelleted and washed twice with wash buffer (20 mM HEPES pH 7.5, 150 mM NaCl and 0.5 mM spermidine containing protease inhibitor) and incubated with conacavallin A magnetic beads (Sigma Aldrich C7555) by rotating for 10 min at room temperature. After placing samples on a magnet stand, the supernatant was removed. Cells were resuspended with antibody buffer (wash buffer with 0.02% digitonin and 2 mM EDTA) containing 0.5 μg of target‐specific antibody (Table EV3), and left rotating overnight at 4°C.

Samples were then placed on a magnet stand to remove antibody buffer, washed thrice with wash buffer containing 0.02% digitonin (Dig‐wash buffer), and incubated with 700 ng/ml of purified protein‐A::MNase fusion (pA‐MNase) on a rotor at 4°C for 1 h followed by two more washes. MNase reaction was thus activated by adding 4 mM CaCl_2_ and incubating at 0°C for 30 min and immediately stopped with 1× final concentration of STOP buffer (340 mM NaCl, 20 mM EDTA, 200 mM EGTA, 0.02% digitonin, 250 µg glycogen and 250 µg RNaseA). Target chromatin was released by incubating at 37°C for 10 min, centrifuging at full speed for 5 min at 4°C and the supernatant collected after incubation on magnet stand. DNA was finally released from chromatin by incubation with 0.4% SDS (Promega #V6551) and 0,5 mg/ml Proteinase K (Thermo Fisher Scientific #AM2546) at 70°C for 10 min. Purification and size selection of DNA were performed using SPRI beads (Beckman Coulter #B23318) following the instruction for double size selection with 0.5× and 1.3× bead volume‐to‐sample volume ratio. CUT&RUN DNA fragments were either subjected to quantitative PCR to amplify selected targets or to next‐generation sequencing to evaluate chromatin marks genome‐wide.

For CUT&RUN‐qPCR, DNA fragments were diluted 10 times with H_2_O and 2 μl amplified with SYgreen Blue Mix (PCRbio) and primers specific for target and control regions (in which the mark is expected to be enriched (positive controls) or depleted (negative controls)) (Table EV2) using the QuantStudio 5 (Applied Biosystems) thermal cycler. Note that primers were designed to amplify minimum amplicon sizes as CUT&RUN produces small fragments. Relative abundance of histone marks was estimated comparing Ct values of target regions to positive control regions.

For CUT&RUN sequencing, libraries were made starting from 10 ng of CUT&RUN DNA fragments using the NEBNext Ultra II DNA Library Prep Kit for Illumina (NEB #E7645S) using the following PCR programme: 98°C 30 s, 98°C 10 s, 65°C 10 s and 65°C 5 min, steps 2 and 3 repeated for 12–14 cycles depending on input DNA. After quantification and quality check with an automated electrophoresis system (Agilent Tape Station system), library samples were sequenced on the Nextseq Illumina sequencing system (paired‐end 40 sequencing). Raw Fastq sequences were trimmed to remove adaptors with TrimGalore (v0.4.3.1, ‐phred33 ‐‐quality 20 ‐‐stringency 1 ‐e 0.1 ‐‐length 20), quality checked and aligned to the custom mouse mm10 genome with the inserted tdTomato reporter using Bowtie2 (v2.3.4.2, ‐I 50 ‐X 800 ‐‐fr ‐N 0 ‐L 22 ‐i 'S,1,1.15' ‐‐n‐ceil 'L,0,0.15' ‐‐dpad 15 ‐‐gbar 4 ‐‐end‐to‐end ‐‐score‐min 'L,‐0.6,‐0.6'). Analysis of the mapped sequences was performed using seqmonk (Babraham bioinformatics, v1.46.0) by enrichment quantification of the normalised reads.

### *Xist* upregulation in TX1072 cells and RNAseq

To induce ectopic X‐chromosome inactivation, WT or *Dppa2* ^−/−^ female polymorphic BL6/Cast XX (TX1072) cell lines with DOX‐inducible *Xist* on the Bl6 allele were treated with DOX (1 μg/ml) for 6 days. To reverse Xist expression to normal levels, cells were washed with PBS 1× for three times and further cultured in absence of DOX up to 7 days. Cell pellets were harvested at the following time points: −DOX, +DOX (6 days), DOX washout (1, 3 and 7 days) and total RNA extracted using the Monarch Total RNA Miniprep Kit (New England Biolabs T2010S), following the manufacturer’s instructions. After quantification of total RNA with Qubit III and quality check with high‐sensitivity RNA Screen Tape (Agilent 5067‐5579) to ensure RIN > 8.5, 250 ng of RNA was used for library preparation for NGS sequencing with NEB next Ultra II Directional RNA protocol for Poly(A) mRNA magnetic Isolation Module (NEB #E7490) following the manufacturer guidelines. Multiplexed amplified libraries were sequenced on NextSeq500 (PE40).

For monoallelic analysis, reads were quality checked, mapped on mm10 (GRCm38) N‐masked genome for Cast SNPs with hisat2 (v. 2.2.1) and split by allele using SNPsplit (v. 0.5.0). Allele‐specific and the unassigned bam files were sorted and quantified using featureCount (parameters: ‐a <input>, ‐M enable multi‐mapping reads to be counted and ‐C chimeric reads not counted). Data were analysed with seqmonk software (v1.46.0) to generate log_2_ reads per million (RPM) using the RNA‐seq quantification pipeline for directional libraries.

### ATAC‐seq

Prior to harvesting, cells were initially treated in culture medium with 200 U/ml of DNase for 30 min at 37°C to digest degraded DNA released from dead cells. After 5× washes with 1xPBS, cells were detached with TrypLE, 5 × 10^4^, counted and pelleted at 500 RCF at 4°C for 5 min. Supernatant was removed and cells resuspended in 50 μl of cold ATAC resuspension BUFFER (10 mM Tris‐HCl pH7.4, 10 mM NaCl and 3 mM MgCl2) with 0.1% NP40, 0.1% Tween20 and 0.01% digitonin and incubated on ice for 3 min. Lysis was washed out using 1 ml of cold ATAC resuspension buffer with 0.1% Tween20 and mixed. Nuclei were pelleted at 500 RCF for 10 min at 4°C. After removal of supernatant, nuclei were resuspended in 50 μl of transposition mixture (25 μl 2xTD buffer, 2.5 μl transposase (Illumina Tagment DNA Enzyme and Buffer Kit #20034197), 16.5 μl PBS1x, 0.5 μl 1% digitonin, 0.5 μl 10% tween20 and 5 μl H_2_O) and incubated at 37°C for 30 min in a thermomixer with 1,000 RPM shaking. Reaction product was cleaned‐up with DNA clean and concentration kit (Zymo Research #D4014) following manufacturer instructions and eluted in 21 μl of elution buffer. Twenty microlitre of this product was used for PCR amplification using Q5 hot start high‐fidelity polymerase (NEB #M0494S) and a unique combination of the dual‐barcoded primers P5 and P7 Nextera XT Index kit (Illumina #15055293) following the cycling conditions: 1. 98°C for 30 s; 2. 98°C for 10 s; 3. 63°C for 30 s; 4. 72°C for 1 min; 5. 72°C for 5 min and repeat from 2 to 4 for five cycles. After the first five cycles, 5 μl of the pre‐amplified mixture was used to determine additional cycles by qPCR amplification using SYgreen Blue Mix (PCRbio) and the above used P5 and P7 primers in a QuantStudio 5 (Applied Biosystems) thermal cycler. After qPCR amplification, profiles were manually assessed plotting linear Rn versus cycle and the number of the additional PCR cycles to be performed equal to one‐third of the maximum fluorescent intensity in this plot (Buenrostro *et al*, 2015). The identified number of extra PCR cycles were performed by placing the pre‐amplified reaction back in the thermal cycler. Final clean‐up of the amplified library was performed using the DNA clean and concentration kit (Zymo #D4014) and DNA amplicons eluted in 20 μl of H_2_O. After quantification and quality check with an automated electrophoresis system (Agilent Tape Station system), library samples were pooled together and sequenced on the Nextseq Illumina sequencing system (paired‐end 40 sequencing).

For sequencing, raw reads were first trimmed with TrimGalore (v0.4.3.1, reads > 20 bp and quality > 30) and then quality checked with FastQC (v0.72). Output files were aligned to custom mouse mm10 genome with the inserted tdTomato reporter using Bowtie2 (v2.3.4.3, paired‐end settings, fragment size 0‐1,000, ‐‐fr, allow mate dovetailing). Uninformative reads were removed with Filter BAM (v2.4.1, mapQuality ≥ 30, isProperPair, !chrM) and duplicated reads were filtered with MarkDuplicates tool (v2.18.2.2). The mapped and filtered sequences were then analysed with seqmonk (Babraham bioinformatics, v1.46.0) by performing enrichment quantification of the normalised reads. Correlation plots were generated by comparing enrichment of reads at promoters in sample versus control conditions.

### Genome‐wide CRISPR screen

For the CRISPR screening, stable integration of *spCas9*‐T2A‐GFP was achieved in *Esg1^‐tdTomato^* reporter ESC by insertion into the *Rosa26* safe harbour locus by CRISPR targeting with a pair of *Rosa26*‐specific gRNAs. After antibiotic selection and single‐cell GFP^pos^ FACS sorting, integrity of the construct was verified by PCR genotyping and Sanger sequencing. The PiggyBac *dCas9^GCN4^* construct was subsequently introduced in these cells as described before and after single colony picking and expansion, successful integration of *dCas9^‐GCN4^* was functionally tested.

To introduce the genome‐wide perturbation, we used lentiviral vectors carrying the Brie gRNA library which contains 78,637 different gRNA that target 19,674 genes (Doench *et al*, 2016), produced as previously described (Carlini *et al*, 2020). Briefly, the pooled gRNA Brie library (Addgene #73632) was expanded in HEK 293T according to BSL2 guidelines, lentiviral‐containing supernatant was harvested and viral particles concentrated and resuspended in NDIFF 227. Lentiviral activity was estimated by transducing ESC across a titration curve and identifying a titration ratio to obtain 30–50% infection efficiency. 7 × 10^7^ *Esg1^‐tdTomato^* ESC containing the CAG::spCas9‐T2A‐GFP and *TRE3G::dCas9^‐GCN4^* was transduced in t2i/L medium with the pre‐determined volume of lentiviral particles to ensure ~50% efficiency (> 400‐fold gRNA coverage). After 24 h, we removed any residual lentiviral particles by five washes with PBS1X and we selected the cells using puromycin (1,2 μg/ml) for 7 days. Cells were then passaged before confluence, maintaining a minimum of 3.2 × 10^7^ cells to ensure gRNA library coverage (> 400‐fold coverage) and medium was changed daily for 1 week to give enough time for the knockouts to be generated.

Before introducing the epigenome editing tool, we inactivated the spCas9‐T2A‐GFP cassette by transfecting > 1 × 10^8^ KO pool cells with two tracr:crRNA (Table EV1) against two unique sequences in the *spCas9* that differs from *dCas9^GCN4^* using Xfect RNA transfection reagent (Takara #631450) according to manufacturer instructions. After 5 days from transfection, 3 × 10^7^ GFP^neg^ cells were sorted with FACS to select the cells with inactivation of the *spCas9*, plated back in t2i/L 10%FBS and further expanded for 3 days. To introduce the epigenetic perturbation, 2 × 10^8^ *Esg1^tdTomato^* KO library cell line already carrying dCas9*^GCN4^* was transfected with *pPB_TRE3G::KRAB^‐GFP‐scFv^_EF1a::Neo* and *pPB_U6::gRNA_EF1a::BFP‐Puro* containing a gRNA against *Esg1* and *pPY_CAG_Pbase* using Xfect mESC transfection reagent (Takara #631320) and selection (neomycin (300 μg/ml) and DOX (100 ng/ml) induction was started after 24 h. Seven days post‐transfection, 3 × 10^7^ cells were sorted for TOM^neg^ and plated back in culture in absence of DOX. After 4 days of DOX washout, 3 × 10^7^ cells were sorted in parallel from the TOM^neg^ and TOM^pos^ fractions for genomic DNA extraction as an early time point (D‐wo (3 days)), using a gating strategy to separate fully silenced cells (TOM^neg‐2.5%^) or cells ranging from fully to mildly silenced (TOM^neg‐wide^). At the same time, 3 × 10^7^ unsorted cells were passaged up to a total of 7 days of DOX washout and sorting have been repeated as before to separate TOM ^neg‐2.5%^, TOM^neg‐wide^ and TOM^pos^ for the final time point (D‐wo (7 days)). Genomic DNA was isolated from purified populations by using DNeasy blood and tissue kit (Qiagen # 69504) following manufacturer instruction including RNAse step.

DNA libraries were prepared from TOM ^neg‐2.5%^, TOM^neg‐wide^ and TOM^pos^ at D‐wo (3 days) and D‐wo (7 days) time points in multiple parallel reactions, each containing 500 ng of gDNA, with custom primers containing the P7 flow cell overhangs (5′‐CAAGCAGAAGACGGCATACGAGATNNNNNNNNGTGACTGGAGTTCAGACGTGTGCTCTTCCGATCTTCTACTATTCTTTCCCCTGCACTGT‐3′), including 8 bp barcode and P5 overhang (5′‐AATGATACGGCGACCACCGAGATCTACACTCTTTCCCTACACGACGCTCTTCCATCTTTGTGGAAAGGACGAAACACCG‐3′) using the Q5 hot start high‐fidelity polymerase (NEB #M0494S) for 22–24 cycles. sgRNA amplicons were purified using SPRI beads (Beckman Coulter #B23318) following the instruction for double size selection with 0.5× and 1.2× bead volume‐to‐sample volume ratio. Purified fragments were checked and quantified with a tape station automated electrophoresis system (Agilent). Equal amplified library amounts were pooled together into a multiplexed library and sequenced for single‐end 50 bp (SE‐50).

### Statistical analyses

For analysis of CRISPR screens, counting of sgRNA representation in the isolated subpopulation of cells was performed using the Model‐based Analysis of Genome‐wide CRISPR‐Cas9 Knockout (MAGeCK, v0.5.9) tool (Li *et al*, 2014). Reads were first trimmed using cutadapt (v1.15) (*cutadapt ‐g TTGTGGAAAGGACGAAACACCG*) and quality checked using FastQC and then, the gRNAs counts were normalised to total reads within the sample (MAGeCK ‐count ‐norm‐method total). Last, the TOM ^neg‐2.5%^ or TOM^neg‐wide^ were compared to the TOM^pos^ for each time point to identify significantly enriched/depleted gRNAs/genes with a false discovery rate (FDR) < 0.2, using the ‐test command in MAGeCK. Statistical analysis of replicate data including Cut&Run‐qPCR and RT‐PCR was performed using appropriate strategies in Prism GraphPad statistical software (v8.4.3).

### Embryo manipulation

All experiments involving mice were carried out in accordance with the approved protocol and guidelines by the laboratory animal management and ethics committee of the European Molecular Biology Laboratory (EMBL) under license. Prior to microinjection, *p53^‐tdTomato^* reporter ESC were transfected with dCas9^GCN4^, *p53_ gRNA^345up^* and *KRAB^GFP‐scFv^* or alternatively *GFP^‐scFv^* and treated with DOX for 7 days. ESC microinjection was performed by the Gene Editing & Embryology Facility of EMBL Rome, using E3.5 embryos derived from natural mating of C57BL/6J mice. Injected embryos were implanted back into pseudo‐pregnant foster mothers. All animals employed and procedures were in accordance with the gold standard Italian and European Union regulation guidelines and approved by the local ethical committee.

After 7 days from the injection, at the embryonic development day 10.5, the deciduum was collected from the uterus and put into a 6 cm dish with cold PBS+10% FBS. Embryos were then extracted from the decidua and moved to fresh PBS+10% FBS, placenta removed and cleaned from debris and tissue fragments. Individual embryos were moved in one well of a round‐bottom 96‐well plates (Corning #CLS3367) containing 50 μl of TrypLE and incubated at 37°C for 20 min and pipetted until the embryo is entirely dissociated in single cell. The single‐cell suspension was then diluted with 100 μl of PBS+1%FBS and spun down at 1,200 rpm for 5 min. Cell pellet was then resuspended in 300 μl of FACS medium (1xPBS+1%FBS) and filtered (BD, cup‐Filcons #340632) for quantitative flow cytometry analysis with Attune Nxt.

## Author contributions

**Jamie A Hackett:** Conceptualization; Formal analysis; Supervision; Funding acquisition; Writing—original draft; Project administration; Writing—review & editing. **Valentina Carlini:** Data curation; Formal analysis; Investigation; Writing—review & editing. **Cristina Policarpi:** Methodology.

In addition to the CRediT author contributions listed above, the contributions in detail are:

VC performed experiments, data analysis and co‐wrote the manuscript. CP provided experimental support. JAH performed data analysis, designed and supervised the study, and wrote the manuscript.

## Disclosure and competing interests statement

The authors declare that they have no conflict of interest.

## Supporting information



Expanded View Figures PDF

Table EV1

Table EV2

Table EV3

Table EV4

## Data Availability

All data derived from CRISPR screening, RNA‐seq, Cut&Run‐seq and ATAC‐seq have been deposited in the publicly available ArrayExpress database under the accession codes E‐MTAB‐10522 (http://www.ebi.ac.uk/arrayexpress/experiments/E‐MTAB‐10522/), E‐MTAB‐10523 (http://www.ebi.ac.uk/arrayexpress/experiments/E‐MTAB‐10523/), E‐MTAB‐10524 (http://www.ebi.ac.uk/arrayexpress/experiments/E‐MTAB‐10524/), E‐MTAB‐10525 (http://www.ebi.ac.uk/arrayexpress/experiments/E‐MTAB‐10525/), E‐MTAB‐11182 (http://www.ebi.ac.uk/arrayexpress/experiments/E‐MTAB‐11182/) and E‐MTAB‐11183 (http://www.ebi.ac.uk/arrayexpress/experiments/E‐MTAB‐11183/).
