# Supplementary material for: Epigenetic inheritance is gated by naïve pluripotency and Dppa2
Source: EMBO J. 2022 Feb 24;41(7):e108677. doi: 10.15252/embj.2021108677 (PMC8982627; doi:10.15252/embj.2021108677)
Supplement: Supplementary file 1 — Expanded View Figures PDF [file EMBJ-41-e108677-s004.pdf]

Expanded View Figures

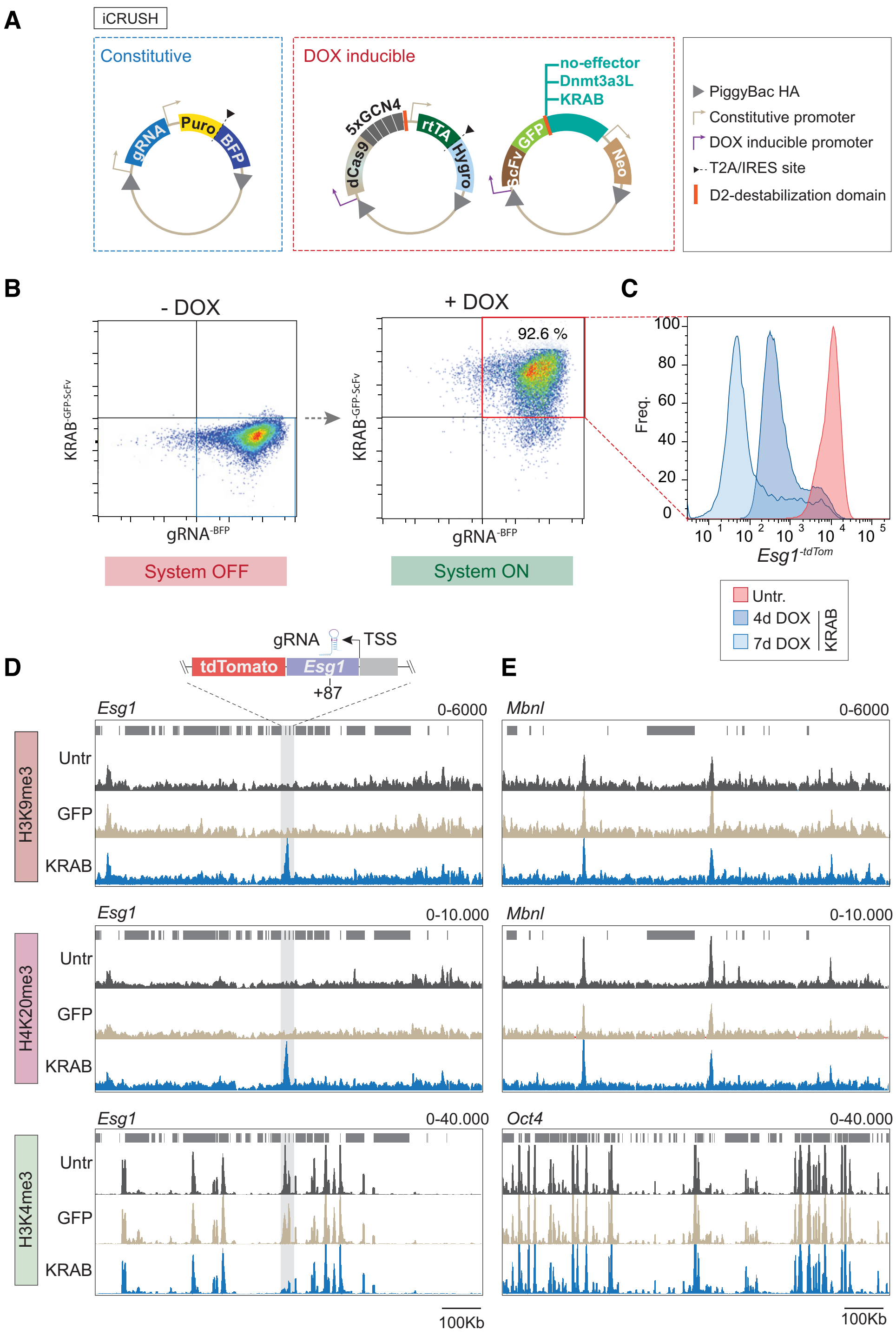

Figure EV1.

◀ **Figure EV1. iCRUSH induces domains of *de novo* histone modification levels comparable to endogenous heterochromatin loci.**

A PiggyBac constructs used to deliver the epigenetic editing tool (iCRUSH) into ESC. From left to right: the enhanced gRNA scaffold is under control of a constitutive U6 promoter, the same construct also drives constitutive expression of a puromycin selection gene and blue fluorescent protein (BFP) separated by the self-cleaving peptide T2A; the dCas9^{GCN4} fusion gene is under control of the TRE3G DOX-inducible promoter, the same construct also drives constitutive expression of the rtTA trans-activator and a hygromycin resistance gene separated by a T2A; the epigenetic effector constructs (KRAB$^{GFP\text{-}scFv}$ or Dnmt3a3L$^{GFP\text{-}scFv}$) are also under control of the TRE3G DOX-inducible promoter and it is fused with a green fluorescent protein (GFP) and an scFv domain specific for GCN4, it also drives constitutive expression of the neomycin resistance gene. These constructs are genomically integrated into ESC by co-transfection with the PiggyBac transposase.

B Density plots, obtained by flow cytometry analysis, show gRNA^{BFP} and KRAB$^{GFP\text{-}scFv}$ expression in the cell population prior to and upon DOX induction, revealing its dynamic activation.

C Fluorescence distribution measured by flow cytometry showing *Esg1* reporter silencing after recruitment of KRAB$^{GFP\text{-}scFv}$ for 4 or 7 days (blue) compared to a reference untargeted sample (red), demonstrating order of magnitude of silencing. Cells were gated for the presence of both KRAB$^{scFv\text{-}GFP}$ (GFP positive) and gRNA^{-BFP} (BFP positive).

D, E Genome views comparing the magnitude of *de novo* peaks of histone marks deposited by iCRUSH at the *Esg1* promoter (D) with endogenous representative examples (*Mbnl* and *Oct4*) (E).

 ©2022 The Authors

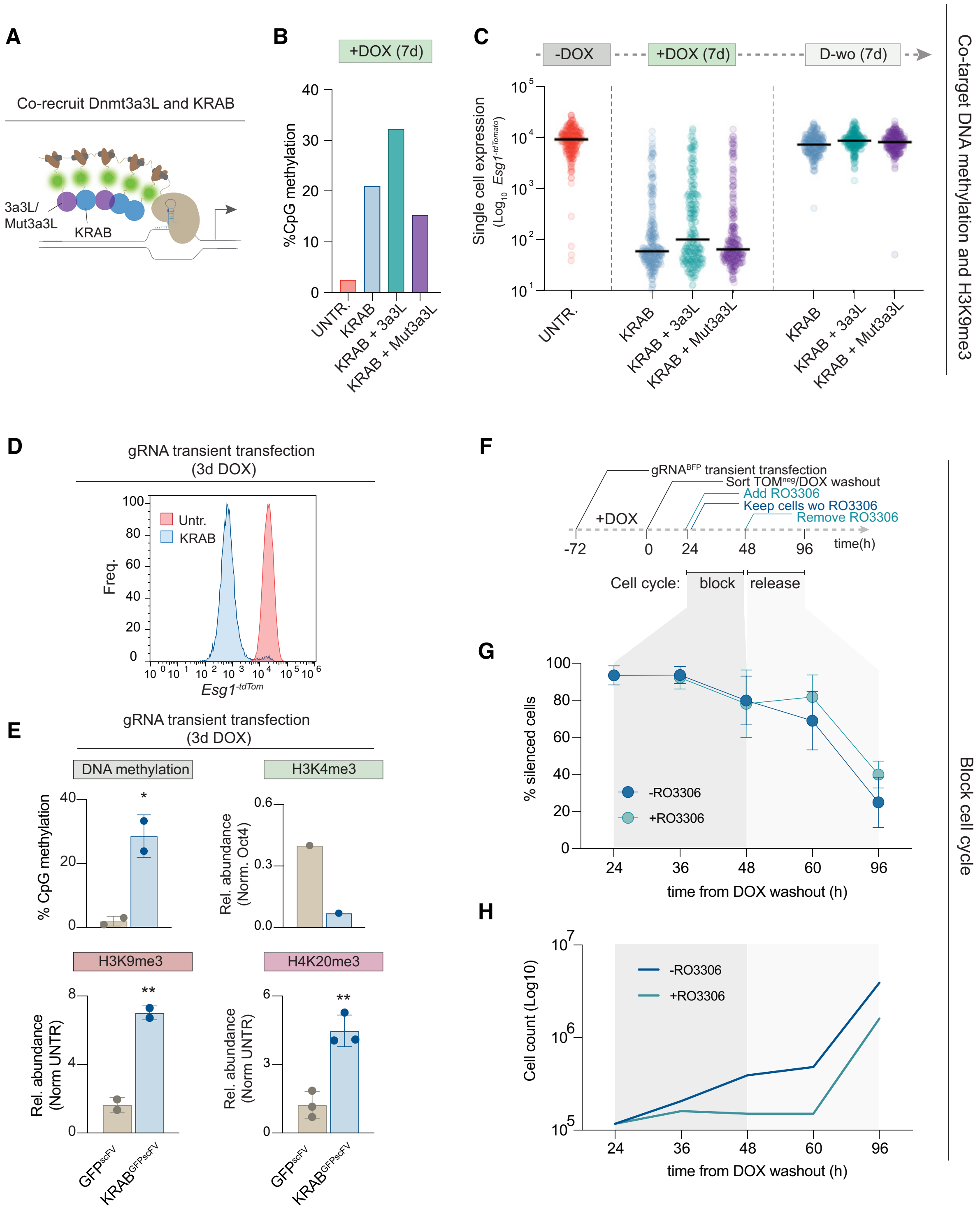

A
Co-recruit Dnmt3a3L and KRAB
3a3L/ Mut3a3L
KRAB
B
+DOX (7d)
%CpG methylation
UNTR.
KRAB
KRAB + 3a3L
KRAB + Mut3a3L
C
-DOX
+DOX (7d)
D-wo (7d)
Single cell expression
Co-target DNA methylation and H3K9me3
D
gRNA transient transfection (3d DOX)
Untr.
KRAB
Freq.
E
gRNA transient transfection (3d DOX)
DNA methylation
H3K4me3
H3K9me3
H4K20me3
% CpG methylation
Rel. abundance (Norm. Oct4)
Rel. abundance (Norm UNTR)
F
transient transfection
Sort TOM/DOX washout
Add RO3306
Keep cells wo RO3306
Remove RO3306
+DOX
time(h)
Cell cycle:
block
release
G
% silenced cells
-RO3306
+RO3306
time from DOX washout (h)
Block cell cycle
H
Cell count (Log10)
-RO3306
+RO3306
time from DOX washout (h)

Figure EV2.

Figure EV2. Cell cycle inhibition slows but does not block epigenetic erasure in naïve ESC.

A Schematics of the iCRUSH epigenetic tool used in the experiment. KRAB$^{GFP\text{-}scFv}$ is either recruited alone or in combination with Dnmt3a/Dnmt3L (3a3L$^{GFP\text{-}scFv}$) or the catalytically mutant Mut3a3L$^{GFP\text{-}scFv}$.

B Bar plot showing the average of the percentage DNA methylation at 6 CpG sites on the *Esg1* promoter assayed by bisulphite pyrosequencing.

C Violin plots show the single-cell distribution of *Esg1*$^{-tdTomato}$ expression (log scale) in each condition (−DOX, 7 days +DOX and 7 days DOX washout) upon epigenetic editing with KRAB$^{GFP\text{-}scFv}$ (blue) +/− 3a3L$^{GFP\text{-}scFv}$ (aqua green) or Mut3a3L$^{GFP\text{-}scFv}$ (purple). Each data point indicates a cell, and horizontal lines represent the median.

D *Esg* $^{-tdTomato}$ reporter expression in KRAB$^{GFP\text{-}scFv}$ or untransfected control after gRNA transient transfection and 3 days of DOX induction.

E Bar plots show percentage of CpG methylation or relative abundance of histone modifications normalised to a positive control region and untransfected control after 3 days of DOX (gRNA transiently transfected) on one, two or three biological replicates. Statistics calculated by unpaired *t*-test over two or three independent biological replicates (**P* < 0.05; ***P* < 0.01). Error is measured as ± SD.

F Timeline of the experiment. Briefly, cells are treated for 72 h (3 days) with DOX to induce iCRUSH-mediated silencing. *tdTomato*-negative cells are sorted and plated back in culture in duplicate experiments without DOX. After 24 h of DOX washout, one sample is treated with the cell cycle inhibitor RO3306 for further 24 h. After a total of 48 h from DOX washout, the cell cycle is released and cells further analysed at 12 h interval up to 96 h. In parallel, cells are cultured in the absence of the inhibitor.

G Time course of the percentage of *Esg1*$^{-tdTomato}$-silenced cells in the + or -RO3306 conditions at 12 h intervals of DOX washout. Error is measured as ± SD between two biological replicates.

H Line plots indicate log growth of cells in + or − RO3306 conditions.

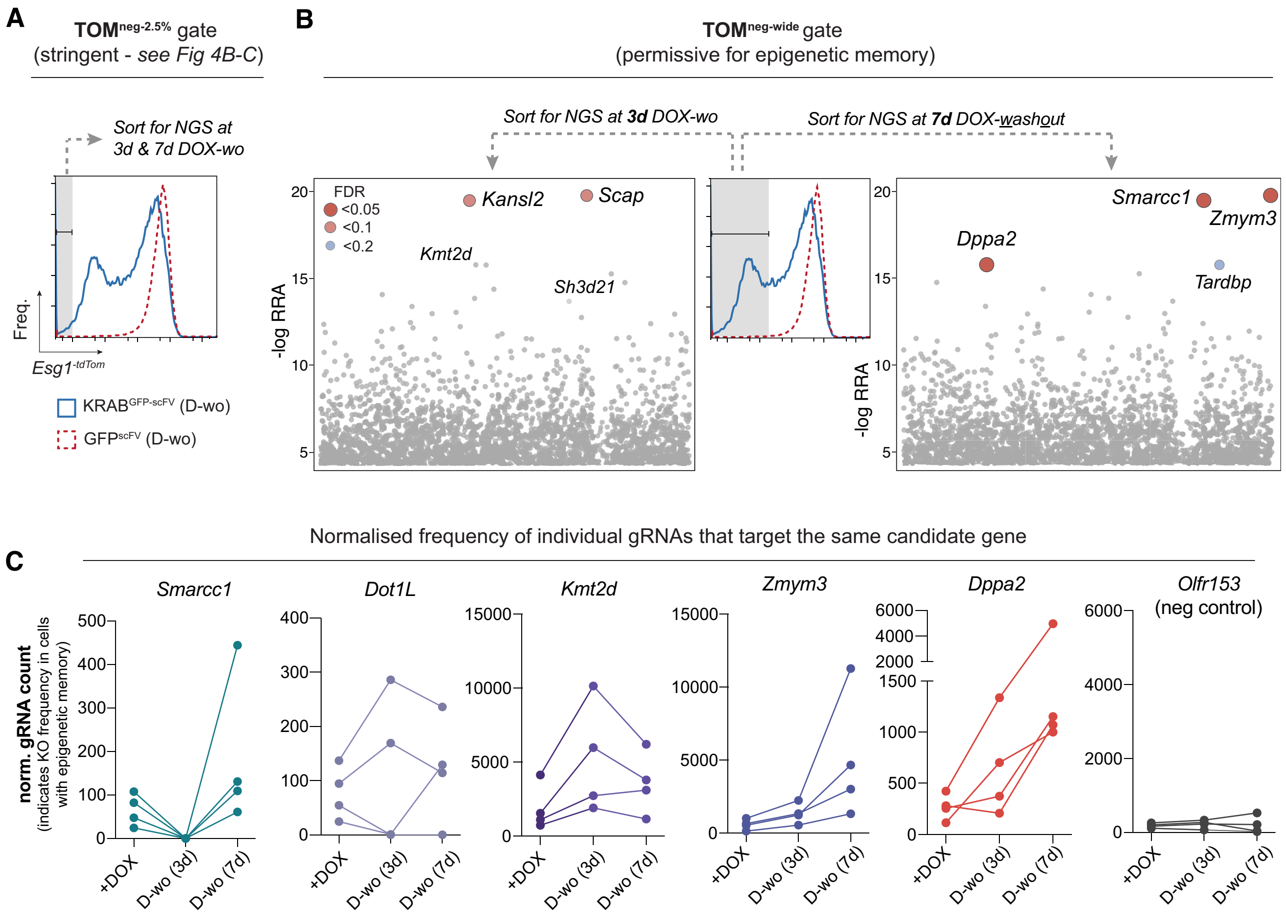

Figure EV3.

©2022 The Authors

Figure EV3. Gating strategy for CRISPR screen and supplementary scatterplots of candidate factors that antagonise epigenetic inheritance.

A Histogram showing the gate used to sort cells that strictly retained epigenetic silencing memory in the CRISPR screen (tdTomato negative) using a stringent gating threshold (bottom 2.5% TOM- cells ($TOM^{2.5\%neg}$). The results of significant candidate genes that enable this memory when knocked out are detailed in main Fig 4B and C.

B Middle histogram: A wider gate was used to include all tdTomato-negative cells ($TOM^{negwide}$) and was designed according to a tdTomato-positive control sample to capture all cells exhibiting full or partial silencing memory. Scatterplots: show significant hits from the screen displayed by -log relative ranking algorithm (RRA) score from the cells captured using the $TOM^{negwide}$ gate at short-term time point (3 days DOX washout; left) or longer term (7 aysd DOX washout; right). These hits are linked with enabling epigenetic inheritance when abrogated.

C Line plot for gRNA count from the CRISPR screen after silencing (+DOX) and during memory phase (DOX washout for 3 or 7 days) for the candidate genes indicated. Each line represents a different gRNA from the pool for the same target gene. A concordant enrichment during DOX washout indicates all gRNAs (and therefore independent knockouts of the target gene) promote epigenetic inheritance. Note *Kmt2d* is only enriched at the earlier time point.

©2022 The Authors

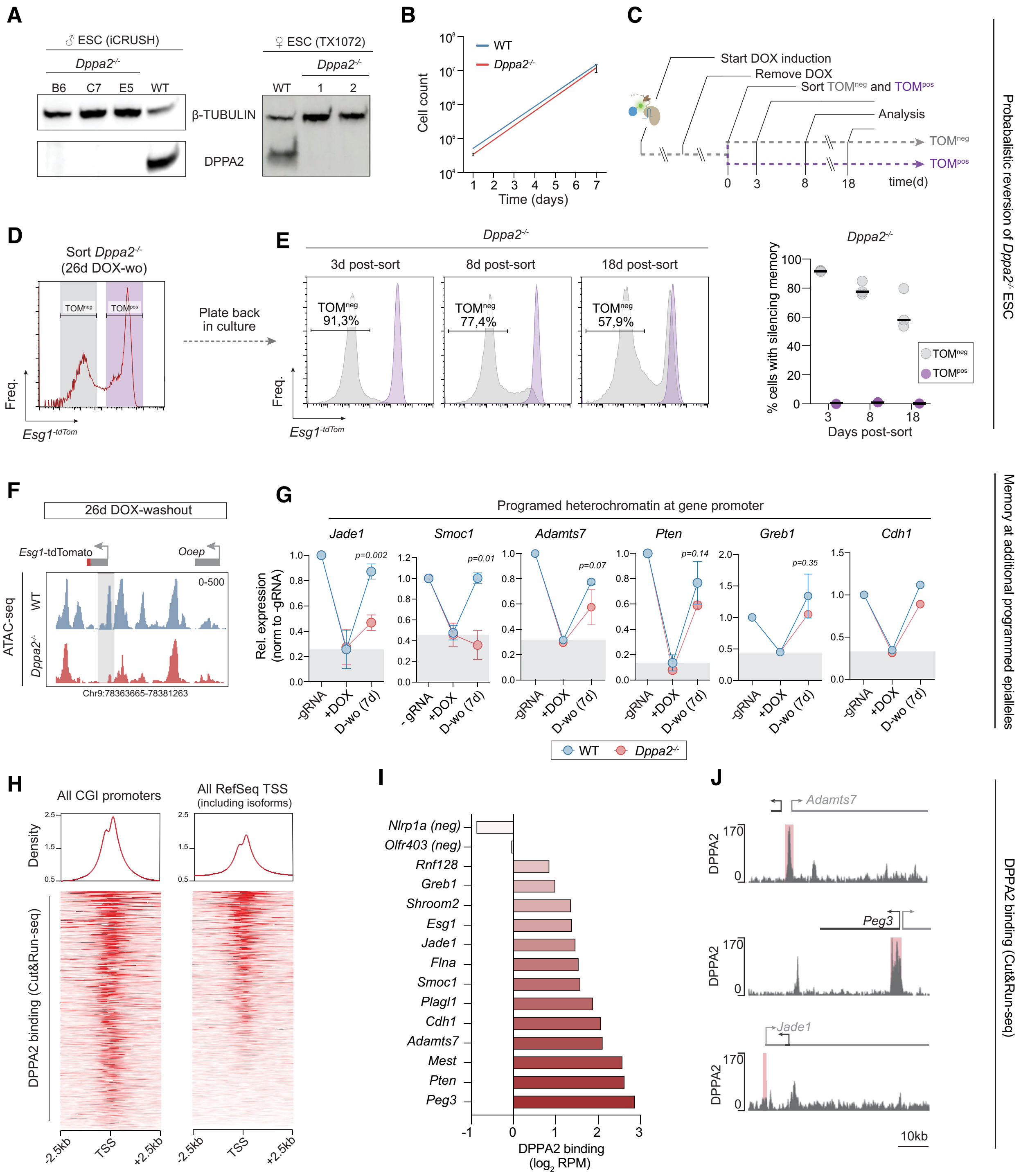

Figure EV4.

 ©2022 The Authors

Figure EV4. Epigenetic inheritance is probabilistic upon Dppa2 knockout.

A Western blot comparing DPPA2 protein expression in WT and *Dppa2* knockout individual ESC clonal lines. β-TUBULIN is used as loading control.
B Line plot indicates exponential growth of wild-type or *Dppa2*$^{-/-}$ cells.
C Timeline of the experiment in D and E. After induction for 7 days, tdTomato-negative (TOM^{neg}) and -positive (TOM^{pos}) fractions of cells are sorted following 26 days of DOX washout in *Dppa2*$^{-/-}$ and put back in culture separately. Flow cytometry analysis is performed after 3, 8 or 18 days after sorting.
D *Esg1*$^{-tdTomato}$ expression in *Dppa2*$^{-/-}$ after 26 days of DOX washout. Grey and purple boxes indicate the gates used to sort TOM^{neg} and TOM^{pos} fraction of cells respectively.
E Left, distributions of the TOM^{neg} and TOM^{pos} cell population over time, showing a probabilistic reversion to active status that is cumulative, indicating a stochastic memory function. Right, percentage of silenced cells of TOM^{neg} and TOM^{pos} in three independent *Dppa2*$^{-/-}$ clones, after 3, 8 or 18 days post-sort.
F ATAC-seq tracks showing chromatin accessibility at *Esg1-tdTomato* after 26 days of DOX washout in wild-type or *Dppa2*$^{-/-}$ lines. Grey box highlights *Esg1* promoter.
G Line plots show gene repression (+DOX) and memory (D-wo) of the indicated targets relative to the housekeeping gene *Rplp0* and normalised to -gRNA control. Grey boxes represent the level of silencing achieved after +DOX treatment in the *wild-type* condition. All non-imprinted targets exhibit increased memory in *Dppa2* knockout, albeit insignificant, which likely reflects stochastic memory function at the single-cell level (i.e. > 50% of the cells still revert in *Dppa2*$^{-/-}$, thereby blunting the effect in population measurements). Error bars are ± SD out of two or three biological replicates. Statistics is measured by one-tail unpaired *t*-test between WT and *Dppa2* knockout conditions.
H Distribution of DPPA2 binding (CUT&RUN-seq) over TSS ± 2.5 kb considering CG dense promoters or all promoters.
I Histograms showing DPPA2 occupancy (CUT&RUN-seq) at the targets reported in (G) and Fig 5H.
J Chromosome tracks of representative examples from (I).

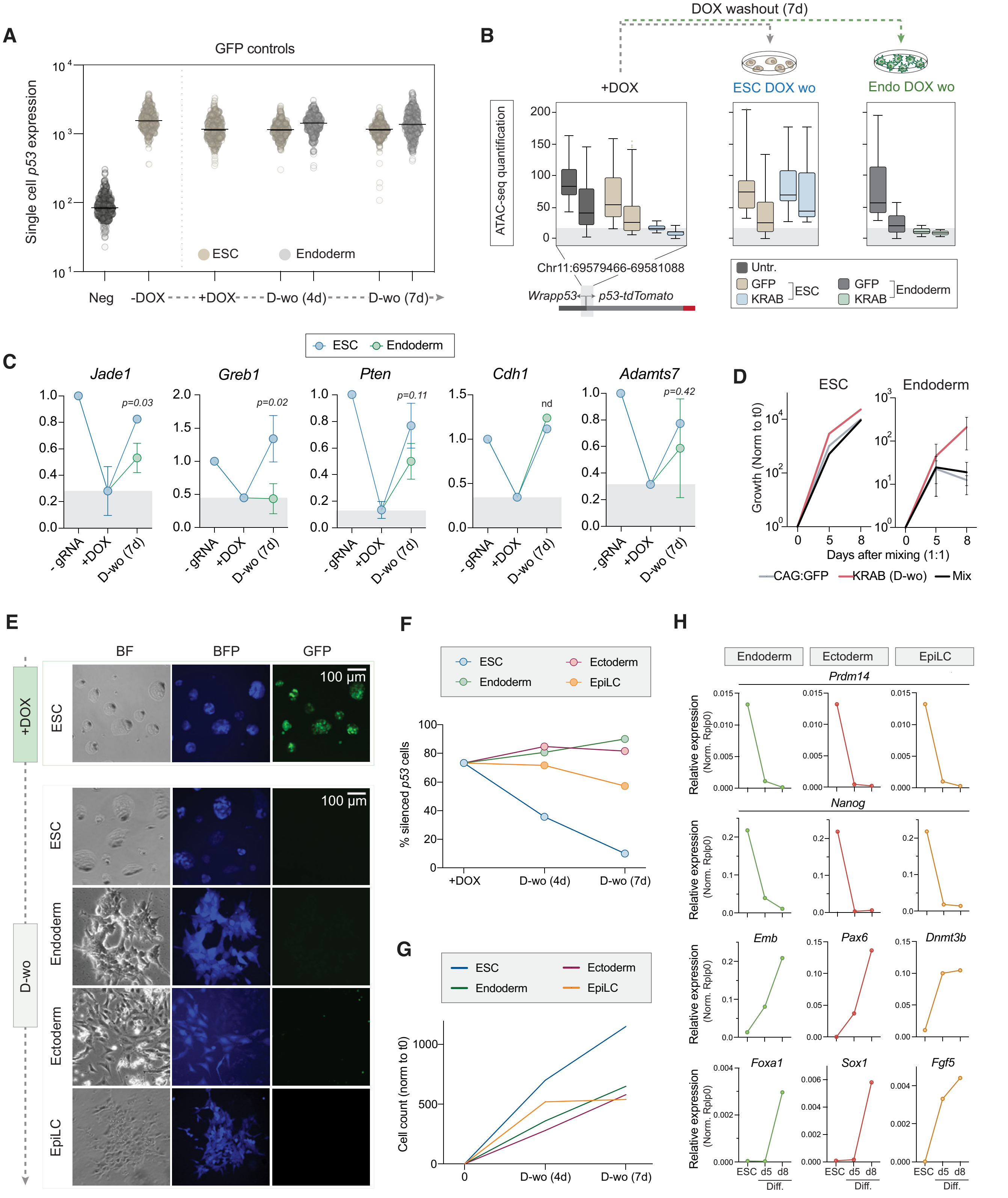
A
GFP controls
Single cell p53 expression
ESC
Endoderm
Neg
-DOX
+DOX
D-wo (4d)
D-wo (7d)
B
DOX washout (7d)
+DOX
ESC DOX wo
Endo DOX wo
ATAC-seq quantification
Chr11:69579466-69581088
Wrapp53
p53-tdTomato
Untr.
GFP
KRAB
ESC
Endoderm
C
ESC
Endoderm
Jade1
Greb1
Pten
Cdh1
Adamts7
p=0.03
p=0.02
p=0.11
nd
p=0.42
- gRNA
+DOX
D-wo (7d)
D
ESC
Endoderm
Growth (Norm to t0)
Days after mixing (1:1)
CAG:GFP
KRAB (D-wo)
Mix
E
BF
BFP
GFP
+DOX
D-wo
ESC
Endoderm
Ectoderm
EpiLC
100 µm
F
ESC
Endoderm
Ectoderm
EpiLC
% silenced p53 cells
+DOX
D-wo (4d)
D-wo (7d)
G
ESC
Endoderm
Ectoderm
EpiLC
Cell count (norm to t0)
0
D-wo (4d)
D-wo (7d)
H
Endoderm
Ectoderm
EpiLC
Prdm14
Nanog
Emb
Pax6
Dnmt3b
Foxa1
Sox1
Fgf5
Relative expression
(Norm. Rplp0)
ESC
d5
d8
Diff.

Figure EV5.

Figure EV5. Epigenetic memory is maintained over differentiation programmes representative of the three germ layers.

A Expression of the *p53*$^{-tdTomato}$ reporter at the single-cell level in ESC or endoderm-differentiated cells in the DOX condition indicated, using only the GFP control, confirming no effect (compared to main Fig 6B). Black horizontal bars represent the median of fluorescence intensity in the population of cells (single dots).

B Box plot quantification of the ATAC-seq accessibility at the *p53* promoter region in two biological replicates side by side. Boxes represent upper and lower quartile out of 800 values, horizontal bar is the median. Whiskers are the 5th to 95th percentiles.

C Line plots show expression of the indicated targets relative to the housekeeping gene *Rplp0* and normalised to -gRNA control for each time point in ESC or endoderm cells. They demonstrate most loci do not exhibit memory of epigenetic silencing in ESC or endoderm, but some (e.g. *Jade1* and *Greb1*) show selective epigenetic inheritance in endoderm. Grey boxes represent the level of repression achieved after DOX treatment in the wild-type condition. Error bars are standard deviation out of two or three biological replicates. Statistics is measured by unpaired *t*-test between WT and *knockout* conditions.

D Line plots indicate exponential growth of CAG:GFP, KRAB$^{-GFP-scFv}$ (D-wo) or the mixed population in one ESC or three endoderm cell lines used for the experiment in Fig 6F. Error bars are ± SD.

E Representative microscopy images of bright field and BFP or GFP fluorescence of ESC, endoderm, ectoderm or EpiLC-differentiated cells upon DOX induction (7 days) or DOX washout (7 days).

F Time course showing epigenetic memory of *p53-tdTomato* silencing during DOX washout in ESC, endoderm, ectoderm and EpiLC.

G Line plots show ratio of cell count normalised to time point 0 in ESC, endoderm, ectoderm or EpiLC-differentiated cells.

H Time course showing relative expression of naïve pluripotency (*Prdm14* and *Nanog*) or differentiation markers (*Emb* and *Foxa1* for endoderm; *Pax6* and *Sox1* for Ectoderm; *Dnmt3b* and *Fgf5* for EpiLC) compared to the housekeeping gene *Rplp0* in ESC and during differentiation programmes after release of the silencing trigger on *p53-tdTomato* reporter.

©2022 The Authors
