# Supplementary material for: Epigenetic inheritance is gated by naïve pluripotency and Dppa2
Source: EMBO J. 2022 Feb 24;41(7):e108677. doi: 10.15252/embj.2021108677 (PMC8982627; doi:10.15252/embj.2021108677)
Supplement: Supplementary file 6 [file EMBJ-41-e108677-s002.pdf]

THE EMBO JOURNAL

Epigenetic Inheritance is Gated by Naïve Pluripotency and Dppa2

Valentina Carlini, Cristina Policarpi, Jamie A. Hackett
DOI: 10.15252/embj.2021108677

Corresponding author: Jamie Hackett (jamie.hackett@embl.it)

Review Timeline:

Submission Date:	8th May 21
Editorial Decision:	14th Jul 21
Revision Received:	12th Nov 21
Editorial Decision:	13th Dec 21
Revision Received:	12th Jan 22
Accepted:	17th Jan 22

Editor: Daniel Klimmeck

Transaction Report:

(Note: With the exception of the correction of typographical or spelling errors that could be a source of ambiguity, letters and reports are not edited. Depending on transfer agreements, referee reports obtained elsewhere may or may not be included in this compilation. Referee reports are anonymous unless the Referee chooses to sign their reports.)

Dear Dr Hackett,

Thank you for the submission of your manuscript (EMBOJ-2021-108677) to The EMBO Journal. Please accept my sincere apologies for getting back to you with unusual protraction. Your study has been sent to three reviewers for evaluation. However, one referee got much delayed and, in the end, was not able to send us his-her report. In the interest of the timeliness of the data, we decided to base our decision on the two other reports, which I enclose below.

As you will see, the referees acknowledge the potential interest and value of your findings, although they also express major concerns. In more detail, the reviewers state substantial issues regarding the generality and depth of evidence supporting the claims made on Dppa2 involvement in epigenetic memory reversal (ref#1,2) and potentially confounding role of 2i culture on DNA hypomethylation (ref#1,2). Further, reviewer #1 remains critical on the advance provided by the iCRUSH methodology. In addition, the referees raise a number of issues related to additional controls and data points required, methods annotation, statistics and data illustration, discussion of results and literature references that would need to be conclusively addressed to achieve the level of robustness and clarity needed for The EMBO Journal.

Given the interest stated, we are overall able to invite you to revise your manuscript experimentally to address the referees' comments, along the lines sketched in your outline. However, we concur with the experts that providing support on the generality of your results and further clarifying the role of DNA methylation in ESC epigenetic memory maintenance should be key points for continued consideration at the EMBO Journal. As to the open outcome of the proposed experiments, we suggest keeping EMBO Reports in mind for this work as an alternative venue.

Please feel free to contact me if you have any questions or need further input on the referee comments.

We generally allow three months as standard revision time. As a matter of policy, competing manuscripts published during this period will not negatively impact on our assessment of the conceptual advance presented by your study. However, we request that you contact the editor as soon as possible upon publication of any related work, to discuss how to proceed. Should you foresee a problem in meeting this three-month deadline, please let us know in advance and we may be able to grant an extension.

When submitting your revised manuscript, please carefully review the instructions below, as they need to be complied with in order to trigger re-review of the manuscript.

Thank you for the opportunity to consider your work for publication.
I look forward to your revision.

Kind regards,

Daniel Klimmeck

Daniel Klimmeck, PhD
Senior Editor
The EMBO Journal

Instruction for the preparation of your revised manuscript:

1) a .docx formatted version of the manuscript text (including legends for main figures, EV figures and tables). Please make sure that the changes are highlighted to be clearly visible.

2) individual production quality figure files as .eps, .tif, .jpg (one file per figure).

3) a .docx formatted letter INCLUDING the reviewers' reports and your detailed point-by-point response to their comments. As part of the EMBO Press transparent editorial process, the point-by-point response is part of the Review Process File (RPF), which will be published alongside your paper.

4) a complete author checklist, which you can download from our author guidelines (https://wol-prod-cdn.literatumonline.com/pb-assets/embo-site/Author Checklist%20-%20EMBO%20J-1561436015657.xlsx). Please insert information in the checklist that is also reflected in the manuscript. The completed author checklist will also be part of the RPF.

5) Please note that all corresponding authors are required to supply an ORCID ID for their name upon submission of a revised

manuscript.

6) It is mandatory to include a 'Data Availability' section after the Materials and Methods. Before submitting your revision, primary datasets produced in this study need to be deposited in an appropriate public database, and the accession numbers and database listed under 'Data Availability'. Please remember to provide a reviewer password if the datasets are not yet public (see https://www.embopress.org/page/journal/14602075/authorguide#datadeposition).
In case you have no data that requires deposition in a public database, please state so in this section. Note that the Data Availability Section is restricted to new primary data that are part of this study.
*** Note - All links should resolve to a page where the data can be accessed. ***

7) Our journal encourages inclusion of *data citations in the reference list* to directly cite datasets that were re-used and obtained from public databases. Data citations in the article text are distinct from normal bibliographical citations and should directly link to the database records from which the data can be accessed. In the main text, data citations are formatted as follows: "Data ref: Smith et al, 2001" or "Data ref: NCBI Sequence Read Archive PRJNA342805, 2017". In the Reference list, data citations must be labeled with "[DATASET]". A data reference must provide the database name, accession number/identifiers and a resolvable link to the landing page from which the data can be accessed at the end of the reference. Further instructions are available at .

8) We would also encourage you to include the source data for figure panels that show essential data. Numerical data can be provided as individual .xls or .csv files (including a tab describing the data). For 'blots' or microscopy, uncropped images should be submitted (using a zip archive or a single pdf per main figure if multiple images need to be supplied for one panel). Additional information on source data and instruction on how to label the files are available at .

9) We replaced Supplementary Information with Expanded View (EV) Figures and Tables that are collapsible/expandable online (see examples in https://www.embopress.org/doi/10.15252/embj.201695874). A maximum of 5 EV Figures can be typeset. EV Figures should be cited as 'Figure EV1, Figure EV2" etc. in the text and their respective legends should be included in the main text after the legends of regular figures.

- For the figures that you do NOT wish to display as Expanded View figures, they should be bundled together with their legends in a single PDF file called *Appendix*, which should start with a short Table of Content. Appendix figures should be referred to in the main text as: "Appendix Figure S1, Appendix Figure S2" etc. See detailed instructions regarding expanded view here: .

- Additional Tables/Datasets should be labelled and referred to as Table EV1, Dataset EV1, etc. Legends have to be provided in a separate tab in case of .xls files. Alternatively, the legend can be supplied as a separate text file (README) and zipped together with the Table/Dataset file.

10) When assembling figures, please refer to our figure preparation guideline in order to ensure proper formatting and readability in print as well as on screen:
http://bit.ly/EMBOPressFigurePreparationGuideline

Please remember: Digital image enhancement is acceptable practice, as long as it accurately represents the original data and conforms to community standards. If a figure has been subjected to significant electronic manipulation, this must be noted in the figure legend or in the 'Materials and Methods' section. The editors reserve the right to request original versions of figures and the original images that were used to assemble the figure.

11) For data quantification: please specify the name of the statistical test used to generate error bars and P values, the number (n) of independent experiments (specify technical or biological replicates) underlying each data point and the test used to calculate p-values in each figure legend. The figure legends should contain a basic description of n, P and the test applied. Graphs must include a description of the bars and the error bars (s.d., s.e.m.).

Further information is available in our Guide to Authors: https://www.embopress.org/page/journal/14602075/authorguide

Revision to The EMBO Journal should be submitted online within 90 days, unless an extension has been requested and approved by the editor; please click on the link below to submit the revision online before 12th Oct 2021:

Link Not Available

Referee #1:

In this work, Carlini et al. generated a dox inducible Cas9-derived system to target heterochromatin at defined loci. The first part of the work showed that targeting of KRAB is able to establish heterochromatin (increase of H3K9me3 and DNA methylation, loss of H3K4me3) and transcriptional silencing at the endogenous gene Esg1 fused to tdTomato. Withdrawal of dox resulted in a rapid switch off of the induced silencing at Esg1 and 7 days after all cells reverted Esg1 to the ON state. Similar results were obtained at p53 and other genes (Pten, Cdh1, Greb1, Adamts7 and Jade1). In contrast, the imprinted genes Peg3, Mest and Plagl1 were able to maintain their silence sates. The authors then tested whether there was an active mechanism for the erasure of silencing. They perform an elegant CRISPR screen using as readout the expression of the endogenous reporter gene Esg1- tdTomato and found that Dppa2 was required to maintain silencing at Esg1 reporter gene. However, with the exception of Jade1, all other genes were not affected by the loss of Dppa2 (Fig. S5F). Thus, I find an overstatement declaring that "We observed propagation of repression at Pten, Cdh1, Greb1 and Adamts7 specifically in the absence of Dppa2 (Fig. S5F). Whilst this did not reach significance, this likely reflects an incompletely penetrant memory at the single cell level, similar to Esg1." The changes were minimal and non-significant. Thus, the data say that there is a difference between these genes relative to Dppa2 and that Dppa2 acts as an epigenome 'surveyor' only for two of the analyzed genes. The authors should have investigated why there is such difference.
Next, the authors asked whether the reversal of ectopic silencing at endogenous loci was a feature of naïve ESCs. They analyzed p53 in differentiated cells (i.e. upon endoderm induction) and found that ectopic silencing of p53 could be maintained, in particular DNA methylation whereas H3K9me3 could not. The authors also found that p53 silencing of ectoderm cells represent a growth advantage that enables the propagation of ectopic p53 silencing within the population.
The authors stated that "These data imply that differentiated cells, but not naïve pluripotent ESC, are competent for epigenetic inheritance of ectopic heterochromatin". However, the authors did not take into consideration the important point that naïve ESC that are cultured in 2i have low DNA methylation activity since the de novo DNMT3A and 3B are downregulated. Consequently, with the exception of the imprinted genes, the genome of naïve ESCs is hypomethylated. Thus, the difference in the epigenetic memory observed between naïve ESCs (treated with 2i) and differentiated cells is very luckily due to the differential DNA methylation activities. This point should have been discussed. Further, the authors should have tested whether ESCs cultured in serum, which have a fully functional DNA methylation machinery, can or cannot reverse ectopic silencing at endogenous loci. They also tested other genes and stated that "We observed that most regions reverted, albeit at least two (Greb1 and Jade1) exhibited robust inheritance of a prior silenced state in wildtype endoderm". This is an unclear statement. They tested 5 non-imprinted genes and only 2 of them exhibited robust inheritance of ectopic silencing, this is not "most of the genes".
The sentence concluding these results is very unclear and reflects the fact that the authors were not able to dissect the mechanisms of this reversal or inheritance of ectopic silencing: "Taken together, these data suggest that the potential for epiallele inheritance of de novo heterochromatin is influenced by multiple cell-type- and genomic- context dependent factors. In the case of p53, augmenting weak-acting heterochromatin inheritance in differentiated with a favourable advantage changes the balance of dynamic forces to enable propagation of ectopic chromatin states within the population." What about Greb1 and Jade1 in differentiated cells? Do they have also a growth advantage? Does Dppa2 affect the silencing inheritance at p53 in ESCs?
In summary, this work showed a nice dox inducible method to establish silencing at defined endogenous genomic loci, however, this methodology already existed. Further, they show the reversal of ectopic silencing, however, also here nothing new. Although the authors showed that Dppa2 is implicated in the reversal of ectopic silencing for Esg1 reporter and Jade1 in naïve ESCs, this seems to be a gene specific effect since the other genes are not affected by the lack of Dppa2. The authors failed to provide mechanistic insights of this process and did not investigate the role of DNA methylation that seems to be key in the studied system. The experiments and the methodology used are exceptionally well done. However, the biological readout of these experiments is unclear.

Minor points
Fig. 3B,C misses the X-axis description

Referee #2:

Carlini and colleagues established a cell line carrying an inducible dcas9 to target KRAB or DNMT3A/L (or combination) to a reporter Esg1 allele. They track transcriptional dynamics using this reporter to show that silencing is efficient when the system is induced by DOX after 7 days, and covers around 10kb from the sgRNA target site. They then do 'wash-off' experiments whereby they remove Dox to probe potentially inheritability of the silenced state. Using this approach they show that silencing is practically reverted 7 days later. Using a Crispr screening, they show that this reversal is mitigated in the absence of Dppa2. They then turn to another reporter, p53, and explore whether the silenced status of the p53 reporter is maintained during differentiation.

The manuscript deals with a very important topic in the epigenetics field, inheritability of transcriptional states, which is largely unexplored in mammals. This is therefore a very welcome effort, with a well-designed experimental set-up to address it. In general, I find the manuscript well executed and with appropriate controls, but somehow overstated in terms of the universality of their conclusions. I would recommend the manuscript for publication at EMBO Journal, provided that the following points are

addressed by the authors (please next time include page numbers !):

Major concerns:
1. Fig. 4F. There does not seem to be significant changes in promoter accessibility beyond Esg1. However, Wolf Reik group has documented significant changes in gene expression in Dppa2-depleted cells by siRNA (including e.g. 2C genes; Genes Dev 2019) - and therefore I find the ATACseq analysis in Fig. 4F at odds with known expression changes upon Dppa2 loss. The authors should perform a differential accessibility/peaks analysis and provide information on additional loci, beyond Esg1, that change accessibility inDpa2-/- cells.

2. Along the same lines, how 'general/targeted' are the observations in the Dppa2 mutants, beyond the targeted iCRUSH system (e.g. beyond the reporter) in +Dox and after washout conditions. An analysis to address this is essential to distinguishing direct effects of KRAB targeting versus general changes in e.g. expression, K9me3 or accessibility due to Dppa2 loss overall. The authors present scRNAseq, but limit this to the tagged Esg1 and p53 alleles. What happens genome-wide? (and to the wild-type, non-targeted allele?, which in fact could be used as control).

3.What is the sgRNA used in Fig. 1B/C, and throughout? This is nowhere stated in the text. The TSS at +87bp shows clearly a repressive effect regardless of KRAB (fig. 1G) and therefore the distinction of which sgRNA is used throughout the manuscript is fundamental to the conclusions of the paper.

4. The authors culture ESCs in 2i (they actually say 1%FCS in the methods, is this a mistake?), which are DNA hypomethylated and express high levels of TET. This could in fact mechanistically explain the reversion of the silenced state. How would Fig. 1G and 2B look-like if the cells are grown in serum/LIF without 2i? (and ideally also K9me3 levels as in Fig. 1B?).

5. The authors data suggest that DNA methylation may be responsible to lock a silenced state. Would the dynamics of Esg1/tdTomato expression upon removal of Dox change in TKO cells? (Dnmt1,3a and 3b KO)

6. Since the ChIP data for Dppa2 is available (Eckersley-Maslin et al., NSMB 2020) it would be interesting to check whether Dppa2 actually binds to Esg1 and the other loci studied in this manuscript. That may give clues to understand the differences between 'classical' and imprinted loci.

7. Why did the authors not confirm the effect of Dppa2 on P53 loss of repression? All other genes presented in figure 2 are examined, but not p53. Also, they state that 'we observed propagation of repression at Pten, Cdh1, Greb1 and Adamts7'. However, the data in Fig S5F do not support that statement, as only Jade1 is statistically different. I agree with their conclusion that it is likely to be context-dependent but Dppa2 seems to be important for only 2 genes out of the 9 tested and this should be clearly spelled-out.

8. When looking at Fig 6D it seems that the 7% actually comes from an outlier, most of the other embryos retain only 0-0.5% tomato-negative cells. The data presented in Fig 6C is then not reflecting the actual data.

9. Along the same lines, Fig. 6E would suggest that in contrast to ESCs-differentiation in culture, in the embryo the cells do not maintain transcriptional repression (only 50% of the embryos have a phenotype). While I find the potential of some clonal inheritance might be interesting, this is really only limited to 7% of the cells (or perhaps even less). The discussion of the authors is therefore not balanced.

10. I find the manuscript in general overstated regarding the extent of their conclusions and should be toned down. For example, I find the last 10 lines of their introduction overstated, because of the following:
i)the authors do not explore 'normal development';
ii)the authors do not compare e.g. naïve/primed pluripotency, nor they follow their dynamics, to be able to conclude on naïve pluripotency (only because they use 2i for culture)
iii)the conclusion on 'only under selective pressure' is also not justified (see my comments below, regarding p53 role in monolayer differentiation for example)
iv) there is no examination of intergenerational inheritance and/or its propagation in mammals.

11. On page 2 of the results, the authors state that they observed bimodal distribution of Esg1 expression (Fig. 2B) - however, Fig. 2B shows a graded pattern of expression, not bimodal. Please correct (alternative, the authors could make tests for bimodality in their dataset, but the data looks quite clearly graded, not modal). Also, their conclusion on 'probabilistic reversion of epimutations' is, accordingly, not founded, as well as the quite extensive (and unnecessary in my view) discussion emerging from this conclusion.

12. In the proliferation inhibition experiment, the analysis of expression, DNA methylation etc, should be done at +60 hours, before the cells start to proliferate. Otherwise, the authors cannot conclude safely on the role of proliferation. Endogenous, non-targeted genes/alleles should be used as control here.

Minor comments:

1. Especially in EV figures some panels have only 1 replicate or information on replicate number is missing. Please state the number of replicates throughout and complete replicate number to >3 where needed.

2. Please provide additional details for the reporter cell lines used (for example, it is nowhere stated that this is a heterozygous reporter allele? - we are referred to a previous publication by last author, but then I assume the current ms does not harbour the Stella-GFP reporter?)

3. The authors report that combination of KRAB and DNMT3A/L domains does not enhance reporter repression or maintenance. This contrasts to the report by Nunez et al. (Cell 2021) in differentiated human cells. From this and the analysis of imprinted genes performed by the authors, it seems that DNA methylation is important in that process. The authors should discuss this.

4. For the screening, please described how many genes were targeted.

5. Controls for efficient differentiation into the 3 cell types in Fig. S7 should be shown. Along these lines, p53 is known to affect differentiation in monolayer cultures of ESCs (Shigeta 2013). How does the repression of p53 affect the interpretations of chimera contribution and differentiation results in Fig. 6? Please discuss.

6. In the abstract, the authors claim that they identified mechanisms of inheritance, but to this reviewer, the authors have not identified a mechanism, they identified molecular players, but not mechanism and I therefore suggest to rephrase.

Response to reviewer comments for *Carlini et al*.

Here we briefly summarise the main additional experiments that we have performed, whilst below we address every issue on a point-by-point basis: In summary, we have undertaken an extensive series of new and validatory experiments, and re-evaluated the conclusions throughout. Taken together, we believe these have addressed each of the key areas of concern and significantly improved the manuscript:

1.) We have performed a major line of additional experiments to address the generality of *Dppa2* function. Most prominently an entirely new Figure 3 is now added, whereby we used an inducible X-inactivation paradigm to epigenetically silence hundreds of genes in parallel in naïve ESC, and then track their reactivation (loss of epigenetic memory). This data further supports our conclusion that, unlike differentiated cells, naïve cells specifically antagonise inheritance of programmed heterochromatin silencing.

2.) Using the above inducible X-linked silencing paradigm, we generated new *Dppa2* knockout female lines, and traced their memory dynamics. We observe propagation of epigenetic memory specifically in absence of *Dppa2* at a subset of genes, confirming its role via an orthologous system, as now shown in an extended Figure 5. Separately, we have programmed and traced heterochromatin at additional loci and identified a further DPPA2 dependency.

3.) We have performed CUT&RUN-seq to map DPPA2 binding genome wide to investigate how its genomic occupancy influences the propensity for heterochromatin memory.

4.) We have repeated our programmed epiallele assays in serum/LIF ESC conditions to compare with 2i/L. This enables us to understand the functional role of global DNA methylation status on reversion of epialleles.

5.) We have used an inhibitor of DNA methylation after epiallele formation.

Referee #1

In this work, Carlini et al. generated a dox inducible Cas9-derived system to target heterochromatin at defined loci. The first part of the work showed that targeting of KRAB is able to establish heterochromatin (increase of H3K9me3 and DNA methylation, loss of H3K4me3) and transcriptional silencing at the endogenous gene Esg1 fused to tdTomato. Withdrawal of dox resulted in a rapid switch off of the induced silencing at Esg1 and 7 days after all cells reverted Esg1 to the ON state. Similar results were obtained at p53 and other genes (Pten, Cdh1, Greb1, Adamts7 and Jade1). In contrast, the imprinted genes Peg3, Mest and Plagl1 were able to maintain their silence sates. The authors then tested whether there was an active mechanism for the erasure of silencing. They perform an elegant CRISPR screen using as readout the expression of the endogenous reporter gene Esg1-tdTomato and found that Dppa2 was required to maintain silencing at Esg1 reporter gene.

We thank the reviewer for committing their time to review our manuscript and for their thoughtful comments, concluding that they consider the "*experiments and the methodology used are exceptionally well done*". Below we address each comment (C) fully with a detailed response (R).

C1: However, with the exception of Jade1, all other genes were not affected by the loss of Dppa2 (Fig. S5F). Thus, I find an overstatement declaring that "We observed propagation of repression at Pten, Cdh1, Greb1 and Adamts7 specifically in the absence of Dppa2 (Fig. S5F). Whilst this did not reach significance, this likely reflects an incompletely penetrant memory at the single cell level, similar to Esg1." The changes were minimal and non-significant. Thus, the data say that there is a difference between these genes relative to Dppa2 and that Dppa2 acts as an epigenome 'surveyor' only for two of the analyzed genes. The authors should have investigated why there is such difference.

R1: We have addressed this issue in three separate ways, which collectively show that the absence of *Dppa2* permits epigenetic memory at least at a significant cohort of sensitive genes.

1.) First, taking a candidate approach, we targeted epialleles to three additional loci and overall find silencing inheritance at *Smoc1*, *Jade1* and *Esg1* specifically in *Dppa2* knockout. At many other targeted loci, their expression trends towards maintaining a memory of the prior repressed state in the absence of *Dppa2,* for example *Adamts7* (*p=0.07*) and *Pten* (*p=0.14*) exhibit 30-50% repression maintenance after DOX-withdrawal. Given that the loci for which we have single-cell resolution (e.g. *Esg1*) exhibit a bimodal distribution of memory (i.e. probabilistic reversion), and we necessarily assay other loci at population level, the majority of any memory effect may be masked by a proportion of cells in the population reverting to an active expression state. Indeed at *Esg1*, 59% of cells maintain silencing at the same stage, which corresponds to only a slightly greater than 50% repression at the population level.

   In our previous draft we explicitly state of additional genes that "*Whilst this* <u>*did not reach significance,*</u> *this likely reflects an incompletely penetrant memory at the single cell level, similar to Esg1*". We have now modified this to be clearer and softer stating "*a trend of memory was propagated at Pten, Cdh1, Greb1 and Adamts7 specifically in the absence of Dppa2 (Fig EV4G). Whilst these did not reach significance, this could potentially reflect an incompletely penetrant memory (bimodality) at the single cell level similar to Esg1, which we cannot resolve at the population level by qRT-PCR"*. Overall this data supports the conclusion that programmed epialleles can be inherited at least at a subset of loci (*e.g. Esg1, Smoc1, Jade1*) only in absence of *Dppa2* and, albeit insignificant, that there is a trend at the population level at other examined loci. In contrast, all of these loci revert epigenetic silencing in WT cells, underlining the principle finding that *Dppa2* is part of the mechanism that blocks epiallelic memory in naïve ESC.

2.) Because the strategy of programming epialleles one gene at a time and tracking memory is inefficient, laborious, and essentially a fishing exercise, we reasoned that a larger unbiased approach of programming epigenetic silencing at many loci would be more powerful to address the important reviewer concern. We therefore undertook a major line of experiments to epigenetically silence hundreds of genes in parallel using a polymorphic (BL6/Cast) female ESC line harbouring an inducible endogenous *Xist (*Schulz et al, 2014). Addition of DOX drives epigenetic repression of all active X-linked genes on the BL6 allele, via recruiting the heterochromatic silencing machinery (new *Fig 3A*).

   This experiment revealed two clear results. Firstly, upon DOX-withdrawal of *Xist*-mediated epigenetic silencing 92% genes *rapidly* revert to their prior active state in <u>*wildtype*</u> naïve ESC. Such reversion is in contrast to differentiated cells, whereby epigenetic memory of *Xist*-mediated silencing is faithfully propagated through many mitosis at essentially all loci. This further supports our key observation that naïve pluripotency carries the specific property of blocking epigenetic memory of heterochromatic silencing (new *Fig 3B-C*). Secondly and more pertinently, we then generated new and independent *Dppa2* knockout lines in this inducible-*Xist* background and found that upon DOX-withdrawal here, there is now a <u>significant (*p=0.04*) block in gene reactivation in naïve ESC when *Dppa2* is absent</u> (new *Fig 5F*). In other words, an epigenetic memory effect. Furthermore, clustering identified a key group of genes that propagate complete memory of prior epigenetic silencing in the absence of *Dppa2* but not in WT ESC (new *Fig 5G-H*). Overall, we used an orthologous approach based on inducing epigenetic silencing at hundreds of genes to demonstrate that, unlike WT, *Dppa2* KO ESC can propagate epigenetic inheritance at a subset, but not all, targeted loci.

3.) Finally, we take on board the reviewer's broader point that some aspects were worded to strongly and have softened our conclusions throughout. In particular by emphasising that *Dppa2* is only part of a wider regulatory structure that restricts epigenetic inheritance in naïve ESC, and that only a subset of genes are sensitive to *Dppa2* loss. We do also feel its important to point out that the observation at a subset of loci is an important finding. It reveals the principle that epigenetic inheritance uncoupled from underlying DNA sequence is unlocked specifically by abrogating *Dppa2*.

C2: Next, the authors asked whether the reversal of ectopic silencing at endogenous loci was a feature of naïve ESCs. They analyzed p53 in differentiated cells (i.e. upon endoderm induction) and found that ectopic silencing of p53 could be maintained, in particular DNA methylation whereas H3K9me3 could not. The authors also found that p53 silencing of ectoderm cells represent a growth advantage that enables the propagation of ectopic p53 silencing within the population. The authors stated that "These data imply that differentiated cells, but not naïve pluripotent ESC, are competent for epigenetic inheritance of ectopic heterochromatin". However, the authors did not take into consideration the important point that naïve ESC that are cultured in 2i have low DNA methylation activity since the de novo DNMT3A and 3B are downregulated. Consequently, with the exception of the imprinted genes, the genome of naïve ESCs is hypomethylated. Thus, the difference in the epigenetic memory observed between naïve ESCs (treated with 2i) and differentiated cells is very luckily due to the differential DNA methylation activities. This point should have been discussed. Further, the authors should have tested whether ESCs cultured in serum, which have a fully functional DNA methylation machinery, can or cannot reverse ectopic silencing at endogenous loci.

R2: The reviewer notes that we observe a fundamental difference in epiallele inheritance between naïve pluripotent cells and differentiated cells (endoderm) (Fig 6). Further we find multiple tested lineages *and* primed pluripotent cells also propagate epigenetic silencing (Fig EV5A-B), implying naïve pluripotency has a unique functional capacity to erase programmed epialleles. The reviewer suggests that the globally hypomethylated state of naïve ESC, as previously reported by us and others (Ficz *et al*, 2013; Habibi *et al*, 2013; Hackett *et al*, 2013; Leitch *et al*, 2013), may underlie this difference. Indeed, we discussed this in the introduction by hypothesising that global demethylation may be preferentially functionally linked with restricting epigenetic memory rather than emergence of pluripotency (see lines 82-87)

To directly address the functional significance of global hypomethylation relative to naïve status further, we have repeated our experiments in the context of serum/Lif (S/L) ESC, as suggested by the reviewer. ESC in S/L are predominantly in the naïve state of pluripotency yet acquire high global DNA methylation enabling the relative contribution of naïve status vs DNA hypomethylation to be parsed. We found that programmed heterochromatin is readily reversed in serum/LIF ESC, albeit with slightly slower dynamics than ESC in 2i/L (*new* Fig 6G). In contrast differentiated cells exhibit silencing memory. The slower dynamics in S/L relative to 2i/L could indicate a contributory, but non-essential role of global DNA hypomethylation for erasure, or alternatively may reflect the metastable status of ESC in S/L, with some subpopulations not in 'naïve' status. In any case, these data argue that global hypomethylation *per se* is not requisite, and that additional properties of naïve cells underlie their unique capacity to reset acquired heterochromatin states (including *Dppa2* activity).

C3: They also tested other genes and stated that "We observed that most regions reverted, albeit at least two (Greb1 and Jade1) exhibited robust inheritance of a prior silenced state in wildtype endoderm". This is an unclear statement. They tested 5 non-imprinted genes and only 2 of them exhibited robust inheritance of ectopic silencing, this is not "most of the genes".

R3: As the per the quote we state that, *"We observed that most regions reverted, albeit at least two (Greb1 and Jade1) exhibited robust inheritance.* We are explicitly pointing out that "most" genes reverted (3/5) and therefore not claiming the majority show inheritance. We have however now re-phrased the sentence for clarity to read:

> *"We found that Jade1 and Greb1 exhibit robust inheritance (p<0.05) of a prior silenced state specifically in endoderm (Fig EV5), whereas Cdh, Adamts7 and Pten reinstate their original activity, implying a degree of context-dependency".*

We believe that this emphasises fairly that both some loci can propagate memory in specifically differentiated cells (i.e. not in ESC), and also that some show a trend towards reversion, suggesting a number of regulatory factors are at play.

C4: The sentence concluding these results is very unclear and reflects the fact that the authors were not able to dissect the mechanisms of this reversal or inheritance of ectopic silencing: "Taken together, these data suggest that the potential for epiallele inheritance of de novo heterochromatin is influenced by multiple cell-type- and genomic- context dependent factors. In the case of p53, augmenting weak-acting heterochromatin inheritance in differentiated [cells] with a favourable advantage changes the balance of dynamic forces to enable propagation of ectopic chromatin states within the population." What about Greb1 and Jade1 in differentiated cells? Do they have also a growth advantage? Does Dppa2 affect the silencing inheritance at p53 in ESCs?

R4: We find that programmed epigenetic silencing of *p53* is inherited in differentiated cells both *in vitro* and, amongst a significant fraction of cells, *in vivo.* We also find silencing of *p53* confers a selective growth advantage which contributes to enriching cells carrying epigenetic memory of silencing in the population. Finally, we find that each locus responds differently; some exhibit varying degrees of programmed epigenetic memory others not (see R1 & R3). Thus, we believe the sentence "*these data suggest that the potential for epiallele inheritance of de novo heterochromatin is influenced by multiple cell-type- and genomic- context dependent factors…*" fairly summarises the extensive data we present. In particular we think that the potential for epigenetic memory is influenced by a complex interplay between cell identity and genomic sequence that is beyond the scope of this manuscript to fully dissect. In response to the reviewer, we have re-written parts of the manuscript to emphasise the influence of the selective advantage on *p53* silencing is <u>not</u> the only factor that enables propagation in differentiated cells (e.g. at *Greb1* and *Jade1*). We have also more clearly spelled out that other contributory mechanisms beyond DPPA2 are likely involved in reversion since not all loci maintain memory during endoderm differentiation or in *Dppa2* knockout ESC, including *p53.* Importantly however, our study does provide novel proof of principle of epigenetic inheritance *in vivo*, at an endogenous locus (*p53*), that is partly facilitated by a selective advantage, and also shows that this can be restricted by naïve pluripotency. We believe this is an important observation.

C5: In summary, this work showed a nice dox inducible method to establish silencing at defined endogenous genomic loci, however, this methodology already existed.

R5: We would address this reviewer concern with two lines of response.

1.) First, iCRUSH demonstrably *does* represent an advance on previous approaches in several important outcomes (i) iCRUSH generates large *domains* (>10kb) of modified chromatin, rather than just a few nucleosomes (<1kb), as with direct KRAB-fusions that are generally employed (ii) iCRUSH significantly enhances ON-target efficacy through combining several extant improvements, including an enhanced gRNA scaffold and a modular recruitment strategy, thereby depositing greater modification levels than published KRAB systems. This *recapitulates physiologically relevant levels* of modification that are necessary to draw conclusions on functionality (iii) Our system is engineered with d2 domains to be *destabilised* to enable memory to be rapidly assessed without confounding re-targeting. (iv) Both effector and gRNA are fluorescently labelled (sfGFP and BFP) to enable convenient tracking, whilst all components have been sub-cloned into an inducible piggyBac system to enable ease of use for other interested researchers. Thus, iCRUSH combines several advances for the emerging epigenetic editing field, which we fully characterise and validate.

2.) Second, iCRUSH is the *tool* that we use to ask *biological* questions. Even should we hold the view that iCRUSH does not provide any 'advance', it is still the best approach available to investigate whether precisely targeted heterochromatin states can confer 'memory'. For this reason, we actually did not claim iCRUSH as a novel part of the manuscript. Indeed, it is not even mentioned in the abstract. Rather it is used as a convenient acronym to describe a tool that we developed, which is fully referenced throughout, and which is a means to an end.

C6: Further, they show the reversal of ectopic silencing, however, also here nothing new.

R6: The generally accepted paradigm in the field is that heterochromatin forms a *heritable* self-reinforcing network via well-characterised read-write mechanisms, as demonstrated in numerous studies across model organisms and as fully referenced in the introduction. We generate robust domains of H3K9me3, H4K20me3 and DNA methylation (heterochromatin) at multiple loci and used an orthologous approach to silence the whole X-chromosome, and show that, contrary to differentiated cells where the paradigm holds, naïve pluripotent cells rapidly reverse this. We are unaware of any citable literature showing that naïve pluripotency is a specific blockade to heritable heterochromatin epialleles on this scale. Likewise, we are unaware of literature showing that such targeted epialleles can be inherited *in vivo* in mammals.

C7: Although the authors showed that Dppa2 is implicated in the reversal of ectopic silencing for Esg1 reporter and Jade1 in naïve ESCs, this seems to be a gene specific effect since the other genes are not affected by the lack of Dppa2.

R7: We have now made it clearer that there is genomic context-dependency in the role of *Dppa2 (*or *"gene-specific effects")* throughout the manuscript. Moreover, as detailed above (R1) we have supplemented the original observations with additional programmed epialleles and an X-inactivation paradigm demonstrating a significant role for *Dppa2* at multiple loci. That DPPA2 is not involved at every locus does not negate the clear and important observation that it has a critical role in preventing epigenetic memory at a key subset of other genes, that it was the top hit in a genome-wide genetic screen, and that it has been comprehensively validated.

C8: The authors failed to provide mechanistic insights of this process and did not investigate the role of DNA methylation that seems to be key in the studied system. The experiments and the methodology used are exceptionally well done. However, the biological readout of these experiments is unclear.

R8: As detailed in R2, we have now added additional experiments indicating that global DNA hypermethylation may play a supportive role, but is not by itself the key driver of epiallele memory . We attempted to supplement this by using DNA methylation inhibitor (5-Aza-dC) that impairs genome wide DNA methylation (Appended figure 1.1A) to revert epigenetic memory. We found that 5-Aza-dC did erase DNA methylation specifically at *Esg1* promoter in *Dppa2* KO ESC (Appended Fig 1.1B) and concomitantly reduces the percentages of memory cells (Appended Fig 1.1C). On the other hand, 5-Aza-dC also promoted increased expression in wild-type cells that exhibited no memory, without changes in DNA methylation, which was already very low (<5%). Therefore, we believe that 5-aza-dC is exerting these effects through its well-characterised indirect effects, but nevertheless provide the data here for reviewer scrutiny. Rather than solely DNA methylation, we postulate that the continued absence of H3K4me3, as well as a multitude of other potential epigenetic modifications yet to be charted in our system, together with DNA methylation, may cooperatively perpetuate epigenetic silencing at programmed loci. We discuss this in the conclusions.

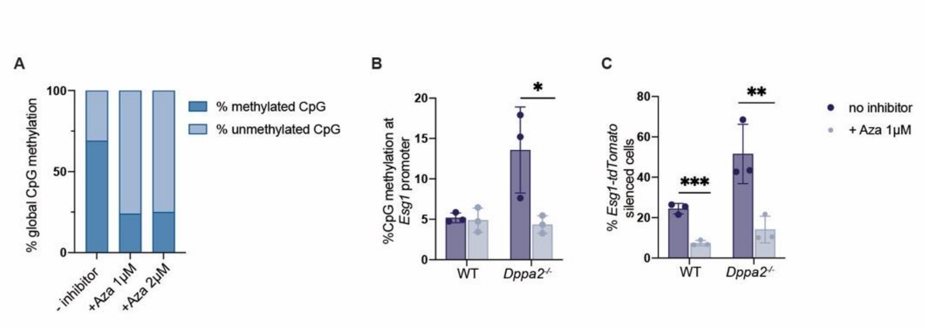

Figure 1.1 Role of DNA methylation in epigenetic memory at Esg1. (A) Percentages of genome-wide DNA methylation by LUMA assay with or without 5-Aza-dC treatment (two different concentrations). (B) Percentage of CpG methylation detected by pyrosequencing at the Esg1 promoter in WT or Dppa2$^{-/-}$ cells with or without 5-Aza-dC. (C) Percentage of Esg1-tdTomato silenced cells detected by flow cytometry in WT

or Dppa2$^{-/-}$ cells with or without 5-Aza-dC. Asterisks indicate p-values by unpaired t-test; *p<0.05, **p<0.01 ***p<0.001. Error bars ± S.D.

Referee #2

Carlini and colleagues established a cell line carrying an inducible dcas9 to target KRAB or DNMT3A/L (or combination) to a reporter Esg1 allele. They track transcriptional dynamics using this reporter to show that silencing is efficient when the system is induced by DOX after 7 days, and covers around 10kb from the sgRNA target site. They then do 'wash-off' experiments whereby they remove Dox to probe potentially inheritability of the silenced state. Using this approach they show that silencing is practically reverted 7 days later. Using a Crispr screening, they show that this reversal is mitigated in the absence of Dppa2. They then turn to another reporter, p53, and explore whether the silenced status of the p53 reporter is maintained during differentiation.

The manuscript deals with a very important topic in the epigenetics field, inheritability of transcriptional states, which is largely unexplored in mammals. This is therefore a very welcome effort, with a well-designed experimental set-up to address it. In general, I find the manuscript well executed and with appropriate controls, but somehow overstated in terms of the universality of their conclusions. I would recommend the manuscript for publication at EMBO Journal, provided that the following points are addressed by the authors (please next time include page numbers!):

We thank the reviewer for their thorough and balanced critique of our study, and for the time that this takes. We are also glad to hear that they find the manuscript '*deals with a very important topic in the epigenetics field'*, that it is *'well executed and with appropriate controls'* and that they *recommend the manuscript for publication at EMBO Journal'* following appropriate revisions. We also apologise for the lack of page numbers, and do appreciate how irritating this can be. Please find below our detailed responses (R) to the reviewer's comments (C).

C1: Fig. 4F. There does not seem to be significant changes in promoter accessibility beyond Esg1. However, Wolf Reik group has documented significant changes in gene expression in Dppa2-depleted cells by siRNA (including e.g. 2C genes; Genes Dev 2019) - and therefore I find the ATACseq analysis in Fig. 4F at odds with known expression changes upon Dppa2 loss. The authors should perform a differential accessibility/peaks analysis and provide information on additional loci, beyond Esg1, that change accessibility in Dppa2-/- cells.

R1: As the reviewer points out, removing *Dppa2* leads to expression changes at a subset of genes, as reported by Reik and colleagues, and our own lab (Eckersley-Maslin *et al*, 2020; Gretarsson & Hackett, 2020). Our data as presented in Fig 4F is consistent with this, in that a subset of loci are affected in *Dppa2* knockout, albeit this was unclear as they were not highlighted. To remedy this, we re-plotted the genome accessibility from the same ATAC-seq data as in Figure 4F, highlighting all significant differential promoters (appended Fig 2.1.A). We have specifically marked the top differential genes reported by Reik and colleagues from their 2019 paper (Eckersley-Maslin *et al*, 2019), to demonstrate the overlap of our data.

Notably however, our cells were maintained in 2i/L whereas Reik and colleagues studies utilised culture in serum/LIF. Under these latter conditions 2C-associated genes are active in a subpopulation of wildtype cells and exhibit strong differential expression profiles upon *Dppa2* knockout. In contrast, 2C genes are expressed at lower baseline levels in 2i/L, from a very small subpopulation. As such, 2C genes promoters exhibit very low initial accessibility in WT ESC at the population level when maintained in 2i/L, and thus could not lose further accessibility upon *Dppa2* KO in our system (see appended Fig 2.1.B). Indeed, recent data has indicated *Dppa2* does not influence the 2C state *in vivo* (Chen *et al*, 2021), which more closely models 2i/L than serum/LIF conditions, and is consistent with our data. Overall, even accounting for different culture systems, we observe good concordance between our accessibility data and published expression data, with the exception of 2C-genes as detailed above.

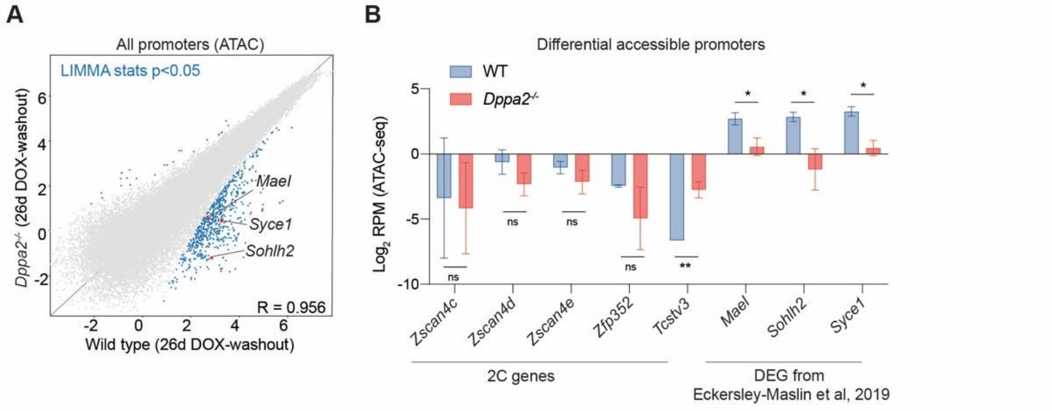

*Figure 2.1. Comparison of differential accessible promoters in Dppa2$^{-/-}$ with differentially expressed genes from Eckersley-Maslin et al, 2019. (A) Scatterplot showing genome accessibility across all promoters after 26d DOX-withdrawal in WT versus Dppa2$^{-/-}$ cells. Highlighted in blue are differentially accessible promoters determined by LIMMA stats ($p<0.05$) in two independent clonal lines. (B) Log2 RPM of genome accessibility at the indicated gene's promoters (2C genes or top differentially expressed genes (DEG) from Eckersley-Maslin et al, 2019). It shows that 2C genes exhibit very low genome accessibility in WT cells cultured in 2i/L, while significant differential accessibility is detected in our Dppa2$^{-/-}$ data at top DEGs. Asterisks indicate p-values by unpaired t-test; *$p<0.05$, **$p<0.01$. Error bars ± S.D.*

We are sensitive to the fact that the original phrasing of our result was inaccurate by implying no or minimal changes in initial accessibility status of *Dppa2* KO, for which we apologise. Therefore, in addition to intersecting data, we have re-phrased the text to make this distinction clearer. We also further consider the difference between the (limited) initial changes in *Dppa2* KO and the additional cohort of loci that become predisposed to inherit stochastic or programmed epialleles in the discussion (lines 449-456).

C2: Along the same lines, how 'general/targeted' are the observations in the Dppa2 mutants, beyond the targeted iCRUSH system (e.g. beyond the reporter) in +Dox and after washout conditions. An analysis to address this is essential to distinguishing direct effects of KRAB targeting versus general changes in e.g. expression, K9me3 or accessibility due to Dppa2 loss overall. The authors present scRNAseq, but limit this to the tagged Esg1 and p53 alleles. What happens genome-wide? (and to the wild-type, non-targeted allele?, which in fact could be used as control).

R2: The transcriptional and epigenetic changes at baseline level of *Dppa2* knockout have been charted by us and others previously (Eckersley-Maslin *et al.*, 2020; Gretarsson & Hackett, 2020), and show no alterations to *global* chromatin or DNA methylation, but that a small number of developmental-associated genes (and LINE) become epigenetically silenced. To address this further we have used accessibility profiling here (ATAC-seq) and observed an overlapping subset lose accessibility in *Dppa2* KO (see appended *Figure 2.1.A*). Furthermore, data previously generated in the lab and published in (Gretarsson & Hackett, 2020) identified only 269 differentially expressed genes in *Dppa2*$^{-/-}$ cultured in 2iL. Collectively this suggests that *Dppa2* deletion by itself does not induce global or major 'general' changes in naïve ESC but rather results in a fraction of developmentally-associated genes becoming epigenetically repressed. Thus, <u>the majority of genes remain in their erstwhile epigenetic state</u>. Crucially, here we show many of these apparently unaffected genes are now sensitised to a stochastic or 'programmed' epigenetic perturbation (e.g. *Esg1, Jade1, Smoc1,* examined *X-linked loci*), and cannot revert to original status without DPPA2. This is specific to events after acquiring an ectopic (programmed) epigenetic state, rather than any pre-existing defect. We have included discussion in the conclusion to highlight the distinction between prior changes and the additional 'sensitized' genome in absence of *Dppa2*.

For the use of the second allele as a control as the reviewer suggested, with our strategy we have generated a hemizygous knock-in reporter allele to enable dynamic and quantitative tracking over time at endogenous loci. To analyse these, we target heterochromatin to both alleles of *Esg1* and *p53*, respectively, since we cannot distinguish them at the 5' end (endogenous promoter). Therefore we can not use the second allele as

a control since both alleles are necessarily targeted. Our single-cell analysis in this context is quantitative flow cytometry of the endogenous knock-in reporter, and we thus cannot extend genome-wide.

C3: What is the sgRNA used in Fig. 1B/C, and throughout? This is nowhere stated in the text. The TSS at +87bp shows clearly a repressive effect regardless of KRAB (fig. 1G) and therefore the distinction of which sgRNA is used throughout the manuscript is fundamental to the conclusions of the paper.

R3: We apologise for not having specified which gRNA we used in the text and have now changed this in text (see line 106, throughout figure legends, and in the figure (Fig 2A). Specifically, for *Esg1* we used gRNA#1 (+87bp, adjacent to TSS) throughout the experiments, since it shows a more penetrant effect of initial silencing, facilitating a greater dynamic range to track recovery/epigenetic memory. Whilst this gRNA exhibits a mild repression upon GFP-recruitment (through steric hindrance), *importantly* this does not affect the epigenetic state of the locus (Fig EV2E) *nor* show epigenetic memory (Fig 2B-C-E). Rather, epigenetic memory is only observed upon programmed heterochromatin (KRAB) targeting (in *Dppa2* knockout). Moreover, we obtained highly comparable results with gRNA#2 (memory for KRAB but not GFP), for which there is no steric hinderance effect.

In order to further rule out the effect of steric hindrance on epigenetic memory we compared the two conditions (KRAB and GFP control) over closer time-points. We induced equivalent silencing by transient transfection of gRNA via either epigenetic (*KRAB$^{-GFP-scFv}$*) or non-epigenetic (*GFP^{-scFv}*) programming for 3 days. Cells that acquired quantitatively equal repression (TOM-) were flow sorted and cultured in the absence of doxycycline, to release the editing system (*appended Fig. 2.2.A*). Epigenetic memory was then assayed in the GFP-/BFP- fraction of the population (the one that switch off the editing system) by measuring the percentage of *Esg1-tdTomato* negative cells by flow cytometry (*appended Fig 2.2.B*). We observed that silencing is lost much faster in the *GFP^{-scFv}* control compared to epigenetic-induced repressed cells (*appended figure 2.2.B*). Importantly replication time was comparable in the two samples, excluding any confounding effect from replication dependent dilution of the marks (Appended Fig. 2.2.C). Therefore, this support that steric hindrance (as induced by GFP control) does not account for epigenetic memory in KRAB targeting.

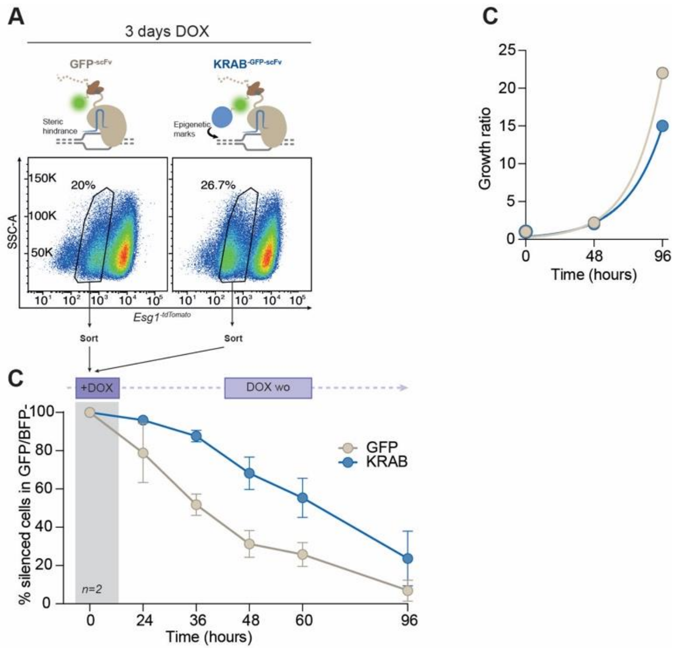

Figure 2.2. KRAB Induced heterochromatin but not GFP targeting mediates short term memory. (A) Density plots shows the intensity of Esg1$^{-dTomato}$ reporter plotted over side scatter area (SSC-A) by flow cytometry. Black boxes indicate the gates used to sort the cells with equal tdTomato repression after 3 days of DOX and subsequently used for the experiment (panel c). (B) Time-curse of the % of silenced cells out of the GFP/BFP- population at 12 hours intervals up to 96 hours of DOX washout. Error is measured as standard deviation between two biological replicates. (C) Exponential curve shows growth obtained by measuring the

ratio between the cell count at each timepoint over the initial number of cells at time 0. The difference observed in the growth between the two conditions does not account for the difference in epigenetic memory.

C4: The authors culture ESCs in 2i (they actually say 1%FCS in the methods, is this a mistake?), which are DNA hypomethylated and express high levels of TET. This could in fact mechanistically explain the reversion of the silenced state. How would Fig. 1G and 2B look-like if the cells are grown in serum/LIF without 2i? (and ideally also K9me3 levels as in Fig. 1B?)

R4: We performed our analysis in 2i/L culture conditions, since it more closely recapitulates the epigenetic/transcriptional state *in vivo* that we are modelling than does serum/LIF conditions (Habibi *et al.*, 2013; Hackett & Surani, 2014; Marks *et al*, 2012). Nevertheless, the reviewer is absolutely correct that by using ESC in serum/LIF (S/L) we can separate the role of naive pluripotency from DNA hypomethylation, and understand the relative impact of the latter on propagation of epigenetic memory. We have therefore performed this experiment and observed the following. Programmed heterochromatin is readily reversed in S/L ESC, albeit with slower dynamics than ESC in 2i/L (see new Fig 6G). The slower dynamics in S/L relative to 2i/L could indicate a contributory, but non-essential, role of global DNA hypomethylation for erasure, or alternatively may reflect the metastable status of ESC in S/L, with some subpopulations not in 'naïve' status. In any case, these data argue that global hypomethylation *per se* is <u>not</u> requisite for reversion, and that additional properties of naïve cells (ESC in 2i/L and most in S/L are naïve) underlie their unique capacity to reset acquired heterochromatin states. Moreover, we find primed cells (EpiLC), can propagate memory (new Fig EV5B), further supporting the specific role of naive status.

C5: The authors data suggest that DNA methylation may be responsible to lock a silenced state. Would the dynamics of Esg1/tdTomato expression upon removal of Dox change in TKO cells? (Dnmt1,3a and 3b KO)

R5: As the reviewer points out, it would be interesting to further dissect the role of DNA methylation in locking the silenced state once the initial trigger is released. However, by performing it in constitutive TKO cells the initial silencing with iCRUSH would be impacted, confounding relative comparisons to WT and reducing the dynamic range for epigenetic memory. Moreover, TKO cells cannot be differentiated since they undergo apoptosis upon lineage-commitment. Therefore, to address this point without affecting the initial silencing, we repeated the memory experiment in *Dppa2* KO and WT ESC, in presence of an inhibitor of DNA methylation (5-Aza-dC). Whilst we found that 5-Aza-dC did erase DNA methylation specifically at *Esg1* promoter in *Dppa2* KO ESC (Appended Fig 1.1B) and concomitantly reduces the percentages of memory cells (*appended Fig 1.1C*), it also promoted increased expression in wild-type cells that exhibited no memory, without changes in DNA methylation, that was already significantly low (<5%). (*appended Fig. 1.1.A-C*). Therefore, we believe that 5-aza-dC is exerting these effects through its well-characterised indirect effects on genome responses (e.g. see (Roulois *et al*, 2015) and consequently are uncomfortable drawing conclusions. Importantly the results from S/L experiments provide evidence that high global levels of DNA methylation *per se* at not sufficient to propagate memory. Rather than solely DNA methylation, we therefore postulate that the continued absence of H3K4me3, as well as a multitude of other potential epigenetic modifications yet to be charted in our system, together with DNA methylation, may cooperatively perpetuate epigenetic silencing at programmed loci.

C6: Since the ChIP data for Dppa2 is available (Eckersley-Maslin et al., NSMB 2020) it would be interesting to check whether Dppa2 actually binds to Esg1 and the other loci studied in this manuscript. That may give clues to understand the differences between 'classical' and imprinted loci.

R6: To address this point, we have performed a new and extensive CUT&RUN-seq analysis of DPPA2 binding in our cells, with excellent enrichments that overlap Eckersley-Maslin et al 2020. We find DPPA2 binds to the majority of all transcriptional start sites genome-wide in ESC irrespective of their activity status, and essentially all CpG island promoters, including all targets we tested (see new Fig EV4H-J) . This is consistent with the idea that DPPA2 samples most promoters as a potential 'surveyor' that contributes to

restricting aberrant or ectopic epigenetic memory. At many loci loss of DPPA2 binding is sufficient to sensitise the promoter to heritable silencing memory, whilst at a major fraction of other promoters, memory is still reverted in *Dppa2* knockout, indicating redundant erasure systems. Thus, DPPA2 binds to the majority of promoters as a default surveyor and therefore its binding does not distinguish genes that exhibit memory (e.g. *Esg1*, *Jade1, Smoc1* etc) from those that do not, upon *Dppa2* knockout. We propose the distinguishing features are likely complex and include redundant erasure systems, as well as inherent chromatin and DNA sequence features of each locus.

C7: Why did the authors not confirm the effect of Dppa2 on P53 loss of repression? All other genes presented in figure 2 are examined, but not p53. Also, they state that 'we observed propagation of repression at Pten, Cdh1, Greb1 and Adamts7'. However, the data in Fig S5F do not support that statement, as only Jade1 is statistically different. I agree with their conclusion that it is likely to be context-dependent but Dppa2 seems to be important for only 2 genes out of the 9 tested and this should be clearly spelled-out.

R7: The reviewer is correct in that, owing to the challenges in tracking dynamic epigenetic states programmed at specific loci in living cells we have an incomplete understanding of how *Dppa2* affects different loci. To address this issue we have taken three separate approaches, which include a major new line of unbiased experiments across hundreds of loci, and that collectively show that the absence of *Dppa2* permits epigenetic memory at least at a significant cohort of sensitive genes. We have also spelled-out more clearly that not all loci are affected by *Dppa2* loss, including *p53*.

1.) First, we targeted heterochromatin epialleles to three additional loci and overall, combined with previous data, find silencing inheritance at *Smoc1*, *Jade1* and *Esg1* specifically in *Dppa2* knockout. At many other targeted loci, the activity trends towards maintaining a memory of the prior repressed state in *Dppa2*-null*,* but as the reviewer points out and we clearly state in the text, this does not reach significance. For example, *Adamts7* (*p=0.07*) and *Pten* (*p=0.14*) exhibit 30-50% repression maintenance after DOX-withdrawal. Notably, given that the loci for which we have single-cell resolution (e.g. *Esg1*) exhibit a bimodal distribution of memory in *Dppa2* knockout, and we necessarily assay other loci at population level, the majority of any memory effect is likely masked by the many cells in the population cells that revert to an active expression state. Indeed, at *Esg1*, 59% of cells maintain silencing at the same stage, which corresponds to only a slightly greater than 50% repression at population level. Overall this data supports the principle that programmed epialleles can be inherited at least at a subset of loci (*e.g. Esg1, Smoc1, Jade1*) only in absence of *Dppa2,* and that there is a trend, albeit insignificant at population level, at other examined loci. In contrast, all of these loci revert in WT cells, underlining the principle that *Dppa2* is part of the mechanism that blocks epiallelic memory naïve ESC, which we trace at single-cell resolution.

2.) Because the strategy of programming epialleles one gene at a time and tracking memory is inefficient, laborious, and essentially a fishing exercise, we reasoned that a larger unbiased approach of programming epigenetic silencing at many loci would be more powerful to address the important reviewer concern. We therefore undertook a major line of experiments to epigenetically silence hundreds of (X-linked) genes in parallel using a polymorphic (BL6/Cast) female ESC line harbouring an inducible endogenous *Xist.* Addition of DOX drives epigenetic repression of all active X-linked genes on the BL6 allele, via recruiting the heterochromatic silencing machinery (new Fig 3A).

   This experiment revealed two clear results. Firstly, upon DOX-withdrawal of *Xist*-mediated epigenetic silencing in naïve ESC, 93% genes rapidly revert to their prior active state in naïve ESC. Such reversion is in contrast to differentiated cells, whereby epigenetic memory of *Xist*-mediated silencing is faithfully propagated through many mitosis (Loda & Heard, 2019). This further supports our key observation that naïve pluripotency carries the specific property of blocking epigenetic memory of heterochromatic silencing (new Fig 3B-C). Secondly and more pertinently, we then generated new independent *Dppa2* knockout lines in this inducible-*Xist* background and found that upon DOX-withdrawal here, there is now a significant (*p=0.04*) block in gene reactivation in naïve

ESC (new Fig 5F). Furthermore, clustering identified a major group of genes that propagate memory of prior epigenetic silencing in the absence of *Dppa2* but not in WT ESC (new Fig 5G). Overall, we used an orthologous approach based on inducing epigenetic silencing at hundreds of genes to demonstrate that, unlike WT, *Dppa2* KO ESC can propagate epigenetic inheritance at a subset, but not all, targeted loci.

3.) Finally, we take on board both reviewers broader point that some aspects were worded too strongly and have softened our conclusions throughout. In particular by emphasising that *Dppa2* is only part of a wider mechanistic structure that restricts epigenetic in naïve ESC, and that only a subset of loci are sensitive to *Dppa2* loss. We do also feel its important to point out that the observation at a subset of loci is an important finding. It reveals the principle that epigenetic inheritance uncoupled from underlying DNA sequence is unlocked specifically by abrogating *Dppa2*.

C8: When looking at Fig 6D it seems that the 7% actually comes from an outlier, most of the other embryos retain only 0-0.5% tomato-negative cells. The data presented in Fig 6C is then not reflecting the actual data.

R8: To test *in vivo* memory we generated 15 independent embryos harbouring cells with 'prior' epigenetic silencing of *p53*, or controls with no prior silencing, out of two independent experiments. In all controls *p53* was active in essentially all cells above background. In contrast, embryos with a prior silenced allele exhibited a spectrum of memory ranging from complete reversion to up to 7% of all embryonic cells propagating silencing. In Fig 6C we plot the example of highest memory, as proof of principle of what is possible given there are, to our knowledge, no previous reports memory of a programmed epiallele *in vivo* in mammals. To ensure this is representative and reflects the actual data, in Fig 6D we used a dotplot showing *every* datapoint from each embryo revealing the full distribution of data. To be absolutely clear of no mis-representation, we further use a heatmap in Fig 6E showing the percentage memory in each individual embryo. We do not think we can be more explicit about the full range of results we obtained. In the text we state that we observed "*up to 7% of cells of foetal cells inherited epigenetic silencing memory*". Finally, we note that as a group of all embryos, memory of prior *p53* silencing is maintained with statistical significance (p=0.03), albeit clearly with a large variance. Thus, whilst we fully agree that there is extensive reversion of prior silencing *in vivo*, and in some embryos this is complete, we also discover the principle that at least a subset of cells can retain silencing memory. This has implications for our understanding of epialleles, and their potential functional significance (see R9 below)

C9: Along the same lines, Fig. 6E would suggest that in contrast to ESCs-differentiation in culture, in the embryo the cells do not maintain transcriptional repression (only 50% of the embryos have a phenotype). While I find the potential of some clonal inheritance might be interesting, this is really only limited to 7% of the cells (or perhaps even less). The discussion of the authors is therefore not balanced.

R9: We agree that only a relatively small percentage of cells appear to propagate p53 silencing in wild-type contexts (maximum 7%), and in some cases none at all. However, considering the crucial role of *p53* as a tumor-suppressor gene, silencing of *p53* at even a small percentage of cells could have a major effect on disease susceptibility and therefore is potentially highly significant. Indeed, 7% of cells translates into an absolute number on the order of 10 billion (10^{10}) cells in a mature adult mouse that would have attenuated *p53*. Given, abrogation of *p53* predisposes to almost certain early-onset cancer, impaired p53 in 10^{10} organismal cells would be predicted to lead to unfavourable outcomes (we cannot perform this experiment for ethical and timeframe reasons). Thus at least in the case of *p53*, we believe our observations could be demonstrative of a significant effect. We discussed this in the conclusions stating:

> *Importantly, we demonstrate this effect* [p53 epigenetic inheritance] *in vivo in mice, with up to 7% of cells within a whole embryo heritably maintaining the legacy of prior p53 silencing. This is relevant as even a small fraction of organismal cells corresponds to a large absolute number that carry*

epigenetic silencing, and such constitutional epimutations have been linked to cancer risk (Hitchins, 2015; Saunderson et al, 2017)"

To address the reviewers point we have now expanded the discussion stating the cell numbers involved and also making it clear that in our hands, the majority of cells still revert.

C10: I find the manuscript in general overstated regarding the extent of their conclusions and should be toned down. For example, I find the last 10 lines of their introduction overstated, because of the following:
i)the authors do not explore 'normal development';
ii)the authors do not compare e.g. naïve/primed pluripotency, nor they follow their dynamics, to be able to conclude on naïve pluripotency (only because they use 2i for culture)
iii)the conclusion on 'only under selective pressure' is also not justified (see my comments below, regarding p53 role in monolayer differentiation for example)
iv) there is no examination of intergenerational inheritance and/or its propagation in mammals.

R10: We thank the reviewer for this and upon careful consideration of our conclusions agree that we should have been more nuanced. In particular, we have re-phrased our key conclusions around the generality of *Dppa2* to more clearly reflect that whilst it is important to restrict epigenetic memory at some loci, at others it is dispensable. Therefore, *Dppa2* is one (key) part of the systems that operate in naive cells to restrict ectopic silencing memory.

For other specifics, (i) we feel we do explore 'normal development'. Both in our *in vivo* assays (see *p53* results) which are entirely performed in context of normal development and using a developmental model of ESC differentiation, that contrasts with developmentally 'abnormal' cancer lines typically employed for epigenetic editing assays. (ii) We have now added data on 2i/L and S/L conditions. We also use EpiLC as a model of primed pluripotency. (iii) We have scaled back and re-written the conclusions on selective advantage to emphasise this may be specific property pf *p53* (iv) Immediately upon fertilisation, epigenetic reprogramming initiates towards establishing the state of naïve pluripotency. Whilst we do not explicitly examine intergenerational effects, we work on the basis that the transition to naïve pluripotency is the first primary stage that any intergenerational inherited epimutations must pass through to be propagated into the embryo and therefore propose its functionality is, in part, to restrict epimutations.

C11: On page 2 of the results, the authors state that they observed bimodal distribution of Esg1 expression (Fig. 2B) - however, Fig. 2B shows a graded pattern of expression, not bimodal. Please correct (alternative, the authors could make tests for bimodality in their dataset, but the data looks quite clearly graded, not modal). Also, their conclusion on 'probabilistic reversion of epimutations' is, accordingly, not founded, as well as the quite extensive (and unnecessary in my view) discussion emerging from this conclusion.

R11: We acknowledge that the data in WT cells shown in Fig 2B reflects a transient graded distribution during reversion to a fully active state, and we have altered the text to reflect this as suggested (see line 155). Upon *Dppa2* knockout however, we observe a clear bimodal distribution of epigenetic memory. This reflects a probabilistic reversion, as demonstrated by our re-plating experiments (Fig EV4C-E), showing that the chance of remaining silenced rise above 0 with an all or nothing outcome. That memory is not deterministic is informative, we believe, as to the underlying mechanisms at play and for the general principles or how epialleles may propagate.

C12: In the proliferation inhibition experiment, the analysis of expression, DNA methylation etc, should be done at +60 hours, before the cells start to proliferate. Otherwise, the authors cannot conclude safely on the role of proliferation. Endogenous, non-targeted genes/alleles should be used as control here.

R12: The analysis of expression has been performed at +60 hours. We performed analysis at multiple timepoints up to 96hrs, and at no assayed stage found a significant difference between cell cycle inhibited cells and control cells, implying replication-independent activities must be, at least partially, responsible for erasure. Consistently, in our subsequent CRISPR screen we identify *Dppa2* and *Kmt2D* as specific factors

that erase silencing memory independent of proliferation rate, confirming that active mechanisms must be at play. Overall our conclusions here do not rule out a role for proliferation and we state that *"whilst passive dilution may partially contribute to reversion of epigenetic memory, active mechanisms play a key role in erasing de novo epialleles in naïve ESC".*

Minor comments:

MC1: Especially in EV figures some panels have only 1 replicate or information on replicate number is missing. Please state the number of replicates throughout and complete replicate number to >3 where needed.

MR1: We have attempted to show replicates in all panels, including bar chats and boxplots, by plotting the individual data points, which are biological independent rather than technical replicate (e.g. multiple independently generated knockout lines, or independent wildtype clonal lines etc). In some EV panels, some specific samples have <3 replicates since the result is entirely expected, and generally only acts as a reference point for the key experimental panels for which we have 3 replicates. To be clearer on significance of our experiemnts, we have now applied statistical tests throughout and provided more comprehensive information about number of replicates and statistical tests used in the figure legends.

MC2. Please provide additional details for the reporter cell lines used (for example, it is nowhere stated that this is a heterozygous reporter allele? - we are referred to a previous publication by last author, but then I assume the current ms does not harbour the Stella-GFP reporter?).

MR2: We apologise for this omission. The *Esg1* reporter is a heterozygous knock-in allele at the endogenous locus. Similarly the *p53* reporter is also a heterozygous knock-in allele at the endogenous locus. We have now added more details to explain this and ESC derivation in the methods. In addition, there are schematics of reporter design in Fig 1A, 2B and F)

MC3: The authors report that combination of KRAB and DNMT3A/L domains does not enhance reporter repression or maintenance. This contrasts to the report by Nunez et al. (Cell 2021) in differentiated human cells. From this and the analysis of imprinted genes performed by the authors, it seems that DNA methylation is important in that process. The authors should discuss this.

MR3: DNA methylation likely plays an important contributory role in propagating memory in differentiated cells, but its presence is not sufficient to confer memory in naïve ESC (see Fig EV2B-C and new added Fig 6G). In the conclusion we discuss (from line 456) the potential mechanisms at play as suggested

MC4: For the screening, please described how many genes were targeted.

MR4: This has been added to the main text (line 238) and is also stated in the methods.

MC5: Controls for efficient differentiation into the 3 cell types in Fig. S7 should be shown. Along these lines, p53 is known to affect differentiation in monolayer cultures of ESCs (Shigeta 2013). How does the repression of p53 affect the interpretations of chimera contribution and differentiation results in Fig. 6? Please discuss.

MR5: We have analysed key lineage markers of differentiation towards endoderm (*Emb, FoxA1*), ectoderm (*Pax6*, *Sox1*), and primed (*Fgf5, Dnmt3b*) in each of the directed differentiation paradigms, using remaining RNA from the data previously presented. We find these markers are strongly upregulated appropriate to each lineage in *p53* silenced cells (new Fig EV5D). Moreover, *p53* silenced cells efficiently silence naïve markers (*Nanog, Prdm14*) to completion implying full exit from pluripotency. We also note that our *p53* silent cells

were capable of contributing to fully differentiated tissues throughout mid-gestation embryos. Finally, p53 null embryos are fully viable, but susceptible to early-onset cancer, implying the absence of p53 has very little impact on differentiation capacity of cells. Collectively, we believe these suggest that *p53* epigenetic silencing does not have a major impact on differentiation capacity, albeit we do believe it has an impact on cell proliferation rate, as discussed.

MC6: In the abstract, the authors claim that they identified mechanisms of inheritance, but to this reviewer, the authors have not identified a mechanism, they identified molecular players, but not mechanism and I therefore suggest to rephrase.

MR6: We agree that we have identified players involved, and re-phrased the abstract accordingly. Note that we did not claim to have found "mechanisms of inheritance" but rather mechanisms that *restrict* inheritance, which we have now switched to *"factors that restrict inheritance"*.

Chen Z, Xie Z, Zhang Y (2021) DPPA2 and DPPA4 are dispensable for mouse zygotic genome activation and preimplantation development. *bioRxiv*: 2021.2008.2019.457017

Eckersley-Maslin M, Alda-Catalinas C, Blotenburg M, Kreibich E, Krueger C, Reik W (2019) Dppa2 and Dppa4 directly regulate the Dux-driven zygotic transcriptional program. *Genes Dev* 33: 194-208

Eckersley-Maslin MA, Parry A, Blotenburg M, Krueger C, Ito Y, Franklin VNR, Narita M, D'Santos CS, Reik W (2020) Epigenetic priming by Dppa2 and 4 in pluripotency facilitates multi-lineage commitment. *Nat Struct Mol Biol* 27: 696-705

Ficz G, Hore TA, Santos F, Lee HJ, Dean W, Arand J, Krueger F, Oxley D, Paul YL, Walter J *et al* (2013) FGF Signaling Inhibition in ESCs Drives Rapid Genome-wide Demethylation to the Epigenetic Ground State of Pluripotency. *Cell Stem Cell* 13: 351-359

Gretarsson KH, Hackett JA (2020) Dppa2 and Dppa4 counteract de novo methylation to establish a permissive epigenome for development. *Nat Struct Mol Biol* 27: 706-716

Habibi E, Brinkman AB, Arand J, Kroeze LI, Kerstens HH, Matarese F, Lepikhov K, Gut M, Brun-Heath I, Hubner NC *et al* (2013) Whole-genome bisulfite sequencing of two distinct interconvertible DNA methylomes of mouse embryonic stem cells. *Cell Stem Cell* 13: 360-369

Hackett JA, Dietmann S, Murakami K, Down TA, Leitch HG, Surani MA (2013) Synergistic Mechanisms of DNA Demethylation during Transition to Ground-State Pluripotency. *Stem Cell Reports* 1: 518-531

Hackett JA, Surani MA (2014) Regulatory Principles of Pluripotency: From the Ground State Up. *Cell Stem Cell* 15: 416-430

Hitchins MP (2015) Constitutional epimutation as a mechanism for cancer causality and heritability? *Nat Rev Cancer* 15: 625-634

Leitch HG, McEwen KR, Turp A, Encheva V, Carroll T, Grabole N, Mansfield W, Nashun B, Knezovich JG, Smith A *et al* (2013) Naive pluripotency is associated with global DNA hypomethylation. *Nat Struct Mol Biol* 20: 311-316

Loda A, Heard E (2019) Xist RNA in action: Past, present, and future. *PLoS Genet* 15: e1008333

Marks H, Kalkan T, Menafra R, Denissov S, Jones K, Hofemeister H, Nichols J, Kranz A, Stewart AF, Smith A *et al* (2012) The transcriptional and epigenomic foundations of ground state pluripotency. *Cell* 149: 590-604

Roulois D, Loo Yau H, Singhania R, Wang Y, Danesh A, Shen SY, Han H, Liang G, Jones PA, Pugh TJ *et al* (2015) DNA-Demethylating Agents Target Colorectal Cancer Cells by Inducing Viral Mimicry by Endogenous Transcripts. *Cell* 162: 961-973

Saunderson EA, Stepper P, Gomm JJ, Hoa L, Morgan A, Allen MD, Jones JL, Gribben JG, Jurkowski TP, Ficz G (2017) Hit-and-run epigenetic editing prevents senescence entry in primary breast cells from healthy donors. *Nat Commun* 8: 1450

Schulz EG, Meisig J, Nakamura T, Okamoto I, Sieber A, Picard C, Borensztein M, Saitou M, Bluthgen N, Heard E (2014) The Two Active X Chromosomes in Female ESCs Block Exit from the Pluripotent State by Modulating the ESC Signaling Network. *Cell Stem Cell* 14: 203-216

1st Revision - Editorial Decision 13th Dec 2021

Dear Dr Hackett, dear Dr Carlini,

Thank you for submitting your revised manuscript (EMBOJ-2021-108677) to The EMBO Journal, as well as your patience with our response. Your amended study was sent back to the two reviewers for re-evaluation, and we have received comments from both of them, which I enclose below.

The referees stated that their issues have been comprehensively resolved and they are now broadly in favour of publication, pending minor revision.

Thus, we are pleased to inform you that your manuscript has been accepted in principle for publication in The EMBO Journal.

Please address the remaining minor issues stated by referee #2 carefully by adjusting the data discussion and display. Further, we need you to consider a number of points related to formatting and data deposition as detailed below, which should be addressed at re-submission.

Please contact me at any time if you have additional questions related to below points.

As you might have noted on our web page, every paper at the EMBO Journal now includes a 'Synopsis', displayed on the html and freely accessible to all readers. The synopsis includes a 'model' figure as well as 2-5 one-short-sentence bullet points that summarize the article. I would appreciate if you could provide this figure and the bullet points.

Thank you for giving us the chance to consider your manuscript for The EMBO Journal. I look forward to your final revision.

Again, please contact me at any time if you need any help or have further questions.

Kind regards,

Daniel Klimmeck

Daniel Klimmeck PhD
Senior Editor
The EMBO Journal

Formatting changes required for the final revised version of the manuscript:

>> Please limit keywords to maximally five.

>> Add a separate 'Statistical analysis' section to your manuscript, detailing the algorithms applied.

>> Re-check statistical test information and display: Figure 1, 2, 6, EV2, EV4, EV5: t-test performed with n=2. Replicates and error bars: Figure 1, 2, 4, 5, 6, EV2, EV4, EV5: S.D: displayed for n=2

>> Check callouts for Figures 6G and Table 1 in the manuscript text.

>> Rescreen the manuscript text and Author Checklist for typos.

>> Adjust the 'Competing Interests' section to 'Conflict of Interest'.

>> Please introduce database hyperlinks in the data accessibility section.

Please remember: Digital image enhancement is acceptable practice, as long as it accurately represents the original data and conforms to community standards. If a figure has been subjected to significant electronic manipulation, this must be noted in the figure legend or in the 'Materials and Methods' section. The editors reserve the right to request original versions of figures and the original images that were used to assemble the figure.

Further information is available in our Guide For Authors: https://www.embopress.org/page/journal/14602075/authorguide

We realize that it is difficult to revise to a specific deadline. In the interest of protecting the conceptual advance provided by the work, we recommend a revision within 3 months (13th Mar 2022). Please discuss the revision progress ahead of this time with the editor if you require more time to complete the revisions. Use the link below to submit your revision:

Link Not Available

Referee #1:

In this revised manuscript, the authors have reasonably addressed all my previous comments. I therefore recommend publication of this manuscript.
Please, next time highlight the modified text in red. It was not always easy to track the changes in the text...

Referee #2:

The authors have addressed my comments satisfactorily. The choice of the experiments with the X-linked gene is pertinent, and overall, the authors have toned down most of their overstatements. The work is elegant and important and thus I am positive for publication of the work in EMBO.

I just have one minor point, which I hope the authors can make all efforts to correct/discuss properly. My remaining point relates to Figure 6, and the p53 data interpretation. Based on their data, it would seem to me that the p53 allele has no memory/has erased memory in fact by 4 days. It is quite surprising that in spite of this, the authors report some 'memory' in the chimera experiments in vivo. It would be important that the authors i) further clarify by text amendments and making these findings very explicit and ii) discuss potential implications of the differences obtained in the two models for their memory readout (cell culture and embryo).

Minor comment:
Figure 5G. The scale on the heatmap is marked as 2 to 2 (I presume that a 'minus' symbol is missing). The colour code used here is opposite to the one used in Figure 3C, which is perhaps not most adequate.

The authors performed the requested editorial changes.

2nd Revision - Editorial Decision **17th Jan 2022**

Dear Dr Hackett,

Thank you for submitting the revised version of your manuscript. I have now evaluated your amended manuscript and concluded that the remaining minor concerns have been sufficiently addressed.

Thus, I am pleased to inform you that your manuscript has been accepted for publication in the EMBO Journal.

Please note that it is EMBO Journal policy for the transcript of the editorial process (containing referee reports and your response letter) to be published as an online supplement to each paper. I would thus like to ask for your consent on keeping the additional referee figures included in this file.

Also, in case you might NOT want the transparent process file published at all, you will also need to inform us via email immediately. More information is available here: http://emboj.embopress.org/about#Transparent_Process

----

Please note that in order to be able to start the production process, our publisher will need and contact you regarding the following forms:

- PAGE CHARGE AUTHORISATION (For Articles and Resources)
http://onlinelibrary.wiley.com/journal/10.1002/(ISSN)1460-2075/homepage/tej_apc.pdf

- LICENCE TO PUBLISH (for non-Open Access)

Your article cannot be published until the publisher has received the appropriate signed license agreement. Once your article has been received by Wiley for production you will receive an email from Wiley's Author Services system, which will ask you to log in and will present them with the appropriate license for completion.

- LICENCE TO PUBLISH for OPEN ACCESS papers

Authors of accepted peer-reviewed original research articles may choose to pay a fee in order for their published article to be made freely accessible to all online immediately upon publication. The EMBO Open fee is fixed at $5,200 (+ VAT where applicable).

We offer two licenses for Open Access papers, CC-BY and CC-BY-NC-ND.
For more information on these licenses, please visit:
http://creativecommons.org/licenses/by-nc-nd/3.0/deed.en_US

- PAYMENT FOR OPEN ACCESS papers

You also need to complete our payment system for Open Access articles. Please follow this link and select EMBO Journal from the drop down list and then complete the payment process: https://authorservices.wiley.com/bauthor/onlineopen_order.asp

Should you be planning a Press Release on your article, please get in contact with embojournal@wiley.com as early as possible, in order to coordinate publication and release dates.

On a different note, I would like to alert you that EMBO Press is currently developing a new format for a video-synopsis of work published with us, which essentially is a short, author-generated film explaining the core findings in hand drawings, and, as we believe, can be very useful to increase visibility of the work. This has proven to offer a nice opportunity for exposure i.p. for the first author(s) of the study. Please see the following link for representative examples and their integration into the article web page:
https://www.embopress.org/video_synopses
https://www.embopress.org/doi/full/10.15252/embj.2019103932

Please let me know, should you be interested to engage in commissioning a similar video synopsis for your work. According operation instructions are available and intuitive.

Finally, we have noted that the submitted version of your article is also posted on the preprint platform bioRxiv. We would thus

appreciate if you could alert bioRxiv on the acceptance of this manuscript at The EMBO Journal in order to allow for an update of the entry status. Thank you in advance!

If you have any questions, please do not hesitate to call or email the Editorial Office.

Thank you again for this contribution to The EMBO Journal and congratulations on a successful publication! Please consider us again in the future for your most exciting work.

Kind regards,

Daniel Klimmeck

Daniel Klimmeck, PhD
Senior Editor
The EMBO Journal
EMBO
Postfach 1022-40
Meyerhofstrasse 1
D-69117 Heidelberg
contact@embojournal.org
Submit at: http://emboj.msubmit.net

EMBO PRESS

YOU MUST COMPLETE ALL CELLS WITH A PINK BACKGROUND

PLEASE NOTE THAT THIS CHECKLIST WILL BE PUBLISHED ALONGSIDE YOUR PAPER

Corresponding Author Name: Jamie Hackett
Journal Submitted to: EMBO Journal
Manuscript Number: EMBOJ-2021-108677

Reporting Checklist For Life Sciences Articles (Rev. June 2017)

This checklist is used to ensure good reporting standards and to improve the reproducibility of published results. These guidelines are consistent with the Principles and Guidelines for Reporting Preclinical Research issued by the NIH in 2014. Please follow the journal's authorship guidelines in preparing your manuscript.

A- Figures
1. Data
The data shown in figures should satisfy the following conditions:

- the data were obtained and processed according to the field's best practice and are presented to reflect the results of the experiments in an accurate and unbiased manner.
- figure panels include only data points, measurements or observations that can be compared to each other in a scientifically meaningful way.
- graphs include clearly labeled error bars for independent experiments and sample sizes. Unless justified, error bars should not be shown for technical replicates.
- if n< 5, the individual data points from each experiment should be plotted and any statistical test employed should be justified
- Source Data should be included to report the data underlying graphs. Please follow the guidelines set out in the author ship guidelines on Data Presentation.

2. Captions

Each figure caption should contain the following information, for each panel where they are relevant:

- a specification of the experimental system investigated (eg cell line, species name).
- the assay(s) and method(s) used to carry out the reported observations and measurements
- an explicit mention of the biological and chemical entity(ies) that are being measured.
- an explicit mention of the biological and chemical entity(ies) that are altered/varied/perturbed in a controlled manner.
- the exact sample size (n) for each experimental group/condition, given as a number, not a range;
- a description of the sample collection allowing the reader to understand whether the samples represent technical or biological replicates (including how many animals, litters, cultures, etc.).
- a statement of how many times the experiment shown was independently replicated in the laboratory.
- definitions of statistical methods and measures:
  - common tests, such as t-test (please specify whether paired vs. unpaired), simple χ2 tests, Wilcoxon and Mann-Whitney tests, can be unambiguously identified by name only, but more complex techniques should be described in the methods section;
  - are tests one-sided or two-sided?
  - are there adjustments for multiple comparisons?
  - exact statistical test results, e.g., P values = x but not P values < x;
  - definition of 'center values' as median or average;
  - definition of error bars as s.d. or s.e.m.

Any descriptions too long for the figure legend should be included in the methods section and/or with the source data.

In the pink boxes below, please ensure that the answers to the following questions are reported in the manuscript itself. Every question should be answered. If the question is not relevant to your research, please write NA (non applicable). We encourage you to include a specific subsection in the methods section for statistics, reagents, animal models and human subjects.

USEFUL LINKS FOR COMPLETING THIS FORM

http://www.antibodypedia.com
http://1degreebio.org
http://www.equator-network.org/reporting-guidelines/improving-bioscience-research-repor
http://grants.nih.gov/grants/olaw/olaw.htm
http://www.mrc.ac.uk/Ourresearch/Ethicsresearchguidance/Useofanimals/index.htm
http://ClinicalTrials.gov
http://www.consort-statement.org
http://www.consort-statement.org/checklists/view/32-consort/66-title
http://www.equator-network.org/reporting-guidelines/reporting-recommendations-for-tumo
http://datadryad.org
http://figshare.com
http://www.ncbi.nlm.nih.gov/gap
http://www.ebi.ac.uk/ega
http://biomodels.net/
http://biomodels.net/miriam/
http://jjj.biochem.sun.ac.za
https://osp.od.nih.gov/biosafety-biosecurity-and-emerging-biotechnology/
http://www.selectagents.gov/

B- Statistics and general methods

	Please fill out these boxes (Do not worry if you cannot see all your text once you press return)
1.a. How was the sample size chosen to ensure adequate power to detect a pre-specified effect size?	No specific measures were taken to predetermine sample size. RNA-seq, CUT&RUN and other molecular assays were generally performed in field-standard biological triplicate, with indpendent clonally derived cell lines as replicates. CRISPR screens were performed in biological duplicates, with extensive internal replications.
1.b. For animal studies, include a statement about sample size estimate even if no statistical methods were used.	For the only experiment to include animals, sample size was selected in order to provide statistical power for discovering proof of principle of a experiment without precedent, whilst minimising animal usage following 3R guidelines.
2. Describe inclusion/exclusion criteria if samples or animals were excluded from the analysis. Were the criteria pre-established?	N/A
3. Were any steps taken to minimize the effects of subjective bias when allocating animals/samples to treatment (e.g. randomization procedure)? If yes, please describe.	N/A
For animal studies, include a statement about randomization even if no randomization was used.	Groups of aggregated morula were randomly allocated to a recipient dam.
4.a. Were any steps taken to minimize the effects of subjective bias during group allocation or/and when assessing results (e.g. blinding of the investigator)? If yes please describe.	N/A
4.b. For animal studies, include a statement about blinding even if no blinding was done	Researchers were necessrily aware of which experimental group each animal belonged to. Analysis was performed via an unbiased quantative flow cytometry method.
5. For every figure, are statistical tests justified as appropriate?	YES
Do the data meet the assumptions of the tests (e.g., normal distribution)? Describe any methods used to assess it.	YES

Is there an estimate of variation within each group of data?	N/A
Is the variance similar between the groups that are being statistically compared?	N/A

C- Reagents

6. To show that antibodies were profiled for use in the system under study (assay and species), provide a citation, catalog number and/or clone number, supplementary information or reference to an antibody validation profile. e.g., Antibodypedia (see link list at top right), 1DegreeBio (see link list at top right).	For comprehensive listing, see Table EV 3
7. Identify the source of cell lines and report if they were recently authenticated (e.g., by STR profiling) and tested for mycoplasma contamination.	Cell lines were either derived freshly for this study and engineeered at low passage with CRISPR or taken from (Hackett et al., 2018). All lines were routinly tested (bi-monthly) for mycoplasma by outsourced independent qPCR assay and found to be negative.

* for all hyperlinks, please see the table at the top right of the document

D- Animal Models

8. Report species, strain, gender, age of animals and genetic modification status where applicable. Please detail housing and husbandry conditions and the source of animals.	N/A
9. For experiments involving live vertebrates, include a statement of compliance with ethical regulations and identify the committee(s) approving the experiments.	All experiemnts involving mice were carried out in accrodance with the approved protocol, license and guidelines by the laboratory animal management ethics committee of the European Molecular Biology Laboratory and Italian Ministry of Health.
10. We recommend consulting the ARRIVE guidelines (see link list at top right) (PLoS Biol. 8(6), e1000412, 2010) to ensure that other relevant aspects of animal studies are adequately reported. See author guidelines, under 'Reporting Guidelines'. See also: NIH (see link list at top right) and MRC (see link list at top right) recommendations. Please confirm compliance.	YES

E- Human Subjects

11. Identify the committee(s) approving the study protocol.	N/A
12. Include a statement confirming that informed consent was obtained from all subjects and that the experiments conformed to the principles set out in the WMA Declaration of Helsinki and the Department of Health and Human Services Belmont Report.	N/A
13. For publication of patient photos, include a statement confirming that consent to publish was obtained.	N/A
14. Report any restrictions on the availability (and/or on the use) of human data or samples.	N/A
15. Report the clinical trial registration number (at ClinicalTrials.gov or equivalent), where applicable.	N/A
16. For phase II and III randomized controlled trials, please refer to the CONSORT flow diagram (see link list at top right) and submit the CONSORT checklist (see link list at top right) with your submission. See author guidelines, under 'Reporting Guidelines'. Please confirm you have submitted this list.	N/A
17. For tumor marker prognostic studies, we recommend that you follow the REMARK reporting guidelines (see link list at top right). See author guidelines, under 'Reporting Guidelines'. Please confirm you have followed these guidelines.	N/A

F- Data Accessibility

18: Provide a "Data Availability" section at the end of the Materials & Methods, listing the accession codes for data generated in this study and deposited in a public database (e.g. RNA-Seq data: Gene Expression Omnibus GSE39462, Proteomics data: PRIDE PXD000208 etc.) Please refer to our author guidelines for 'Data Deposition'.<br><br>Data deposition in a public repository is mandatory for:<br>a. Protein, DNA and RNA sequences<br>b. Macromolecular structures<br>c. Crystallographic data for small molecules<br>d. Functional genomics data<br>e. Proteomics and molecular interactions	YES. All data derived from CRISPR screening, RNA-seq, Cut&Run-seq and ATAC-seq have been deposited in the publically available ArrayExpress database under the accession codes E-MTAB-10522, E-MTAB-10523, E-MTAB-10524, E-MTAB-10525.
19. Deposition is strongly recommended for any datasets that are central and integral to the study; please consider the journal's data policy. If no structured public repository exists for a given data type, we encourage the provision of datasets in the manuscript as a Supplementary Document (see author guidelines under 'Expanded View' or in unstructured repositories such as Dryad (see link list at top right) or Figshare (see link list at top right).	N/A
20. Access to human clinical and genomic datasets should be provided with as few restrictions as possible while respecting ethical obligations to the patients and relevant medical and legal issues. If practically possible and compatible with the individual consent agreement used in the study, such data should be deposited in one of the major public access-controlled repositories such as dbGAP (see link list at top right) or EGA (see link list at top right).	N/A
21. Computational models that are central and integral to a study should be shared without restrictions and provided in a machine-readable form. The relevant accession numbers or links should be provided. When possible, standardized format (SBML, CellML) should be used instead of scripts (e.g. MATLAB). Authors are strongly encouraged to follow the MIRIAM guidelines (see link list at top right) and deposit their model in a public database such as Biomodels (see link list at top right) or JWS Online (see link list at top right). If computer source code is provided with the paper, it should be deposited in a public repository or included in supplementary information.	N/A

G- Dual use research of concern

22. Could your study fall under dual use research restrictions? Please check biosecurity documents (see link list at top right) and list of select agents and toxins (APHIS/CDC) (see link list at top right). According to our biosecurity guidelines, provide a statement only if it could.	N/A
